# Supplementary material for: Gabapentin in pregnancy and the risk of adverse neonatal and maternal outcomes: A population-based cohort study nested in the US Medicaid Analytic eXtract dataset
Source: PLoS Med. 2020 Sep 1;17(9):e1003322. doi: 10.1371/journal.pmed.1003322 (PMC7462308; doi:10.1371/journal.pmed.1003322)
Supplement: S1 Table — (DOCX) [file pmed.1003322.s001.docx]

# S1 Table. Definitions for congenital malformations^1^

| **Malformation Group** | **ICD-9 Code** |
| --- | --- |
| 1. Central Nervous System defects | 740.xx-742.xx |
| 2. Eye anomalies | 743.xx (exclude if only 743.6x and 743.8x) |
| 3. Ear anomalies | 744.xx (exclude if only 744.1x, 744.21, 744.29, and 744.4x-744.9x) |
| 4. Cardiac malformations | 745.xx-746.xx, 747.0x-747.4x, 747.83 (exclude if only 745.5 AND preterm, 746.02 AND preterm, 746.4x, 746.6x, 746.99, 747.0x and preterm, 747.3 and preterm) |
| 5. Vascular (non-cardiac) malformations | 747.6x-747.9x (exclude if only 747.83) |
| 6. Respiratory malformations | 748.xx (do not count if only 748.1x) |
| 7. Oral cleft | 749.xx |
| 8. Gastrointestinal malformations | 750.xx-751.xx (do not count if only 750.0x, 750.1x, 750.50, 751.0x) |
| 9. Genital (male and female) malformations | 752.xx (do not count if only 752.42, 752.52) (in addition, do not count 752.5x if preterm) |
| 10. Urinary malformations | 753.xx (do not include if only 753.7x) |
| 11. Musculoskeletal malformations | 754.xx and 756.xx (do not count if only 754.3x, 754.81, 754.82, 756.2x) |
| 12. Limb defects | 755.xx (exclude if only 755.65) |
| 13. Other malformations | 757.xx; 759.xx (excl if only 757.2-757.6, 759.81-759.83) |

^1^Major malformations were defined on the basis of inpatient or outpatient ICD-9 diagnostic and procedural codes in the maternal (first month after delivery) or infant records (first three months after birth) records. We considered maternal records since Medicaid claims are sometimes recorded under the mother before the infant’s eligibility has been processed [Centers for Medicare & Medicaid Services. Medicaid Analytic eXtract (MAX) general information. MAX 1999-2005 state claims anomalies from the “2005 files” zipped file within the “MAX Data 2005 to 2008 general information, data dictionaries, data element lists, data anomalies, validation table measures and SAS loads zipped file. http://www.cms.gov/research-statistics-data-and-systems/computer-data-and-systems/medicaiddatasourcesgeninfo/maxgeneralinformation.html. Accessed December 23, 2018]. Congenital malformations were defined as the presence of any of 13 malformation groups (central nervous system, ear, eye, cardiac, other vascular, respiratory, oral cleft, gastro-intestinal, genital, urinary, musculoskeletal, limb, other). A specific malformation was deemed to be present if (i) an ICD-9 diagnosis for the specific malformation group was recorded on >1 date, (ii) a diagnosis on one date was accompanied by a relevant surgery or procedure code, or (iii) a diagnosis was present on one date and the infant died within 90 days from birth.
