# Supplementary material for: Gabapentin in pregnancy and the risk of adverse neonatal and maternal outcomes: A population-based cohort study nested in the US Medicaid Analytic eXtract dataset
Source: PLoS Med. 2020 Sep 1;17(9):e1003322. doi: 10.1371/journal.pmed.1003322 (PMC7462308; doi:10.1371/journal.pmed.1003322)
Supplement: S2 Table — NICUa, neonatal intensive care unit admission; SGA, small for gestational age. (DOCX) [file pmed.1003322.s002.docx]

# S2 Table. Definitions for preeclampsia, preterm birth, small for gestational age, and neonatal intensive care unit admission

| **Outcome** | **Definition** |
| --- | --- |
| Preeclampsia | An ICD-9 inpatient code for preeclampsia or eclampsia 642.4x-642.7x from the delivery hospitalization |
| Preterm birth | An ICD-9 code for preterm birth from delivery to delivery + 30 days: 644.21, 765.1-765.19, 765.21, 765.22, 765.23, 765.24, 765.0-765.09, 765.25, 765.26, 765.27, 765.28,  Other preterm ICD-9 codes from delivery to delivery + 30 days: 362.20, 362.22, 362.23, 362.24, 362.25, 362.26, 362.27, 644.20, 765.20, 776.6x, CPT-4 codes: 49491, 49492, 67229, 00836 |
| Small for gestational age | An ICD-9 code from delivery to delivery + 30 days: 656.5x, 764.0x, 764.1x, 764.9x |
| Neonatal intensive care unit admission | CPT-4 codes from delivery to delivery + 30 days: 99468, 99469, 4168 (only if in infant claims), 4169 (only if in infant claims), 99478, 99479, 99480, 99471, 99472, 99477, 99291 (only if in infant claims), 99292 (only if in infant claims), 99295, 99296, 99297 |
