# Supplementary material for: Gabapentin in pregnancy and the risk of adverse neonatal and maternal outcomes: A population-based cohort study nested in the US Medicaid Analytic eXtract dataset
Source: PLoS Med. 2020 Sep 1;17(9):e1003322. doi: 10.1371/journal.pmed.1003322 (PMC7462308; doi:10.1371/journal.pmed.1003322)
Supplement: S3 Table — PS, propensity score. (DOCX) [file pmed.1003322.s003.docx]

# S3 Table. Baseline characteristics of gabapentin exposed and unexposed women, before propensity score adjustment

| **Baseline characteristics** | **Reference: Unexposed (N=1,744,447)** | **Exposed  during T1 (N=4,642)** | ***St. Diff.*** | **Exposed early in pregnancy (N=3,745)** | ***St. Diff.*** | **Exposed  late in pregnancy (N=556)** | ***St. Diff.*** | **Exposed early and late in pregnancy (N=1,275)** | ***St. Diff.*** |
| --- | --- | --- | --- | --- | --- | --- | --- | --- | --- |
| Age at delivery |  |  |  |  |  |  |  |  |  |
| Age, mean (SD) | 24.3 (5.8) | 28.4 (6.0) | *0.69* | 28.1 (6.1) | *0.64* | 27.2 (5.8) | *0.50* | 28.9 (5.8) | *0.79* |
| Age categories, N (%) |  |  |  |  |  |  |  |  |  |
| <20 | 403,479 (23.1) | 285 (6.1) | *-0.50* | 249 (6.7) | *-0.48* | 49 (8.8) | *-0.40* | 68 (5.3) | *-0.53* |
| 20-24 | 597,207 (34.2) | 1,011 (21.8) | *-0.28* | 881 (23.5) | *-0.24* | 143 (25.7) | *-0.19* | 227 (17.8) | *-0.38* |
| 25-29 | 421,876 (24.2) | 1,480 (31.9) | *0.17* | 1,174 (31.4) | *0.16* | 187 (33.6) | *0.21* | 418 (32.8) | *0.19* |
| 30-34 | 208,127 (11.9) | 1,081 (23.3) | *0.30* | 838 (22.4) | *0.28* | 111 (20.0) | *0.22* | 334 (26.2) | *0.37* |
| 35-39 | 90,122 (5.2) | 604 (13.0) | *0.28* | 459 (12.3) | *0.25* | 52 (9.4) | *0.16* | 182 (14.3) | *0.31* |
| >39 | 23,636 (1.4) | 181 (3.9) | *0.16* | 144 (3.9) | *0.16* | 14 (2.5) | *0.08* | 46 (3.6) | *0.15* |
| Race/ethnicity, N (%) |  |  |  |  |  |  |  |  |  |
| White | 698,670 (40.1) | 3,210 (69.2) | *0.61* | 2,492 (66.5) | *0.55* | 334 (60.1) | *0.41* | 961 (75.4) | *0.77* |
| Black | 569,089 (32.6) | 615 (13.3) | *-0.47* | 561 (15.0) | *-0.42* | 116 (20.9) | *-0.27* | 125 (9.8) | *-0.58* |
| Hispanic | 250,779 (14.4) | 317 (6.8) | *-0.25* | 294 (7.9) | *-0.21* | 55 (9.9) | *-0.14* | 53 (4.2) | *-0.36* |
| Native American | 30,246 (1.7) | 109 (2.4) | *0.04* | 70 (1.9) | *0.01* | 11 (2.0) | *0.02* | 51 (4.0) | *0.14* |
| Asian | 68,499 (3.9) | 100 (2.2) | *-0.10* | 91 (2.4) | *-0.09* | <11 (1.6) | *-0.14* | 16 (1.3) | *-0.17* |
| Unknown | 38,314 (2.2) | 104 (2.2) | *0.00* | 77 (2.1) | *-0.01* | 13 (2.3) | *0.01* | 33 (2.6) | *0.03* |
| Other race | 88,850 (5.1) | 187 (4.0) | *-0.05* | 160 (4.3) | *-0.04* | 18 (3.2) | *-0.09* | 36 (2.8) | *-0.12* |
| Other^1^ | 225,909 (13.0) | 500 (10.8) | *-0.07* | 398 (10.6) | *-0.07* | 51 (9.2) | *-0.12* | 136 (10.7) | *-0.07* |
| Year of delivery, N (%) |  |  |  |  |  |  |  |  |  |
| 2000 | 1,614 (0.1) | <11 | *-0.02* | <11 | *-0.03* | <11 | *-0.04* | <11 | *0.00* |
| 2001 | 82,062 (4.7) | 120 (2.6) | *-0.11* | 98 (2.6) | *-0.11* | 17 (3.1) | *-0.09* | 26 (2.0) | *-0.15* |
| 2002 | 93,848 (5.4) | 164 (3.5) | *-0.09* | 144 (3.9) | *-0.07* | 29 (5.2) | *-0.01* | 30 (2.4) | *-0.16* |
| 2003 | 128,510 (7.4) | 272 (5.9) | *-0.06* | 225 (6.0) | *-0.05* | 30 (5.4) | *-0.08* | 77 (6.0) | *-0.05* |
| 2004 | 156,164 (9.0) | 322 (6.9) | *-0.07* | 264 (7.1) | *-0.07* | 32 (5.8) | *-0.12* | 93 (7.3) | *-0.06* |
| 2005 | 157,545 (9.0) | 308 (6.6) | *-0.09* | 250 (6.7) | *-0.09* | 37 (6.7) | *-0.09* | 78 (6.1) | *-0.11* |
| 2006 | 157,394 (9.0) | 268 (5.8) | *-0.12* | 232 (6.2) | *-0.11* | 38 (6.8) | *-0.08* | 59 (4.6) | *-0.17* |
| 2007 | 149,190 (8.6) | 273 (5.9) | *-0.10* | 221 (5.9) | *-0.10* | 26 (4.7) | *-0.16* | 73 (5.7) | *-0.11* |
| 2008 | 146,315 (8.4) | 306 (6.6) | *-0.07* | 268 (7.2) | *-0.05* | 36 (6.5) | *-0.07* | 75 (5.9) | *-0.10* |
| 2009 | 153,051 (8.8) | 424 (9.1) | *0.01* | 347 (9.3) | *0.02* | 43 (7.7) | *-0.04* | 121 (9.5) | *0.02* |
| 2010 | 161,022 (9.2) | 516 (11.1) | *0.06* | 422 (11.3) | *0.07* | 72 (13.0) | *0.12* | 127 (10.0) | *0.02* |
| 2011 | 170,232 (9.8) | 669 (14.4) | *0.14* | 523 (14.0) | *0.13* | 70 (12.6) | *0.09* | 206 (16.2) | *0.19* |
| 2012 | 145,089 (8.3) | 741 (16.0) | *0.24* | 567 (15.1) | *0.21* | 108 (19.4) | *0.33* | 213 (16.7) | *0.26* |
| 2013 | 42,411 (2.4) | 257 (5.5) | *0.16* | 183 (4.9) | *0.13* | 18 (3.2) | *0.05* | 96 (7.5) | *0.24* |
| Medicaid eligibility group, N (%) |  |  |  |  |  |  |  |  |  |
| 11-Individuals receiving cash assistance or eligible under section 1931 of the act-aged | <11 | <11 | *0.00* | <11 | *0.00* | <11 | *0.00* | <11 | *0.00* |
| 12-Individuals receiving cash assistance or eligible under section 1931 of the act - blind/disabled | 42,041 (2.4) | 425 (9.2) | *0.29* | 303 (8.1) | *0.26* | 37 (6.7) | *0.21* | 151 (11.8) | *0.37* |
| 14-Individuals receiving cash assistance or eligible under section 1931 of the act - children | 147,512 (8.5) | 78 (1.7) | *-0.31* | 77 (2.1) | *-0.29* | 19 (3.4) | *-0.21* | <11 | *-0.38* |
| 15-Individuals receiving cash assistance or eligible under section 1931 of the act - adults | 811,607 (46.5) | 2,631 (56.7) | *0.20* | 2,135 (57.0) | *0.21* | 317 (57.0) | *0.21* | 715 (56.1) | *0.19* |
| 16-Individuals receiving cash assistance or eligible under section 1931 -u children | 1,090 (0.1) | <11 | *-0.02* | <11 | *-0.02* | <11 | *-0.04* | <11 | *-0.04* |
| 17-Individuals receiving cash assistance or eligible under section 1931 - u adults | 11,285 (0.7) | 34 (0.7) | *0.01* | 30 (0.8) | *0.02* | <11 | *0.05* | <11 | *0.00* |
| 22-Medically needy - blind/disabled | 822 (0.1) | 14 (0.3) | *0.06* | 12 (0.3) | *0.06* | <11 | *0.04* | <11 | *0.05* |
| 24-Medically needy - children | 33,240 (1.9) | 29 (0.6) | *-0.11* | 27 (0.7) | *-0.10* | <11 | *-0.07* | <11 | *-0.14* |
| 25-Medically needy - adults | 127,640 (7.3) | 216 (4.7) | *-0.11* | 182 (4.9) | *-0.10* | 28 (5.0) | *-0.09* | 52 (4.1) | *-0.14* |
| 31-Poverty related eligibles - aged | <11 | <11 | *0.00* | <11 | *0.00* | <11 | *0.00* | <11 | *0.00* |
| 32-Poverty related eligibles - blind/disabled | 652 (0.0) | 16 (0.3) | *0.07* | 11 (0.3) | *0.06* | <11 | *0.09* | <11 | *0.08* |
| 34-Poverty related eligibles - children | 142,223 (8.2) | 115 (2.5) | *-0.26* | 95 (2.5) | *-0.25* | 14 (2.5) | *-0.25* | 30 (2.4) | *-0.26* |
| 35-Poverty related eligibles - adults | 137,134 (7.9) | 353 (7.6) | *-0.01* | 278 (7.4) | *-0.02* | 44 (7.9) | *0.00* | 104 (8.2) | *0.01* |
| 3a-Poverty related eligibles - adults | 268 (0.0) | <11 | *0.00* | <11 | *0.01* | <11 | *-0.02* | <11 | *-0.02* |
| 42-Other eligibles - blind/disabled | 1,993 (0.1) | 36 (0.8) | *0.10* | 29 (0.8) | *0.10* | <11 | *0.07* | <11 | *0.09* |
| 44-Other eligibles - children | 27,534 (1.6) | 29 (0.6) | *-0.09* | 26 (0.7) | *-0.08* | <11 | *-0.06* | <11 | *-0.11* |
| 45-Other eligibles - adults | 169,313 (9.7) | 449 (9.7) | *0.00* | 368 (9.8) | *0.00* | 54 (9.7) | *0.00* | 121 (9.5) | *-0.01* |
| 48-Other eligibles - foster care children | 11,501 (0.7) | <11 | *-0.07* | <11 | *-0.07* | <11 | *-0.07* | <11 | *-0.06* |
| 52-Section 1115 demonstration Medicaid expansion | 132 (0.0) | <11 | *0.02* | <11 | *0.03* | <11 | *-0.01* | <11 | *-0.01* |
| 54-Section 1115 demonstration Medicaid expansion | 3,078 (0.2) | <11 | *-0.06* | <11 | *-0.05* | <11 | *-0.06* | <11 | *-0.06* |
| 55-Section 1115 demonstration Medicaid expansion | 75,370 (4.3) | 203 (4.4) | *0.00* | 159 (4.3) | *0.00* | 18 (3.2) | *-0.06* | 54 (4.2) | *0.00* |
| Multiple gestation, N (%) | 59,636 (3.4) | 219 (4.7) | *0.07* | 177 (4.7) | *0.07* | 34 (6.1) | *0.13* | 59 (4.6) | *0.06* |
| **Labeled indications, N (%)** |  |  |  |  |  |  |  |  |  |
| Epilepsy or seizures | 11,861 (0.7) | 347 (7.5) | *0.35* | 193 (5.2) | *0.27* | 36 (6.5) | *0.32* | 179 (14.0) | *0.53* |
| Neuropathic pain | 21,701 (1.2) | 1,116 (24.0) | *0.73* | 918 (24.5) | *0.74* | 65 (11.7) | *0.43* | 264 (20.7) | *0.65* |
| Restless legs syndrome | 345 (0.0) | 37 (0.8) | *0.12* | 24 (0.6) | *0.11* | <11 | *0.05* | 15 (1.2) | *0.15* |
| **Pain conditions, N (%)** |  |  |  |  |  |  |  |  |  |
| Fibromyalgia | 14,545 (0.8) | 397 (8.6) | *0.37* | 332 (8.9) | *0.38* | 35 (6.3) | *0.30* | 91 (7.1) | *0.33* |
| Arthritis, arthropathies and musculoskeletal pain | 146,564 (8.4) | 1,862 (40.1) | *0.80* | 1,518 (40.5) | *0.81* | 169 (30.4) | *0.58* | 482 (37.8) | *0.74* |
| Back and neck pain | 135,116 (7.8) | 2,099 (45.2) | *0.94* | 1,681 (44.9) | *0.93* | 162 (29.1) | *0.57* | 567 (44.5) | *0.92* |
| Migraine or headache | 124,860 (7.2) | 1,194 (25.7) | *0.52* | 975 (26.0) | *0.52* | 111 (20.0) | *0.38* | 313 (24.6) | *0.49* |
| Other pain conditions^2^ | 18,052 (1.0) | 573 (12.3) | *0.46* | 435 (11.6) | *0.45* | 34 (6.1) | *0.28* | 182 (14.3) | *0.51* |
| **Psychiatric conditions, N (%)** |  |  |  |  |  |  |  |  |  |
| Depression | 106,658 (6.1) | 1,261 (27.2) | *0.59* | 1,013 (27.1) | *0.59* | 98 (17.6) | *0.36* | 336 (26.4) | *0.57* |
| Bipolar disorder | 21,146 (1.2) | 573 (12.3) | *0.45* | 434 (11.6) | *0.43* | 42 (7.6) | *0.31* | 174 (13.7) | *0.49* |
| Anxiety | 64,514 (3.7) | 1,047 (22.6) | *0.58* | 798 (21.3) | *0.55* | 87 (15.7) | *0.41* | 332 (26.0) | *0.66* |
| Attention deficit hyperactivity disorder | 16,699 (1.0) | 145 (3.1) | *0.15* | 105 (2.8) | *0.14* | 18 (3.2) | *0.16* | 51 (4.0) | *0.20* |
| Psychosis | 5,716 (0.3) | 105 (2.3) | *0.17* | 85 (2.3) | *0.17* | <11 | *0.05* | 29 (2.3) | *0.17* |
| Other psychiatric disorders | 34,326 (2.0) | 352 (7.6) | *0.27* | 292 (7.8) | *0.27* | 24 (4.3) | *0.13* | 80 (6.3) | *0.22* |
| **Other maternal conditions, N (%)** |  |  |  |  |  |  |  |  |  |
| Hypertension | 39,100 (2.2) | 419 (9.0) | *0.30* | 318 (8.5) | *0.28* | 40 (7.2) | *0.24* | 132 (10.4) | *0.34* |
| Diabetes | 27,469 (1.6) | 370 (8.0) | *0.30* | 295 (7.9) | *0.30* | 28 (5.0) | *0.19* | 104 (8.2) | *0.31* |
| Renal disease | 5,755 (0.3) | 78 (1.7) | *0.14* | 54 (1.4) | *0.12* | <11 | *0.05* | 25 (2.0) | *0.15* |
| Chronic fatigue syndrome | 53,027 (3.0) | 400 (8.6) | *0.24* | 336 (9.0) | *0.25* | 35 (6.3) | *0.15* | 85 (6.7) | *0.17* |
| Nausea and vomiting | 208,372 (11.9) | 971 (20.9) | *0.24* | 836 (22.3) | *0.28* | 93 (16.7) | *0.14* | 211 (16.6) | *0.13* |
| Sleep disorder | 12,439 (0.7) | 257 (5.5) | *0.28* | 197 (5.3) | *0.27* | 22 (4.0) | *0.22* | 74 (5.8) | *0.29* |
| **Lifestyle factors, N (%)** |  |  |  |  |  |  |  |  |  |
| Obesity or overweight | 39,697 (2.3) | 274 (5.9) | *0.18* | 213 (5.7) | *0.18* | 29 (5.2) | *0.16* | 75 (5.9) | *0.18* |
| Smoking | 68,294 (3.9) | 729 (15.7) | *0.40* | 557 (14.9) | *0.38* | 60 (10.8) | *0.27* | 225 (17.7) | *0.45* |
| Drug abuse or dependence | 18,618 (1.1) | 245 (5.3) | *0.24* | 201 (5.4) | *0.25* | 20 (3.6) | *0.17* | 56 (4.4) | *0.21* |
| Alcohol abuse or dependence | 7,271 (0.4) | 107 (2.3) | *0.16* | 89 (2.4) | *0.17* | <11 | *-0.01* | 24 (1.9) | *0.14* |
| **Concomitant use of medications, N (%)** |  |  |  |  |  |  |  |  |  |
| Anticonvulsants prior to pregnancy |  |  |  |  |  |  |  |  |  |
| Carbamazepine | 2,070 (0.1) | 78 (1.7) | *0.17* | 32 (0.9) | *0.11* | <11 | *0.09* | 52 (4.1) | *0.28* |
| Phenytoin | 1,713 (0.1) | 69 (1.5) | *0.16* | 37 (1.0) | *0.12* | <11 | *0.14* | 34 (2.7) | *0.22* |
| Topiramate | 5,483 (0.3) | 165 (3.6) | *0.24* | 127 (3.4) | *0.23* | <11 | *0.09* | 45 (3.5) | *0.24* |
| Valproate | 5,009 (0.3) | 119 (2.6) | *0.19* | 87 (2.3) | *0.18* | <11 | *0.12* | 42 (3.3) | *0.23* |
| Other anticonvulsants | 10,138 (0.6) | 304 (6.6) | *0.33* | 208 (5.6) | *0.29* | 30 (5.4) | *0.29* | 120 (9.4) | *0.41* |
| Anticonvulsants in first trimester |  |  |  |  |  |  |  |  |  |
| Carbamazepine | 1,671 (0.1) | 74 (1.6) | *0.16* | 29 (0.8) | *0.10* | <11 | *0.06* | 51 (4.0) | *0.28* |
| Phenytoin | 1,484 (0.1) | 55 (1.2) | *0.14* | 31 (0.8) | *0.11* | <11 | *0.12* | 26 (2.0) | *0.19* |
| Topiramate | 3,342 (0.2) | 158 (3.4) | *0.24* | 122 (3.3) | *0.24* | <11 | *0.08* | 47 (3.7) | *0.26* |
| Valproate | 3,007 (0.2) | 87 (1.9) | *0.17* | 53 (1.4) | *0.14* | <11 | *0.13* | 39 (3.1) | *0.23* |
| Other anticonvulsants | 8,030 (0.5) | 288 (6.2) | *0.32* | 205 (5.5) | *0.30* | 20 (3.6) | *0.22* | 104 (8.2) | *0.39* |
| Opioids and opioid-related treatment prior to pregnancy |  |  |  |  |  |  |  |  |  |
| Codeine | 57,930 (3.3) | 348 (7.5) | *0.19* | 280 (7.5) | *0.18* | 39 (7.0) | *0.17* | 96 (7.5) | *0.19* |
| Hydrocodone | 154,071 (8.8) | 1,644 (35.4) | *0.68* | 1,329 (35.5) | *0.68* | 181 (32.6) | *0.61* | 458 (35.9) | *0.69* |
| Oxycodone | 39,297 (2.3) | 738 (15.9) | *0.49* | 585 (15.6) | *0.48* | 62 (11.2) | *0.36* | 217 (17.0) | *0.52* |
| Tramadol | 32,150 (1.8) | 715 (15.4) | *0.50* | 537 (14.3) | *0.47* | 55 (9.9) | *0.35* | 221 (17.3) | *0.55* |
| Other opioids | 4,777 (0.3) | 230 (5.0) | *0.30* | 174 (4.7) | *0.29* | 22 (4.0) | *0.26* | 72 (5.7) | *0.32* |
| Buprenorphine | 2,603 (0.2) | 112 (2.4) | *0.20* | 72 (1.9) | *0.18* | <11 | *0.13* | 46 (3.6) | *0.26* |
| Opioid antagonists | 2,583 (0.2) | 113 (2.4) | *0.20* | 72 (1.9) | *0.18* | <11 | *0.12* | 45 (3.5) | *0.25* |
| Morphine equivalents, mg, mean (SD) | 58.7 (465.7) | 960.6 (2,200.6) | *0.57* | 905.5 (2,139.0) | *0.55* | 591.4 (1,647.2) | *0.44* | 1,153.7 (2,427.8) | *0.63* |
| Opioids and opioid-related treatment in first trimester |  |  |  |  |  |  |  |  |  |
| Codeine | 54,340 (3.1) | 418 (9.0) | *0.25* | 351 (9.4) | *0.26* | 44 (7.9) | *0.21* | 100 (7.8) | *0.21* |
| Hydrocodone | 99,899 (5.7) | 1,505 (32.4) | *0.72* | 1,195 (31.9) | *0.71* | 159 (28.6) | *0.64* | 442 (34.7) | *0.77* |
| Oxycodone | 20,474 (1.2) | 642 (13.8) | *0.49* | 503 (13.4) | *0.48* | 59 (10.6) | *0.41* | 202 (15.8) | *0.54* |
| Tramadol | 17,044 (1.0) | 591 (12.7) | *0.48* | 463 (12.4) | *0.47* | 53 (9.5) | *0.39* | 170 (13.3) | *0.49* |
| Other opioids | 3,663 (0.2) | 230 (5.0) | *0.30* | 171 (4.6) | *0.29* | 20 (3.6) | *0.25* | 84 (6.6) | *0.36* |
| Buprenorphine | 2,920 (0.2) | 140 (3.0) | *0.23* | 89 (2.4) | *0.20* | <11 | *0.13* | 57 (4.5) | *0.29* |
| Opioid antagonists^3^ | 2,433 (0.1) | 116 (2.5) | *0.21* | 73 (2.0) | *0.18* | <11 | *0.11* | 47 (3.7) | *0.26* |
| Morphine equivalents, mg, mean (SD) | 50.6 (544.4) | 1,064.4 (2,662.4) | *0.53* | 978.3 (2,532.6) | *0.51* | 732.2 (2,228.5) | *0.42* | 1,395.5 (3,128.5) | *0.60* |
| Other medications |  |  |  |  |  |  |  |  |  |
| Acetaminophen | 294,888 (16.9) | 2,441 (52.6) | *0.81* | 1,974 (52.7) | *0.81* | 269 (48.4) | *0.71* | 684 (53.7) | *0.83* |
| NSAIDs | 289,853 (16.6) | 2,051 (44.2) | *0.63* | 1,717 (45.9) | *0.66* | 219 (39.4) | *0.52* | 513 (40.2) | *0.54* |
| Triptans | 18,626 (1.1) | 349 (7.5) | *0.32* | 282 (7.5) | *0.32* | 25 (4.5) | *0.21* | 94 (7.4) | *0.32* |
| Antidepressants | 153,401 (8.8) | 2,595 (55.9) | *1.17* | 2,036 (54.4) | *1.12* | 184 (33.1) | *0.63* | 714 (56.0) | *1.17* |
| Benzodiazepines | 53,817 (3.1) | 1,275 (27.5) | *0.72* | 981 (26.2) | *0.69* | 124 (22.3) | *0.60* | 399 (31.3) | *0.81* |
| Other anxiolytics | 7,054 (0.4) | 203 (4.4) | *0.26* | 158 (4.2) | *0.26* | 16 (2.9) | *0.20* | 53 (4.2) | *0.25* |
| Other hypnotics | 61,638 (3.5) | 904 (19.5) | *0.52* | 707 (18.9) | *0.50* | 70 (12.6) | *0.34* | 269 (21.1) | *0.55* |
| Barbiturates | 17,280 (1.0) | 271 (5.8) | *0.27* | 210 (5.6) | *0.26* | 29 (5.2) | *0.25* | 89 (7.0) | *0.31* |
| Antipsychotics | 23,404 (1.3) | 761 (16.4) | *0.55* | 603 (16.1) | *0.54* | 58 (10.4) | *0.39* | 203 (15.9) | *0.54* |
| Stimulants | 15,088 (0.9) | 310 (6.7) | *0.31* | 209 (5.6) | *0.27* | 31 (5.6) | *0.27* | 117 (9.2) | *0.39* |
| Antihypertensive medications | 47,603 (2.7) | 739 (15.9) | *0.47* | 555 (14.8) | *0.44* | 58 (10.4) | *0.31* | 229 (18.0) | *0.52* |
| Non-insulin antidiabetic medications | 14,972 (0.9) | 215 (4.6) | *0.23* | 171 (4.6) | *0.23* | 13 (2.3) | *0.12* | 58 (4.6) | *0.23* |
| Insulin | 14,440 (0.8) | 246 (5.3) | *0.26* | 184 (4.9) | *0.25* | 17 (3.1) | *0.16* | 78 (6.1) | *0.29* |
| Corticosteroids | 204,556 (11.7) | 1,373 (29.6) | *0.45* | 1,124 (30.0) | *0.46* | 134 (24.1) | *0.33* | 349 (27.4) | *0.40* |
| Fluconazole | 75,606 (4.3) | 434 (9.4) | *0.20* | 359 (9.6) | *0.21* | 45 (8.1) | *0.16* | 116 (9.1) | *0.19* |
| Other teratogenic agents^4^ | 50,414 (2.9) | 182 (3.9) | *0.06* | 153 (4.1) | *0.07* | 22 (4.0) | *0.06* | 48 (3.8) | *0.05* |
| **Markers of burden of disease** |  |  |  |  |  |  |  |  |  |
| Obstetric Comorbidity Index^5^, mean (SD) | 0.8 (1.4) | 1.7 (2.0) | *0.52* | 1.6 (1.9) | *0.48* | 1.6 (1.8) | *0.45* | 1.9 (2.1) | *0.60* |
| Number of distinct filled prescriptions, mean (SD) | 1.7 (2.4) | 6.1 (4.4) | *1.23* | 5.9 (4.3) | *1.20* | 4.5 (4.1) | *0.82* | 6.3 (4.5) | *1.26* |
| Number of diagnoses, mean (SD) | 2.7 (3.2) | 6.6 (5.3) | *0.89* | 6.6 (5.2) | *0.89* | 5.4 (5.2) | *0.61* | 6.6 (5.5) | *0.86* |
| Number of outpatient physician visits, mean (SD) | 2.1 (3.5) | 6.2 (7.4) | *0.70* | 6.1 (7.5) | *0.69* | 4.7 (6.9) | *0.47* | 6.0 (6.7) | *0.72* |
| Patients hospitalized, N (%) | 62,587 (3.6) | 305 (6.6) | *0.14* | 235 (6.3) | *0.12* | 33 (5.9) | *0.11* | 91 (7.1) | *0.16* |
| Number of hospitalizations, mean (SD) | 0.0 (0.2) | 0.1 (0.3) | *0.15* | 0.1 (0.3) | *0.14* | 0.1 (0.3) | *0.12* | 0.1 (0.3) | *0.17* |
| Number of days hospitalized, mean (SD) | 0.1 (1.1) | 0.4 (2.2) | *0.15* | 0.4 (2.3) | *0.14* | 0.3 (1.6) | *0.11* | 0.4 (1.8) | *0.17* |
| Number of emergency room visits, mean (SD) | 0.3 (0.9) | 0.9 (1.7) | *0.45* | 0.9 (1.7) | *0.45* | 0.8 (1.5) | *0.38* | 0.9 (1.6) | *0.46* |
| T1: first trimester; St. Diff.: Standardized differences, i.e., the difference in means or proportions divided by the pooled standard deviation [Austin PC. Balance diagnostics for comparing the distribution of baseline covariates between treatment groups in propensity-score matched samples. Statistics in medicine 2009;28:3083-107]; SD: standard deviation  Maternal comorbidities and concomitant medication use were measured during the 3 months before pregnancy through the end of the first trimester. Measures of healthcare intensity (e.g., number of medical visits) were measured only during the 3-month period before pregnancy, in order for these not to be affected by early awareness of possible pregnancy complications. ^1^Other race includes Asian, Native American, Other, and Unknown ^2^ Other pain conditions include osteoarthritis, rheumatoid arthritis, generalized pain, pain disorders related to psychological factors, and pain not elsewhere classified ^3^ Opioid antagonists include naloxone and naltrexone ^4^ Other teratogenic agents include methimazole, danazol, propylthiouracil, and progestins ^5^ The obstetric comorbidity index predicts severe maternal morbidity. The range for the maternal comorbidity index is 0 to 45, with lower values associated with lower burden of maternal illness and higher values associated with higher burden of maternal illness [Bateman BT, Mhyre JM, Hernandez-Diaz S, Huybrechts KF, Fischer MA, Creanga AA, Callaghan WM, Gagne JJ. Development of a comorbidity index for use in obstetric patients. Obstet Gynecol. 2013;122:957-65] In accordance with the data use agreement, we do not report information for frequency cells with less than 11 cases. These are noted as <11 | | | | | | | | | |
