# Supplementary material for: Gabapentin in pregnancy and the risk of adverse neonatal and maternal outcomes: A population-based cohort study nested in the US Medicaid Analytic eXtract dataset
Source: PLoS Med. 2020 Sep 1;17(9):e1003322. doi: 10.1371/journal.pmed.1003322 (PMC7462308; doi:10.1371/journal.pmed.1003322)
Supplement: S4 Table — PS, propensity score. (DOCX) [file pmed.1003322.s004.docx]

# S4 Table. Baseline characteristics of gabapentin exposed and unexposed women, after propensity score adjustment

| **Baseline characteristics** | **Reference: Unexposed (N=1,728,052)** | **Exposed  during T1 (N=4,642)** | ***St. Diff.*** | **Reference: Unexposed (N=1,728,347)** | **Exposed early in pregnancy (N=3,745)** | ***St. Diff.*** | **Reference: Unexposed (N=1,736,786)** | **Exposed  late in pregnancy (N=555)** | ***St. Diff.*** | **Reference: Unexposed (N=1,618,836)** | **Exposed early and late in pregnancy (N = 1,275)** | ***St. Diff.*** |
| --- | --- | --- | --- | --- | --- | --- | --- | --- | --- | --- | --- | --- |
| Age at delivery |  |  |  |  |  |  |  |  |  |  |  |  |
| Age, mean (SD) | 28.3 (5.9) | 28.4 (6.0) | *0.00* | 28.1 (5.9) | 28.1 (6.1) | *0.00* | 27.2 (5.8) | 27.2 (5.8) | *0.00* | 28.9 (5.7) | 28.9 (5.8) | *0.01* |
| Age categories, N (%) |  |  |  |  |  |  |  |  |  |  |  |  |
| <20 | 92,517 (5.4) | 285 (6.1) | *0.03* | 100,890 (5.8) | 249 (6.7) | *0.03* | 145,132 (8.4) | 49 (8.8) | *0.02* | 72,556 (4.5) | 68 (5.3) | *0.04* |
| 20-24 | 375,521 (21.7) | 1,011 (21.8) | *0.00* | 406,367 (23.5) | 881 (23.5) | *0.00* | 438,865 (25.3) | 143 (25.8) | *0.01* | 281,300 (17.4) | 227 (17.8) | *0.01* |
| 25-29 | 565,503 (32.7) | 1,480 (31.9) | *-0.02* | 554,968 (32.1) | 1,174 (31.4) | *-0.02* | 592,910 (34.1) | 187 (33.7) | *-0.01* | 547,979 (33.9) | 418 (32.8) | *-0.02* |
| 30-34 | 407,016 (23.6) | 1,081 (23.3) | *-0.01* | 390,706 (22.6) | 838 (22.4) | *-0.01* | 350,416 (20.2) | 111 (20.0) | *0.00* | 431,924 (26.7) | 334 (26.2) | *-0.01* |
| 35-39 | 220,758 (12.8) | 604 (13.0) | *0.01* | 209,388 (12.1) | 459 (12.3) | *0.00* | 165,238 (9.5) | 51 (9.2) | *-0.01* | 226,921 (14.0) | 182 (14.3) | *0.01* |
| >39 | 66,738 (3.9) | 181 (3.9) | *0.00* | 66,026 (3.8) | 144 (3.9) | *0.00* | 44,226 (2.6) | 14 (2.5) | *0.00* | 58,156 (3.6) | 46 (3.6) | *0.00* |
| Race/ethnicity, N (%) |  |  |  |  |  |  |  |  |  |  |  |  |
| White | 1,230,928 (71.2) | 3,210 (69.2) | *-0.05* | 1,183,872 (68.5) | 2,492 (66.5) | *-0.04* | 1,058,595 (61.0) | 333 (60.0) | *-0.02* | 1,257,482 (77.7) | 961 (75.4) | *-0.05* |
| Black | 209,657 (12.1) | 615 (13.3) | *0.03* | 240,684 (13.9) | 561 (15.0) | *0.03* | 356,833 (20.6) | 116 (20.9) | *0.01* | 139,413 (8.6) | 125 (9.8) | *0.04* |
| Hispanic | 108,575 (6.3) | 317 (6.8) | *0.02* | 126,239 (7.3) | 294 (7.9) | *0.02* | 165,866 (9.6) | 55 (9.9) | *0.01* | 58,508 (3.6) | 53 (4.2) | *0.03* |
| Native American | 39,326 (2.3) | 109 (2.4) | *0.00* | 31,982 (1.9) | 70 (1.9) | *0.00* | 35,094 (2.0) | 11 (2.0) | *0.00* | 62,060 (3.8) | 51 (4.0) | *0.01* |
| Asian | 34,510 (2.0) | 100 (2.2) | *0.01* | 39,464 (2.3) | 91 (2.4) | *0.01* | 25,621 (1.5) | <11 | *0.01* | 16,356 (1.0) | 16 (1.3) | *0.02* |
| Unknown | 38,500 (2.2) | 104 (2.2) | *0.00* | 35,285 (2.0) | 77 (2.1) | *0.00* | 39,579 (2.3) | 13 (2.3) | *0.00* | 41,635 (2.6) | 33 (2.6) | *0.00* |
| Other race | 66,557 (3.9) | 187 (4.0) | *0.01* | 70,821 (4.1) | 160 (4.3) | *0.01* | 55,198 (3.2) | 18 (3.2) | *0.00* | 43,383 (2.7) | 36 (2.8) | *0.01* |
| Other^1^ | 178,893 (10.4) | 500 (10.8) | *0.01* | 177,552 (10.3) | 398 (10.6) | *0.01* | 155,492 (9.0) | 51 (9.2) | *0.01* | 163,433 (10.1) | 136 (10.7) | *0.02* |
| Year of delivery, N (%) |  |  |  |  |  |  |  |  |  |  |  |  |
| 2000 | 762 (0.0) | <11 | *0.00* | 445 (0.0) | <11 | *0.00* | <11 | <11 | *.* | 1,403 (0.1) | <11 | *0.00* |
| 2001 | 43,917 (2.5) | 120 (2.6) | *0.00* | 44,081 (2.6) | 98 (2.6) | *0.00* | 51,276 (3.0) | 17 (3.1) | *0.01* | 31,147 (1.9) | 26 (2.0) | *0.01* |
| 2002 | 61,428 (3.6) | 164 (3.5) | *0.00* | 67,130 (3.9) | 144 (3.9) | *0.00* | 91,475 (5.3) | 29 (5.2) | *0.00* | 36,487 (2.3) | 30 (2.4) | *0.01* |
| 2003 | 103,630 (6.0) | 272 (5.9) | *-0.01* | 106,293 (6.2) | 225 (6.0) | *-0.01* | 90,744 (5.2) | 30 (5.4) | *0.01* | 99,783 (6.2) | 77 (6.0) | *-0.01* |
| 2004 | 121,816 (7.1) | 322 (6.9) | *0.00* | 123,153 (7.1) | 264 (7.1) | *0.00* | 97,121 (5.6) | 32 (5.8) | *0.01* | 119,852 (7.4) | 93 (7.3) | *0.00* |
| 2005 | 113,971 (6.6) | 308 (6.6) | *0.00* | 113,637 (6.6) | 250 (6.7) | *0.00* | 111,744 (6.4) | 37 (6.7) | *0.01* | 97,340 (6.0) | 78 (6.1) | *0.00* |
| 2006 | 99,870 (5.8) | 268 (5.8) | *0.00* | 106,891 (6.2) | 232 (6.2) | *0.00* | 117,546 (6.8) | 38 (6.9) | *0.00* | 75,003 (4.6) | 59 (4.6) | *0.00* |
| 2007 | 102,299 (5.9) | 273 (5.9) | *0.00* | 102,038 (5.9) | 221 (5.9) | *0.00* | 81,395 (4.7) | 26 (4.7) | *0.00* | 93,208 (5.8) | 73 (5.7) | *0.00* |
| 2008 | 114,036 (6.6) | 306 (6.6) | *0.00* | 122,770 (7.1) | 268 (7.2) | *0.00* | 112,098 (6.5) | 36 (6.5) | *0.00* | 97,646 (6.0) | 75 (5.9) | *-0.01* |
| 2009 | 158,831 (9.2) | 424 (9.1) | *0.00* | 160,461 (9.3) | 347 (9.3) | *0.00* | 134,350 (7.7) | 43 (7.8) | *0.00* | 155,358 (9.6) | 121 (9.5) | *0.00* |
| 2010 | 193,707 (11.2) | 516 (11.1) | *0.00* | 197,472 (11.4) | 422 (11.3) | *0.00* | 227,538 (13.1) | 72 (13.0) | *0.00* | 163,676 (10.1) | 127 (10.0) | *0.00* |
| 2011 | 249,871 (14.5) | 669 (14.4) | *0.00* | 241,937 (14.0) | 523 (14.0) | *0.00* | 221,025 (12.7) | 70 (12.6) | *0.00* | 264,067 (16.3) | 206 (16.2) | *0.00* |
| 2012 | 271,909 (15.7) | 741 (16.0) | *0.01* | 259,863 (15.0) | 567 (15.1) | *0.00* | 343,537 (19.8) | 107 (19.3) | *-0.01* | 272,892 (16.9) | 213 (16.7) | *0.00* |
| 2013 | 92,003 (5.3) | 257 (5.5) | *0.01* | 82,175 (4.8) | 183 (4.9) | *0.01* | 56,937 (3.3) | 18 (3.2) | *0.00* | 110,974 (6.9) | 96 (7.5) | *0.03* |
| Medicaid eligibility group, N (%) |  |  |  |  |  |  |  |  |  |  |  |  |
| 11-Individuals receiving cash assistance or eligible under section 1931 of the act-aged | <11 | <11 | *0.00* | <11 | <11 | *0.00* | <11 | <11 | *0.00* | <11 | <11 | *0.00* |
| 12-Individuals receiving cash assistance or eligible under section 1931 of the act - blind/disabled | 158,108 (9.2) | 425 (9.2) | *0.00* | 138,980 (8.0) | 303 (8.1) | *0.00* | 111,551 (6.4) | 37 (6.7) | *0.01* | 187,594 (11.6) | 151 (11.8) | *0.01* |
| 14-Individuals receiving cash assistance or eligible under section 1931 of the act - children | 23,903 (1.4) | 78 (1.7) | *0.02* | 29,933 (1.7) | 77 (2.1) | *0.02* | 55,331 (3.2) | 19 (3.4) | *0.01* | 7,760 (0.5) | <11 | *0.03* |
| 15-Individuals receiving cash assistance or eligible under section 1931 of the act - adults | 987,536 (57.2) | 2,631 (56.7) | *-0.01* | 995,589 (57.6) | 2,135 (57.0) | *-0.01* | 1,002,326 (57.7) | 317 (57.1) | *-0.01* | 917,528 (56.7) | 715 (56.1) | *-0.01* |
| 16-Individuals receiving cash assistance or eligible under section 1931 -u children | 345 (0.0) | <11 | *0.00* | 440 (0.0) | <11 | *0.00* | <11 | <11 | *0.00* | <11 | <11 | *0.00* |
| 17-Individuals receiving cash assistance or eligible under section 1931 - u adults | 13,596 (0.8) | 34 (0.7) | *-0.01* | 14,807 (0.9) | 30 (0.8) | *-0.01* | 19,073 (1.1) | 5 (0.9) | *-0.02* | 11,484 (0.7) | <11 | *-0.01* |
| 22-Medically needy - blind/disabled | 4,439 (0.3) | 14 (0.3) | *0.01* | 4,633 (0.3) | 12 (0.3) | *0.01* | 2,665 (0.2) | <11 | *0.01* | 3,592 (0.2) | <11 | *0.00* |
| 24-Medically needy - children | 9,793 (0.6) | 29 (0.6) | *0.01* | 11,454 (0.7) | 27 (0.7) | *0.01* | 17,421 (1.0) | <11 | *0.01* | 5,321 (0.3) | <11 | *0.01* |
| 25-Medically needy - adults | 78,497 (4.5) | 216 (4.7) | *0.01* | 82,121 (4.8) | 182 (4.9) | *0.01* | 84,978 (4.9) | 28 (5.1) | *0.01* | 65,846 (4.1) | 52 (4.1) | *0.00* |
| 31-Poverty related eligibles - aged | <11 | <11 | *0.00* | <11 | <11 | *0.00* | <11 | <11 | *0.00* | <11 | <11 | *0.00* |
| 32-Poverty related eligibles - blind/disabled | 5,743 (0.3) | 16 (0.3) | *0.00* | 5,064 (0.3) | 11 (0.3) | *0.00* | 7,471 (0.4) | <11 | *0.02* | 6,241 (0.4) | <11 | *0.00* |
| 34-Poverty related eligibles - children | 37,733 (2.2) | 115 (2.5) | *0.02* | 39,060 (2.3) | 95 (2.5) | *0.02* | 43,802 (2.5) | 14 (2.5) | *0.00* | 31,560 (2.0) | 30 (2.4) | *0.03* |
| 35-Poverty related eligibles - adults | 134,176 (7.8) | 353 (7.6) | *-0.01* | 130,544 (7.6) | 278 (7.4) | *0.00* | 139,901 (8.1) | 44 (7.9) | *0.00* | 134,497 (8.3) | 104 (8.2) | *-0.01* |
| 3a-Poverty related eligibles - adults | 278 (0.0) | <11 | *0.00* | 349 (0.0) | <11 | *0.00* | <11 | <11 | *0.00* | <11 | <11 | *0.00* |
| 42-Other eligibles - blind/disabled | 12,880 (0.8) | 36 (0.8) | *0.00* | 12,839 (0.7) | 29 (0.8) | *0.00* | 8,907 (0.5) | <11 | *0.00* | 11,010 (0.7) | <11 | *0.00* |
| 44-Other eligibles - children | 10,070 (0.6) | 29 (0.6) | *0.01* | 11,314 (0.7) | 26 (0.7) | *0.00* | 14,749 (0.9) | <11 | *0.01* | 6,446 (0.4) | <11 | *0.01* |
| 45-Other eligibles - adults | 173,064 (10.0) | 449 (9.7) | *-0.01* | 175,511 (10.2) | 368 (9.8) | *-0.01* | 171,749 (9.9) | 54 (9.7) | *-0.01* | 160,385 (9.9) | 121 (9.5) | *-0.01* |
| 48-Other eligibles - foster care children | 3,510 (0.2) | <11 | *0.00* | 3,450 (0.2) | <11 | *0.00* | 3,218 (0.2) | <11 | *0.00* | 3,448 (0.2) | <11 | *0.00* |
| 52-Section 1115 demonstration Medicaid expansion | 692 (0.0) | <11 | *0.00* | 853 (0.1) | <11 | *0.00* | <11 | <11 | *0.00* | <11 | <11 | *0.00* |
| 54-Section 1115 demonstration Medicaid expansion | 0 (0.0) | <11 | *0.00* | 333 (0.0) | <11 | *0.00* | <11 | <11 | *0.00* | <11 | <11 | *0.00* |
| 55-Section 1115 demonstration Medicaid expansion | 73,689 (4.3) | 203 (4.4) | *0.01* | 71,071 (4.1) | 159 (4.3) | *0.01* | 53,642 (3.1) | 18 (3.2) | *0.01* | 66,125 (4.1) | 54 (4.2) | *0.01* |
| Multiple gestation, N (%) | 82,108 (4.8) | 219 (4.7) | *0.00* | 83,205 (4.8) | 177 (4.7) | *0.00* | 109,151 (6.3) | 34 (6.1) | *-0.01* | 75,768 (4.7) | 59 (4.6) | *0.00* |
| **Labeled indications, N (%)** |  |  |  |  |  |  |  |  |  |  |  |  |
| Epilepsy or seizures | 118,185 (6.8) | 347 (7.5) | *0.02* | 81,910 (4.7) | 193 (5.2) | *0.02* | 88,916 (5.1) | 36 (6.5) | *0.06* | 193,709 (12.0) | 179 (14.0) | *0.06* |
| Neuropathic pain | 360,011 (20.8) | 1,116 (24.0) | *0.08* | 363,242 (21.0) | 918 (24.5) | *0.08* | 180,551 (10.4) | 65 (11.7) | *0.04* | 305,300 (18.9) | 264 (20.7) | *0.05* |
| Restless legs syndrome | 9,769 (0.6) | 37 (0.8) | *0.03* | 7,961 (0.5) | 24 (0.6) | *0.02* | 2,767 (0.2) | <11 | *0.01* | 13,220 (0.8) | 15 (1.2) | *0.04* |
| **Pain conditions, N (%)** |  |  |  |  |  |  |  |  |  |  |  |  |
| Fibromyalgia | 130,801 (7.6) | 397 (8.6) | *0.04* | 132,918 (7.7) | 332 (8.9) | *0.04* | 92,486 (5.3) | 34 (6.1) | *0.03* | 105,938 (6.5) | 91 (7.1) | *0.02* |
| Arthritis, arthropathies and musculoskeletal pain | 687,430 (39.8) | 1,862 (40.1) | *0.01* | 693,126 (40.1) | 1,518 (40.5) | *0.01* | 524,663 (30.2) | 168 (30.3) | *0.00* | 612,018 (37.8) | 482 (37.8) | *0.00* |
| Back and neck pain | 778,936 (45.1) | 2,099 (45.2) | *0.00* | 773,032 (44.7) | 1,681 (44.9) | *0.00* | 505,163 (29.1) | 161 (29.0) | *0.00* | 728,913 (45.0) | 567 (44.5) | *-0.01* |
| Migraine or headache | 449,957 (26.0) | 1,194 (25.7) | *-0.01* | 456,281 (26.4) | 975 (26.0) | *-0.01* | 343,828 (19.8) | 110 (19.8) | *0.00* | 398,460 (24.6) | 313 (24.6) | *0.00* |
| Other pain conditions^2^ | 183,590 (10.6) | 573 (12.3) | *0.05* | 172,244 (10.0) | 435 (11.6) | *0.05* | 95,957 (5.5) | 34 (6.1) | *0.03* | 202,305 (12.5) | 182 (14.3) | *0.05* |
| **Psychiatric conditions, N (%)** |  |  |  |  |  |  |  |  |  |  |  |  |
| Depression | 486,116 (28.1) | 1,261 (27.2) | *-0.02* | 480,702 (27.8) | 1,013 (27.1) | *-0.02* | 305,217 (17.6) | 98 (17.7) | *0.00* | 438,959 (27.1) | 336 (26.4) | *-0.02* |
| Bipolar disorder | 204,516 (11.8) | 573 (12.3) | *0.02* | 191,232 (11.1) | 434 (11.6) | *0.02* | 117,222 (6.8) | 42 (7.6) | *0.03* | 207,098 (12.8) | 174 (13.7) | *0.03* |
| Anxiety | 393,323 (22.8) | 1,047 (22.6) | *0.00* | 370,371 (21.4) | 798 (21.3) | *0.00* | 261,725 (15.1) | 87 (15.7) | *0.02* | 422,354 (26.1) | 332 (26.0) | *0.00* |
| Attention deficit hyperactivity disorder | 54,731 (3.2) | 145 (3.1) | *0.00* | 49,283 (2.9) | 105 (2.8) | *0.00* | 51,263 (3.0) | 17 (3.1) | *0.01* | 64,948 (4.0) | 51 (4.0) | *0.00* |
| Psychosis | 37,281 (2.2) | 105 (2.3) | *0.01* | 36,325 (2.1) | 85 (2.3) | *0.01* | 11,763 (0.7) | <11 | *0.01* | 34,423 (2.1) | 29 (2.3) | *0.01* |
| Other psychiatric disorders | 126,233 (7.3) | 352 (7.6) | *0.01* | 128,514 (7.4) | 292 (7.8) | *0.01* | 69,544 (4.0) | 24 (4.3) | *0.02* | 95,422 (5.9) | 80 (6.3) | *0.02* |
| **Other maternal conditions, N (%)** |  |  |  |  |  |  |  |  |  |  |  |  |
| Hypertension | 152,404 (8.8) | 419 (9.0) | *0.01* | 144,835 (8.4) | 318 (8.5) | *0.00* | 124,243 (7.2) | 40 (7.2) | *0.00* | 160,078 (9.9) | 132 (10.4) | *0.02* |
| Diabetes | 135,470 (7.8) | 370 (8.0) | *0.00* | 134,915 (7.8) | 295 (7.9) | *0.00* | 89,150 (5.1) | 28 (5.1) | *0.00* | 127,049 (7.9) | 104 (8.2) | *0.01* |
| Renal disease | 27,350 (1.6) | 78 (1.7) | *0.01* | 24,155 (1.4) | 54 (1.4) | *0.00* | 12,771 (0.7) | <11 | *0.00* | 30,224 (1.9) | 25 (2.0) | *0.01* |
| Chronic fatigue syndrome | 152,454 (8.8) | 400 (8.6) | *-0.01* | 158,480 (9.2) | 336 (9.0) | *-0.01* | 108,545 (6.3) | 35 (6.3) | *0.00* | 109,188 (6.7) | 85 (6.7) | *0.00* |
| Nausea and vomiting | 364,720 (21.1) | 971 (20.9) | *0.00* | 389,777 (22.6) | 836 (22.3) | *-0.01* | 292,915 (16.9) | 93 (16.8) | *0.00* | 266,139 (16.4) | 211 (16.6) | *0.00* |
| Sleep disorder | 92,257 (5.3) | 257 (5.5) | *0.01* | 87,546 (5.1) | 197 (5.3) | *0.01* | 63,610 (3.7) | 21 (3.8) | *0.01* | 90,183 (5.6) | 74 (5.8) | *0.01* |
| **Lifestyle factors, N (%)** |  |  |  |  |  |  |  |  |  |  |  |  |
| Obesity or overweight | 102,598 (5.9) | 274 (5.9) | *0.00* | 99,402 (5.8) | 213 (5.7) | *0.00* | 92,442 (5.3) | 29 (5.2) | *0.00* | 94,696 (5.9) | 75 (5.9) | *0.00* |
| Smoking | 266,069 (15.4) | 729 (15.7) | *0.01* | 253,690 (14.7) | 557 (14.9) | *0.01* | 187,742 (10.8) | 60 (10.8) | *0.00* | 282,640 (17.5) | 225 (17.7) | *0.00* |
| Drug abuse or dependence | 88,302 (5.1) | 245 (5.3) | *0.01* | 88,288 (5.1) | 201 (5.4) | *0.01* | 56,119 (3.2) | 20 (3.6) | *0.02* | 68,967 (4.3) | 56 (4.4) | *0.01* |
| Alcohol abuse or dependence | 39,439 (2.3) | 107 (2.3) | *0.00* | 40,123 (2.3) | 89 (2.4) | *0.00* | 6,164 (0.4) | <11 | *0.00* | 30,485 (1.9) | 24 (1.9) | *0.00* |
| **Concomitant use of medications, N (%)** |  |  |  |  |  |  |  |  |  |  |  |  |
| Anticonvulsants prior to pregnancy |  |  |  |  |  |  |  |  |  |  |  |  |
| Carbamazepine | 27,838 (1.6) | 78 (1.7) | *0.01* | 14,813 (0.9) | 32 (0.9) | *0.00* | 11,045 (0.6) | <11 | *0.01* | 55,422 (3.4) | 52 (4.1) | *0.03* |
| Phenytoin | 22,790 (1.3) | 69 (1.5) | *0.01* | 15,342 (0.9) | 37 (1.0) | *0.01* | 16,502 (1.0) | <11 | *0.03* | 36,532 (2.3) | 34 (2.7) | *0.03* |
| Topiramate | 56,916 (3.3) | 165 (3.6) | *0.01* | 53,321 (3.1) | 127 (3.4) | *0.02* | 18,006 (1.0) | <11 | *0.00* | 53,226 (3.3) | 45 (3.5) | *0.01* |
| Valproate | 45,900 (2.7) | 119 (2.6) | *-0.01* | 41,404 (2.4) | 87 (2.3) | *0.00* | 22,552 (1.3) | <11 | *0.01* | 52,053 (3.2) | 42 (3.3) | *0.00* |
| Other anticonvulsants | 108,275 (6.3) | 304 (6.6) | *0.01* | 90,763 (5.3) | 208 (5.6) | *0.01* | 79,947 (4.6) | 30 (5.4) | *0.04* | 141,031 (8.7) | 120 (9.4) | *0.02* |
| Anticonvulsants in first trimester |  |  |  |  |  |  |  |  |  |  |  |  |
| Carbamazepine | 25,323 (1.5) | 74 (1.6) | *0.01* | 12,829 (0.7) | 29 (0.8) | *0.00* | 5,669 (0.3) | <11 | *0.01* | 52,126 (3.2) | 51 (4.0) | *0.04* |
| Phenytoin | 18,494 (1.1) | 55 (1.2) | *0.01* | 12,922 (0.8) | 31 (0.8) | *0.01* | 12,532 (0.7) | <11 | *0.02* | 28,387 (1.8) | 26 (2.0) | *0.02* |
| Topiramate | 50,776 (2.9) | 158 (3.4) | *0.03* | 47,382 (2.7) | 122 (3.3) | *0.03* | 12,470 (0.7) | <11 | *0.00* | 53,291 (3.3) | 47 (3.7) | *0.02* |
| Valproate | 32,108 (1.9) | 87 (1.9) | *0.00* | 24,400 (1.4) | 53 (1.4) | *0.00* | 18,701 (1.1) | <11 | *0.02* | 45,690 (2.8) | 39 (3.1) | *0.01* |
| Other anticonvulsants | 98,344 (5.7) | 288 (6.2) | *0.02* | 84,668 (4.9) | 205 (5.5) | *0.03* | 53,961 (3.1) | 20 (3.6) | *0.03* | 119,066 (7.4) | 104 (8.2) | *0.03* |
| Opioids and opioid-related treatment prior to pregnancy |  |  |  |  |  |  |  |  |  |  |  |  |
| Codeine | 134,487 (7.8) | 348 (7.5) | *-0.01* | 134,843 (7.8) | 280 (7.5) | *-0.01* | 127,866 (7.4) | 39 (7.0) | *-0.01* | 127,983 (7.9) | 96 (7.5) | *-0.01* |
| Hydrocodone | 623,670 (36.1) | 1,644 (35.4) | *-0.01* | 623,226 (36.1) | 1,329 (35.5) | *-0.01* | 586,649 (33.8) | 180 (32.4) | *-0.03* | 605,087 (37.4) | 458 (35.9) | *-0.03* |
| Oxycodone | 254,113 (14.7) | 738 (15.9) | *0.03* | 246,982 (14.3) | 585 (15.6) | *0.04* | 186,602 (10.7) | 62 (11.2) | *0.01* | 265,559 (16.4) | 217 (17.0) | *0.02* |
| Tramadol | 260,712 (15.1) | 715 (15.4) | *0.01* | 243,573 (14.1) | 537 (14.3) | *0.01* | 169,561 (9.8) | 55 (9.9) | *0.00* | 280,181 (17.3) | 221 (17.3) | *0.00* |
| Other opioids | 69,724 (4.0) | 230 (5.0) | *0.04* | 64,666 (3.7) | 174 (4.7) | *0.05* | 55,466 (3.2) | 22 (4.0) | *0.04* | 76,676 (4.7) | 72 (5.7) | *0.04* |
| Buprenorphine | 39,304 (2.3) | 112 (2.4) | *0.01* | 31,443 (1.8) | 72 (1.9) | *0.01* | 19,340 (1.1) | <11 | *0.00* | 56,634 (3.5) | 46 (3.6) | *0.01* |
| Opioid antagonists | 39,754 (2.3) | 113 (2.4) | *0.01* | 31,684 (1.8) | 72 (1.9) | *0.01* | 17,433 (1.0) | <11 | *0.01* | 55,357 (3.4) | 45 (3.5) | *0.01* |
| Morphine equivalents, mg, mean (SD) | 830.9 (2,008.1) | 960.6 (2,200.4) | *0.06* | 778.5 (1,942.2) | 905.5 (2,138.7) | *0.06* | 543.0 (1,540.5) | 591.2 (1,647.2) | *0.03* | 1,053.2 (2,279.0) | 1,153.7 (2,426.9) | *0.04* |
| Opioids and opioid-related treatment in first trimester |  |  |  |  |  |  |  |  |  |  |  |  |
| Codeine | 164,449 (9.5) | 418 (9.0) | *-0.02* | 170,188 (9.9) | 351 (9.4) | *-0.02* | 149,739 (8.6) | 44 (7.9) | *-0.03* | 137,931 (8.5) | 100 (7.8) | *-0.02* |
| Hydrocodone | 567,426 (32.8) | 1,505 (32.4) | *-0.01* | 558,337 (32.3) | 1,195 (31.9) | *-0.01* | 514,850 (29.6) | 158 (28.5) | *-0.03* | 576,777 (35.6) | 442 (34.7) | *-0.02* |
| Oxycodone | 215,090 (12.5) | 642 (13.8) | *0.04* | 208,428 (12.1) | 503 (13.4) | *0.04* | 175,417 (10.1) | 59 (10.6) | *0.02* | 239,389 (14.8) | 202 (15.8) | *0.03* |
| Tramadol | 207,664 (12.0) | 591 (12.7) | *0.02* | 200,788 (11.6) | 463 (12.4) | *0.02* | 160,327 (9.2) | 53 (9.6) | *0.01* | 213,324 (13.2) | 170 (13.3) | *0.00* |
| Other opioids | 67,474 (3.9) | 230 (5.0) | *0.05* | 61,358 (3.6) | 171 (4.6) | *0.05* | 50,416 (2.9) | 20 (3.6) | *0.04* | 86,330 (5.3) | 84 (6.6) | *0.05* |
| Buprenorphine | 46,634 (2.7) | 140 (3.0) | *0.02* | 37,553 (2.2) | 89 (2.4) | *0.01* | 20,038 (1.2) | <11 | *-0.01* | 66,291 (4.1) | 57 (4.5) | *0.02* |
| Opioid antagonists^3^ | 39,278 (2.3) | 116 (2.5) | *0.01* | 31,430 (1.8) | 73 (2.0) | *0.01* | 14,822 (0.9) | <11 | *0.01* | 55,154 (3.4) | 47 (3.7) | *0.02* |
| Morphine equivalents, mg, mean (SD) | 920.6 (2,479.0) | 1,064.4 (2,662.2) | *0.06* | 843.6 (2,343.5) | 978.3 (2,532.3) | *0.06* | 662.4 (2,086.8) | 733.3 (2,228.4) | *0.03* | 1,248.9 (2,978.8) | 1,395.5 (3,127.2) | *0.05* |
| Other medications |  |  |  |  |  |  |  |  |  |  |  |  |
| Acetaminophen | 940,495 (54.4) | 2,441 (52.6) | *-0.04* | 945,438 (54.7) | 1,974 (52.7) | *-0.04* | 883,316 (50.9) | 268 (48.3) | *-0.05* | 902,899 (55.8) | 684 (53.7) | *-0.04* |
| NSAIDs | 786,336 (45.5) | 2,051 (44.2) | *-0.03* | 817,966 (47.3) | 1,717 (45.9) | *-0.03* | 712,162 (41.0) | 219 (39.5) | *-0.03* | 666,998 (41.2) | 513 (40.2) | *-0.02* |
| Triptans | 127,239 (7.4) | 349 (7.5) | *0.01* | 125,863 (7.3) | 282 (7.5) | *0.01* | 76,867 (4.4) | 25 (4.5) | *0.00* | 119,382 (7.4) | 94 (7.4) | *0.00* |
| Antidepressants | 1,024,007 (59.3) | 2,595 (55.9) | *-0.07* | 992,094 (57.4) | 2,036 (54.4) | *-0.06* | 579,910 (33.4) | 183 (33.0) | *-0.01* | 956,864 (59.1) | 714 (56.0) | *-0.06* |
| Benzodiazepines | 472,952 (27.4) | 1,275 (27.5) | *0.00* | 447,859 (25.9) | 981 (26.2) | *0.01* | 367,221 (21.1) | 123 (22.2) | *0.02* | 505,873 (31.3) | 399 (31.3) | *0.00* |
| Other anxiolytics | 72,245 (4.2) | 203 (4.4) | *0.01* | 69,567 (4.0) | 158 (4.2) | *0.01* | 44,589 (2.6) | 16 (2.9) | *0.02* | 66,751 (4.1) | 53 (4.2) | *0.00* |
| Other hypnotics | 326,639 (18.9) | 904 (19.5) | *0.01* | 315,432 (18.3) | 707 (18.9) | *0.02* | 208,224 (12.0) | 69 (12.4) | *0.01* | 331,343 (20.5) | 269 (21.1) | *0.02* |
| Barbiturates | 100,861 (5.8) | 271 (5.8) | *0.00* | 96,900 (5.6) | 210 (5.6) | *0.00* | 86,710 (5.0) | 29 (5.2) | *0.01* | 110,737 (6.8) | 89 (7.0) | *0.01* |
| Antipsychotics | 273,962 (15.9) | 761 (16.4) | *0.01* | 264,450 (15.3) | 603 (16.1) | *0.02* | 157,987 (9.1) | 58 (10.5) | *0.05* | 246,349 (15.2) | 203 (15.9) | *0.02* |
| Stimulants | 109,573 (6.3) | 310 (6.7) | *0.01* | 92,538 (5.4) | 209 (5.6) | *0.01* | 85,444 (4.9) | 30 (5.4) | *0.02* | 139,622 (8.6) | 117 (9.2) | *0.02* |
| Antihypertensive medications | 264,270 (15.3) | 739 (15.9) | *0.02* | 248,935 (14.4) | 555 (14.8) | *0.01* | 177,386 (10.2) | 58 (10.5) | *0.01* | 275,410 (17.0) | 229 (18.0) | *0.02* |
| Non-insulin antidiabetic medications | 80,295 (4.7) | 215 (4.6) | *0.00* | 78,739 (4.6) | 171 (4.6) | *0.00* | 43,194 (2.5) | 13 (2.3) | *-0.01* | 73,791 (4.6) | 58 (4.6) | *0.00* |
| Insulin | 89,787 (5.2) | 246 (5.3) | *0.00* | 84,870 (4.9) | 184 (4.9) | *0.00* | 54,052 (3.1) | 17 (3.1) | *0.00* | 95,188 (5.9) | 78 (6.1) | *0.01* |
| Corticosteroids | 519,312 (30.1) | 1,373 (29.6) | *-0.01* | 527,264 (30.5) | 1,124 (30.0) | *-0.01* | 424,569 (24.5) | 133 (24.0) | *-0.01* | 446,114 (27.6) | 349 (27.4) | *0.00* |
| Fluconazole | 165,166 (9.6) | 434 (9.4) | *-0.01* | 169,585 (9.8) | 359 (9.6) | *-0.01* | 144,308 (8.3) | 45 (8.1) | *-0.01* | 149,567 (9.2) | 116 (9.1) | *0.00* |
| Other teratogenic agents^4^ | 69,559 (4.0) | 182 (3.9) | *-0.01* | 71,726 (4.2) | 153 (4.1) | *0.00* | 70,212 (4.0) | 22 (4.0) | *0.00* | 62,388 (3.9) | 48 (3.8) | *0.00* |
| **Markers of burden of disease** |  |  |  |  |  |  |  |  |  |  |  |  |
| Obstetric Comorbidity Index^5^, mean (SD) | 1.7 (1.9) | 1.7 (2.0) | *0.01* | 1.6 (1.9) | 1.6 (1.9) | *0.01* | 1.6 (1.8) | 1.6 (1.8) | *-0.01* | 1.9 (2.0) | 1.9 (2.1) | *0.02* |
| Number of distinct filled prescriptions, mean (SD) | 6.1 (4.1) | 6.1 (4.4) | *-0.02* | 6.0 (4.1) | 5.9 (4.3) | *-0.03* | 4.5 (4.0) | 4.4 (4.1) | *-0.02* | 6.3 (4.2) | 6.3 (4.5) | *0.00* |
| Number of diagnoses, mean (SD) | 6.8 (5.3) | 6.6 (5.3) | *-0.03* | 6.8 (5.2) | 6.6 (5.2) | *-0.03* | 5.3 (4.9) | 5.4 (5.2) | *0.01* | 6.7 (5.3) | 6.6 (5.5) | *-0.03* |
| Number of outpatient physician visits, mean (SD) | 5.9 (6.5) | 6.2 (7.4) | *0.04* | 5.8 (6.3) | 6.1 (7.5) | *0.04* | 4.4 (5.6) | 4.7 (6.9) | *0.05* | 5.9 (6.6) | 6.0 (6.7) | *0.01* |
| Patients hospitalized, N (%) | 106,281 (6.2) | 305 (6.6) | *0.02* | 101,838 (5.9) | 235 (6.3) | *0.02* | 97,117 (5.6) | 33 (6.0) | *0.02* | 106,779 (6.6) | 91 (7.1) | *0.02* |
| Number of hospitalizations, mean (SD) | 0.1 (0.3) | 0.1 (0.3) | *0.02* | 0.1 (0.3) | 0.1 (0.3) | *0.02* | 0.1 (0.3) | 0.1 (0.3) | *0.02* | 0.1 (0.3) | 0.1 (0.3) | *0.02* |
| Number of days hospitalized, mean (SD) | 0.3 (2.0) | 0.4 (2.2) | *0.03* | 0.3 (2.0) | 0.4 (2.3) | *0.03* | 0.3 (1.8) | 0.3 (1.6) | *0.01* | 0.4 (2.1) | 0.4 (1.8) | *0.02* |
| Number of emergency room visits, mean (SD) | 1.0 (1.9) | 0.9 (1.7) | *-0.02* | 1.0 (1.9) | 0.9 (1.7) | *-0.02* | 0.8 (1.7) | 0.8 (1.5) | *-0.02* | 1.0 (1.9) | 0.9 (1.6) | *-0.02* |
| T1: first trimester; St. Diff.: Standardized differences, i.e., the difference in means or proportions divided by the pooled standard deviation [Austin PC. Balance diagnostics for comparing the distribution of baseline covariates between treatment groups in propensity-score matched samples. Statistics in medicine 2009;28:3083-107]; SD: standard deviation  Maternal comorbidities and concomitant medication use were measured during the 3 months before pregnancy through the end of the first trimester. Measures of healthcare intensity (e.g., number of medical visits) were measured only during the 3-month period before pregnancy, in order for these not to be affected by early awareness of possible pregnancy complications. ^1^Other race includes Asian, Native American, Other, and Unknown ^2^ Other pain conditions include osteoarthritis, rheumatoid arthritis, generalized pain, pain disorders related to psychological factors, and pain not elsewhere classified ^3^ Opioid antagonists include naloxone and naltrexone ^4^ Other teratogenic agents include methimazole, danazol, propylthiouracil, and progestins ^5^ The obstetric comorbidity index predicts severe maternal morbidity. The range for the maternal comorbidity index is 0 to 45, with lower values associated with lower burden of maternal illness and higher values associated with higher burden of maternal illness [Bateman BT, Mhyre JM, Hernandez-Diaz S, Huybrechts KF, Fischer MA, Creanga AA, Callaghan WM, Gagne JJ. Development of a comorbidity index for use in obstetric patients. Obstet Gynecol. 2013;122:957-65] In accordance with the data use agreement, we do not report information for frequency cells with less than 11 cases. These are noted as <11 | | | | | | | | | | | | |
