# Supplementary material for: Gabapentin in pregnancy and the risk of adverse neonatal and maternal outcomes: A population-based cohort study nested in the US Medicaid Analytic eXtract dataset
Source: PLoS Med. 2020 Sep 1;17(9):e1003322. doi: 10.1371/journal.pmed.1003322 (PMC7462308; doi:10.1371/journal.pmed.1003322)
Supplement: S5 Table — PS, propensity score; RR, relative risk. (DOCX) [file pmed.1003322.s005.docx]

# S5 Table. Sensitivity and secondary analyses for the relative risk of neonatal and maternal outcomes associated with exposure to gabapentin compared with unexposed pregnancies before and after PS adjustment

| **Exposure Group:** | **Unexposed** | **Exposed**  **during T1** |  | **Exposed  early in pregnancy** |  | | **Exposed  late in pregnancy** | |  | | **Exposed  early and late in pregnancy** | |  | | |
| --- | --- | --- | --- | --- | --- | --- | --- | --- | --- | --- | --- | --- | --- | --- | --- |
| **Outcomes** |  | **PS-adjusted  RR (95% CI)** | **p-value** | **PS-adjusted  RR (95% CI)** | **p-value** | **PS-adjusted  RR (95% CI)** | | **p-value** | | **PS-adjusted  RR (95% CI)** | | **p-value** | | |  |
| **Major congenital malformations** |  |  |  |  |  |  | |  | |  | |  | | |  |
| Main unadjusted analysis | Ref. | 1.49 (1.31-1.69) | <0.001 | . |  | . | |  | | . | |  | | |  |
| Main PS-adjusted analysis | **Ref.** | **1.07 (0.94-1.21)** | **0.33** | . |  | . | |  | | . | |  | | |  |
| ≥ 2 Rx, unadjusted | Ref. | 1.65 (1.37-1.99) | <0.001 | . |  | . | |  | | . | |  | | |  |
| ≥ 2 Rx, PS-adjusted | Ref. | 1.15 (0.95-1.39) | 0.15 | . |  | . | |  | | . | |  | | |  |
| Infant claims, unadjusted | Ref. | 1.52 (1.33-1.73) | <0.001 | . |  | . | |  | | . | |  | | |  |
| Infant claims, PS-adjusted | Ref. | 1.05 (0.92-1.20) | 0.48 | . |  | . | |  | | . | |  | | |  |
| 1-year follow-up^1^, unadjusted | Ref. | 1.51 (1.34-1.69) | <0.001 | . |  | . | |  | | . | |  | | |  |
| 1-year follow-up^1^, PS-adjusted | Ref. | 1.05 (0.94-1.18) | 0.37 | . |  | . | |  | | . | |  | | |  |
| High-dimensional-PS-adjusted | Ref. | 1.06 (0.93-1.20) | 0.40 | . |  | . | |  | | . | |  | | |  |
| ≥ 1 epilepsy or seizure dx, unadjusted | Ref. | 1.22 (0.81-1.84) | 0.34 | . |  | . | |  | | . | |  | | |  |
| ≥ 1 epilepsy or seizure dx, PS-adjusted | Ref. | 1.01 (0.66-1.56) | 0.96 | . |  | . | |  | | . | |  | | |  |
| No epilepsy or seizure dx, unadjusted | Ref. | 1.46 (1.28-1.67) | <0.001 | . |  | . | |  | | . | |  | | |  |
| No epilepsy or seizure dx, PS-adjusted | Ref. | 1.07 (0.93-1.22) | 0.35 | . |  | . | |  | | . | |  | | |  |
| ≥ 1 pain dx^2^, unadjusted | Ref. | 1.33 (1.15-1.53) | <0.001 | . |  | . | |  | | . | |  | | |  |
| ≥ 1 pain dx^2^, PS-adjusted | Ref. | 1.06 (0.91-1.22) | 0.48 | . |  | . | |  | | . | |  | | |  |
| No pain dx, unadjusted | Ref. | 1.50 (1.16-1.96) | <0.01 | . |  | . | |  | | . | |  | | |  |
| No pain dx, PS-adjusted | Ref. | 1.14 (0.87-1.49) | 0.33 | . |  | . | |  | | . | |  | | |  |
| **Cardiac malformations** |  |  |  |  |  |  | |  | |  | |  | | |  |
| Main unadjusted analysis | Ref. | 1.77 (1.43-2.18) | <0.001 | . |  | . | |  | | . | |  | | |  |
| Main PS-adjusted analysis | **Ref.** | **1.12 (0.89-1.40)** | **0.35** | . |  | . | |  | | . | |  | | |  |
| ≥ 2 Rx, unadjusted | Ref. | 2.44 (1.85-3.22) | <0.001 | . |  | . | |  | | . | |  | | |  |
| ≥ 2 Rx, PS-adjusted | Ref. | 1.40 (1.03-1.90) | 0.03 | . |  | . | |  | | . | |  | | |  |
| Infant claims, unadjusted | Ref. | 1.88 (1.52-2.34) | <0.001 | . |  | . | |  | | . | |  | | |  |
| Infant claims, PS-adjusted | Ref. | 1.13 (0.89-1.44) | 0.33 | . |  | . | |  | | . | |  | | |  |
| 1-year follow-up^1^, unadjusted | Ref. | 1.70 (1.36-2.12) | <0.001 | . |  | . | |  | | . | |  | | |  |
| 1-year follow-up^1^, PS-adjusted | Ref. | 1.10 (0.92-1.53) | 0.41 | . |  | . | |  | | . | |  | | |  |
| High-dimensional-PS-adjusted | Ref. | 1.11 (0.88-1.40) | 0.37 | . |  | . | |  | | . | |  | | |  |
| High-dimensional-PS-adjusted, ≥2 Rx^3^ | Ref. | 1.40 (1.03-1.90) | 0.03 | . |  | . | |  | | . | |  | | |  |
| ≥ 1 epilepsy or seizure dx, unadjusted | Ref. | 1.85 (1.02-3.37) | 0.04 | . |  | . | |  | | . | |  | | |  |
| ≥ 1 epilepsy or seizure dx, PS-adjusted | Ref. | 1.40 (0.73-2.71) | 0.31 | . |  | . | |  | | . | |  | | |  |
| No epilepsy or seizure dx, unadjusted | Ref. | 1.67 (1.34-2.09) | <0.001 | . |  | . | |  | | . | |  | | |  |
| No epilepsy or seizure dx, PS-adjusted | Ref. | 1.08 (0.84-1.38) | 0.56 | . |  | . | |  | | . | |  | | |  |
| ≥ 1 pain dx^2^, unadjusted | Ref. | 1.50 (1.18-4.12) | <0.01 | . |  | . | |  | | . | |  | | |  |
| ≥ 1 pain dx^2^, PS-adjusted | Ref. | 1.07 (0.82-1.40) | 0.61 | . |  | . | |  | | . | |  | | |  |
| No pain dx, unadjusted | Ref. | 2.05 (1.37-3.07) | <0.001 | . |  | . | |  | | . | |  | | |  |
| No pain dx, PS-adjusted | Ref. | 1.32 (0.84-2.06) | 0.23 | . |  | . | |  | | . | |  | | |  |
| **Preeclampsia** |  |  |  |  |  |  | |  | |  | |  | | |  |
| Main unadjusted analysis | Ref. |  |  | 1.17 (1.01-1.35) | 0.03 | 1.43 (1.03-2.00) | | 0.03 | | 1.52 (1.23-1.87) | | <0.001 | | |  |
| Main PS-adjusted analysis | **Ref.** | . |  | **0.87 (0.75-1.00)** | **0.05** | **0.96 (0.69-1.33)** | | **0.80** | | **0.92 (0.74-1.13)** | | **0.42** | | |  |
| ≥ 2 Rx, unadjusted | Ref. | . |  | 1.45 (1.16-1.82) | <0.01 | 1.54 (0.85-2.80) | | 0.16 | | 1.46 (1.11-1.90) | | <0.01 | | |  |
| ≥ 2 Rx, PS-adjusted | Ref. | . |  | 0.98 (0.78-1.23) | 0.88 | 1.17 (0.64-2.14) | | 0.60 | | 0.87 (0.67-1.14) | | 0.32 | | |  |
| High-dimensional-PS-adjusted | Ref. | . |  | 0.86 (0.75-1.00) | 0.05 | 0.96 (0.69-1.33) | | 0.78 | | 1.00 (0.80-1.25) | | 0.99 | | |  |
| Updated CAP^4^, unadjusted | Ref. | . |  | 1.17 (1.02-1.35) | 0.03 | 1.43 (1.03-2.00) | | 0.03 | | 1.52 (1.23-1.87) | | <0.001 | | |  |
| Updated CAP^4^, PS-adjusted | Ref. | . |  | 0.88 (0.76-1.02) | 0.08 | 0.98 (0.70-1.36) | | 0.89 | | 0.93 (0.75-1.15) | | 0.51 | | |  |
| ≥ 1 epilepsy or seizure dx, unadjusted | Ref. | . |  | 0.88 (0.46-1.68) | 0.71 | 1.05 (0.27-4.06) | | 0.94 | | 1.16 (0.65-2.07) | | 0.61 | | |  |
| ≥ 1 epilepsy or seizure dx, PS-adjusted | Ref. | . |  | 0.74 (0.39-1.41) | 0.37 | 1.43 (0.37-5.46) | | 0.60 | | 0.82 (0.46-1.46) | | 0.49 | | |  |
| No epilepsy or seizure dx, unadjusted | Ref. | . |  | 1.18 (1.02-1.36) | 0.03 | 1.44 (1.03-2.03) | | 0.04 | | 1.52 (1.21-1.92) | | <0.001 | | |  |
| No epilepsy or seizure dx, PS-adjusted | Ref. | . |  | 0.88 (0.76-1.01) | 0.08 | 0.96 (0.68-1.35) | | 0.82 | | 0.96 (0.76-1.20) | | 0.71 | | |  |
| ≥ 1 pain dx^2^, unadjusted | Ref. | . |  | 1.11 (0.95-1.30) | 0.20 | 1.13 (0.71-1.80) | | 0.60 | | 1.44 (1.14-1.83) | | <0.01 | | |  |
| ≥ 1 pain dx^2^, PS-adjusted | Ref. | . |  | 0.91 (0.77-1.06) | 0.23 | 0.83 (0.52-1.31) | | 0.42 | | 0.94 (0.74-1.19) | | 0.61 | | |  |
| No pain dx, unadjusted | Ref. | . |  | 1.00 (0.73-1.37) | 0.99 | 1.72 (1.07-2.76) | | 0.03 | | 1.24 (0.76-2.03) | | 0.40 | | |  |
| No pain dx, PS-adjusted | Ref. | . |  | 0.74 (0.54-1.02) | 0.06 | 1.18 (0.74-1.89) | | 0.49 | | 0.84 (0.51-1.38) | | 0.49 | | |  |
| **Preterm delivery** |  |  |  |  |  |  | |  | |  | |  | | |  |
| Main unadjusted analysis | Ref. | . |  | 1.46 (1.35-1.57) | <0.001 | 1.84 (1.55-2.18) | | <0.001 | | 1.93 (1.73-2.16) | | <0.001 | | |  |
| Main PS-adjusted analysis | **Ref.** | . |  | **1.00 (0.93-1.08)** | **0.89** | **1.28 (1.08-1.52)** | | **<0.01** | | **1.22 (1.09-1.36)** | | **<0.001** | | |  |
| ≥ 2 Rx, unadjusted | Ref. | . |  | 1.56 (1.37-1.77) | <0.001 | 1.89 (1.38-2.59) | | <0.01 | | 2.02 (1.77-2.30) | | <0.001 | | |  |
| ≥ 2 Rx, PS-adjusted | Ref. | . |  | 0.98 (0.86-1.12) | 0.81 | 1.27 (0.93-1.74) | | 0.14 | | 1.28 (1.12-1.46) | | <0.001 | | |  |
| High-dimensional-PS-adjusted | Ref. | . |  | 1.01 (0.93-1.08) | 0.89 | 1.25 (1.06-1.49) | | <0.01 | | 1.22 (1.08-1.37) | | <0.01 | | |  |
| Updated CAP^4^, unadjusted | Ref. | . |  | 1.46 (1.35-1.57) | <0.001 | 1.84 (1.55-2.18) | | <0.001 | | 1.93 (1.73-2.16) | | <0.001 | | |  |
| Updated CAP^4^, PS-adjusted | Ref. | . |  | 0.99 (0.92-1.06) | 0.74 | 1.24 (1.04-1.47) | | 0.01 | | 1.20 (1.08-1.34) | | <0.01 | | |  |
| ≥ 1 epilepsy or seizure dx, unadjusted | Ref. | . |  | 1.31 (0.99-1.74) | 0.06 | 1.41 (0.76-2.60) | | 0.27 | | 1.56 (1.20-2.02) | | <0.001 | | |  |
| ≥ 1 epilepsy or seizure dx, PS-adjusted | Ref. | . |  | 1.07 (0.81-1.42) | 0.62 | 1.14 (0.51-2.51) | | 0.75 | | 1.47 (1.13-1.91) | | <0.01 | | |  |
| No epilepsy or seizure dx, unadjusted | Ref. | . |  | 1.43 (1.33-1.55) | <0.001 | 1.83 (1.53-2.84) | | <0.001 | | 1.87 (1.66-2.11) | | <0.001 | | |  |
| No epilepsy or seizure dx, PS-adjusted | Ref. | . |  | 1.00 (0.93-1.08) | 0.94 | 1.30 (1.09-1.55) | | <0.01 | | 1.20 (1.06-1.35) | | <0.01 | | |  |
| ≥ 1 pain dx^2^, unadjusted | Ref. | . |  | 1.27 (1.16-1.37) | <0.001 | 1.71 (1.38-2.12) | | <0.001 | | 1.80 (1.60-2.03) | | <0.001 | | |  |
| ≥ 1 pain dx^2^, PS-adjusted | Ref. | . |  | 0.96 (0.88-1.04) | 0.32 | 1.24 (1.00-1.54) | | 0.05 | | 1.22 (1.08-1.38) | | <0.01 | | |  |
| No pain dx, unadjusted | Ref. | . |  | 1.54 (1.33-1.79) | <0.001 | 1.75 (1.33-2.31) | | <0.001 | | 1.61 (1.24-2.08) | | <0.001 | | |  |
| No pain dx, PS-adjusted | Ref. | . |  | 1.15 (0.99-1.34) | 0.07 | 1.33 (1.01-1.75) | | 0.05 | | 1.19 (0.92-1.54) | | 0.17 | | |  |
| **Small for gestational age (SGA)** |  |  |  |  |  |  | |  | |  | |  | | |  |
| Main unadjusted analysis | Ref. | . |  | 1.71 (1.50-1.96) | <0.001 | 1.97 (1.43-2.71) | | <0.001 | | 2.26 (1.85-2.75) | | <0.001 | | |  |
| Main PS-adjusted analysis | **Ref.** | . |  | **1.17 (1.02-1.33)** | **0.02** | **1.39 (1.01-1.91)** | | **0.05** | | **1.32 (1.08-1.60)** | | **<0.01** | | |  |
| ≥ 2 Rx, unadjusted | Ref. | . |  | 1.88 (1.50-2.36) | <0.001 | 1.99 (1.09-3.63) | | 0.02 | | 2.22 (1.74-2.84) | | <0.001 | | |  |
| ≥ 2 Rx, PS-adjusted | Ref. | . |  | 1.15 (0.92-1.45) | 0.22 | 1.35 (0.74-2.46) | | 0.33 | | 1.28 (1.00-1.63) | | 0.05 | | |  |
| High-dimensional-PS-adjusted | Ref. | . |  | 1.17 (1.02-1.33) | 0.02 | 1.36 (0.99-1.88) | | 0.06 | | 1.39 (1.12-1.72) | | <0.01 | | |  |
| Updated CAP^4^, unadjusted | Ref. | . |  | 1.71 (1.50-1.96) | <0.001 | 1.97 (1.43-2.71) | | <0.001 | | 2.26 (1.85-2.75) | | <0.001 | | |  |
| Updated CAP^4^, PS-adjusted | Ref. | . |  | 1.15 (1.01-1.32) | 0.04 | 1.36 (0.99-1.88) | | 0.06 | | 1.31 (1.08-1.60) | | <0.01 | | |  |
| ≥ 1 epilepsy or seizure dx, unadjusted | Ref. | . |  | 1.53 (0.93-2.50) | 0.09 | 0.55 (0.08-3.78) | | 0.54 | | 1.65 (1.01-2.69) | | 0.05 | | |  |
| ≥ 1 epilepsy or seizure dx, PS-adjusted | Ref. | . |  | 1.35 (0.83-2.20) | 0.23 | 1.03 (0.15-7.10) | | 0.98 | | 1.46 (0.89-2.38) | | 0.13 | | |  |
| No epilepsy or seizure dx, unadjusted | Ref. | . |  | 1.68 (1.46-1.93) | <0.001 | 2.05 (1.48-2.84) | | <0.001 | | 2.21 (1.78-2.74) | | <0.001 | | |  |
| No epilepsy or seizure dx, PS-adjusted | Ref. | . |  | 1.17 (1.01-1.34) | 0.03 | 1.46 (1.05-2.02) | | 0.02 | | 1.30 (1.05-1.62) | | 0.02 | | |  |
| ≥ 1 pain dx^2^, unadjusted | Ref. | . |  | 1.56 (1.34-1.81) | <0.001 | 1.77 (1.17-2.68) | | <0.01 | | 2.08 (1.67-2.59) | | <0.001 | | |  |
| ≥ 1 pain dx^2^, PS-adjusted | Ref. | . |  | 1.16 (1.00-1.35) | 0.05 | 1.33 (0.88-2.01) | | 0.18 | | 1.29 (1.04-1.61) | | 0.02 | | |  |
| No pain dx, unadjusted | Ref. | . |  | 1.54 (1.16-2.06) | <0.01 | 1.96 (1.18-3.25) | | <0.01 | | 1.93 (1.24-3.03) | | <0.01 | | |  |
| No pain dx, PS-adjusted | Ref. | . |  | 1.14 (0.85-1.51) | 0.39 | 1.44 (0.86-2.39) | | 0.16 | | 1.32 (0.84-2.07) | | 0.22 | | |  |
| **NICU admission** |  |  |  |  |  |  | |  | |  | |  | | |  |
| Main unadjusted analysis | Ref. | . |  | 1.89 (1.73-2.07) | <0.001 | 2.11 (1.69-2.63) | | <0.001 | | 3.03 (2.69-3.41) | | <0.001 | | |  |
| Main PS-adjusted analysis | **Ref.** | . |  | **1.01 (0.93-1.11)** | **0.77** | **1.21 (0.97-1.51)** | | **0.09** | | **1.35 (1.20-1.52)** | | **<0.001** | | |  |
| ≥ 2 Rx, unadjusted | Ref. | . |  | 2.31 (1.99-2.67) | <0.001 | 1.87 (1.19-2.92) | | <0.01 | | 3.22 (2.79-3.71) | | <0.001 | | |  |
| ≥ 2 Rx, PS-adjusted | Ref. | . |  | 1.08 (0.93-1.25) | 0.31 | 1.00 (0.64-1.57) | | 0.99 | | 1.43 (1.24-1.65) | | <0.001 | | |  |
| High-dimensional-PS-adjusted | Ref. | . |  | 1.01 (0.92-1.11) | 0.80 | 1.19 (0.95-1.49) | | 0.12 | | 1.31 (1.15-1.50) | | <0.001 | | |  |
| Updated CAP^4^, unadjusted | Ref. | . |  | 1.89 (1.73-2.07) | <0.001 | 2.11 (1.69-2.63) | | <0.001 | | 3.03 (2.69-3.41) | | <0.001 | | |  |
| Updated CAP^4^, PS-adjusted | Ref. | . |  | 1.00 (0.91-1.09) | 0.95 | 1.16 (0.93-1.45) | | 0.18 | | 1.33 (1.18-1.50) | | <0.001 | | |  |
| ≥ 1 epilepsy or seizure dx, unadjusted | Ref. | . |  | 0.88 (0.55-1.41) | 0.58 | 0.29 (0.04-2.03) | | 0.21 | | 1.59 (1.12-2.27) | | <0.01 | | |  |
| ≥ 1 epilepsy or seizure dx, PS-adjusted | Ref. | . |  | 0.68 (0.42-1.11) | 0.16 | 0.31 (0.05-2.15) | | 0.24 | | 1.18 (0.83-1.68) | | 0.35 | | |  |
| No epilepsy or seizure dx, unadjusted | Ref. | . |  | 1.93 (1.75-2.11) | <0.001 | 2.23 (1.78-2.79) | | <0.001 | | 3.11 (2.74-3.53) | | <0.001 | | |  |
| No epilepsy or seizure dx, PS-adjusted | Ref. | . |  | 1.04 (0.95-1.14) | 0.43 | 1.29 (1.03-1.61) | | 0.03 | | 1.38 (1.22-1.56) | | <0.001 | | |  |
| ≥ 1 pain dx^2^, unadjusted | Ref. | . |  | 1.55 (1.39-1.72) | <0.001 | 1.80 (1.35-2.40) | | <0.001 | | 2.56 (2.24-2.93) | | <0.001 | | |  |
| ≥ 1 pain dx^2^, PS-adjusted | Ref. | . |  | 0.95 (0.86-1.06) | 0.38 | 1.13 (0.85-1.50) | | 0.41 | | 1.30 (1.13-1.48) | | <0.001 | | |  |
| No pain dx, unadjusted | Ref. | . |  | 2.05 (1.71-2.46) | <0.001 | 2.12 (1.49-3,02) | | <0.001 | | 2.95 (2.29-3.82) | | <0.001 | | |  |
| No pain dx, PS-adjusted | Ref. | . |  | 1.18 (0.99-1.42) | 0.07 | 1.33 (0.94-1.90) | | 0.11 | | 1.56 (1.21-2.01) | | <0.001 | | |  |
| T1: first trimester; PS: propensity score; RR: risk ratios; CI: confidence intervals; NICU: neonatal intensive care unit; Ref.: reference; Rx: filled prescription; dx: diagnosis; CAP: covariate assessment period  ^1^ Restricted to infants continuously eligible for ≥ 1 year ^2^ Includes neuropathic pain, fibromyalgia, arthritis, arthropathies and musculoskeletal pain, back and neck pain, migraine or headache, osteoarthritis, rheumatoid arthritis, or other pain  ^3^ Post-hoc analysis ^4^ CAP measured from the last menstrual period through the first 140 days of pregnancy | | | | | | | | | | | | | |  |  |
