# Supplementary material for: Gabapentin in pregnancy and the risk of adverse neonatal and maternal outcomes: A population-based cohort study nested in the US Medicaid Analytic eXtract dataset
Source: PLoS Med. 2020 Sep 1;17(9):e1003322. doi: 10.1371/journal.pmed.1003322 (PMC7462308; doi:10.1371/journal.pmed.1003322)
Supplement: S6 Table — RR, relative risk; T1, first trimester. (DOCX) [file pmed.1003322.s006.docx]

# S6 Table. Post-hoc analyses for the relative risk of specific types of cardiac malformations associated with exposure to gabapentin during the first trimester compared with unexposed pregnancies

| **Exposure Group:** | **Unexposed** | **Gabapentin-exposed during the first trimester** | | | | | | | |
| --- | --- | --- | --- | --- | --- | --- | --- | --- | --- |
| **Outcome:** |  | **Conotruncal defects** |  | **Single ventricle** |  | **Ventricular septal defect** |  | **Secundum atrial septal defect** |  |
|  |  | **RR (95% CI)** | **p-value** | **RR (95% CI)** | **p-value** | **RR (95% CI)** | **p-value** | **RR (95% CI)** | **p-value** |
| **Analysis** |  |  |  |  |  |  |  |  |  |
| PS-adjusted | Ref. | 1.99 (1.03-3.82) | 0.04 | 1.50 (0.21-10.66) | 0.69 | 0.89 (0.60-1.31) | 0.54 | 1.25 (0.80-1.96) | 0.33 |
| 2 Rx | Ref. | 2.77 (1.15-6.66) | 0.02 | . |  | 1.32 (0.81-2.14) | 0.27 | 1.16 (0.58-2.32) | 0.67 |
| Infant claims | Ref. | 2.07 (1.08-3.99) | 0.03 | 1.60 (0.22-11.40) | 0.64 | 0.88 (0.58-1.34) | 0.56 | 1.27 (0.80-2.02) | 0.31 |
| High-dimensional-PS-adjusted | Ref. | 1.92 (1.00-3.69) | 0.05 | 1.48 (0.21-10.56) | 0.69 | 0.89 (0.60-1.32) | 0.56 | 1.25 (0.80-1.96) | 0.33 |
| 2 Rx | Ref. | 2.77 (1.15-6.65) | 0.02 | . |  | 1.34 (0.82-2.18) | 0.25 | 1.12 (0.56-2.24) | 0.74 |
| Infant claims | Ref. | 1.98 (1.03-3.82) | 0.04 | 1.58 (0.22-11.23) | 0.65 | 0.90 (0.59-1.36) | 0.60 | 1.28 (0.81-2.04) | 0.29 |
| **Exposure Group:** | **Unexposed** | **Gabapentin-exposed during the first trimester** | | | | | | | |
|  |  | **Atrioventricular septal defect** |  | **Right-sided defects** |  | **Left-sided defects** |  | **Other cardiac defects** |  |
|  |  | **RR (95% CI)** | **p-value** | **RR (95% CI)** | **p-value** | **RR (95% CI)** | **p-value** | **RR (95% CI)** | **p-value** |
| **Analysis** |  |  |  |  |  |  |  |  |  |
| PS-adjusted | Ref. | 0.86 (0.12-6.13) | 0.88 | 1.09 (0.55-2.18) | 0.80 | 1.72 (0.997-2.96) | 0.05 | 1.50 (0.91-2.49) | 0.11 |
| 2 Rx | Ref. | . |  | 0.88 (0.28-2.74) | 0.83 | 1.79 (0.81-3.99) | 0.15 | 1.76 (0.88-3.52) | 0.11 |
| Infant claims | Ref. | 0.98 (0.14-6.94) | 0.98 | 0.98 (0.47-2.05) | 0.95 | 1.75 (0.99-3.08) | 0.05 | 1.41 (0.82-2.42) | 0.22 |
| High-dimensional-PS-adjusted | Ref. | 0.84 (0.12-5.97) | 0.86 | 1.06 (0.53-2.12) | 0.87 | 1.63 (0.94-2.80) | 0.08 | 1.46 (0.88-2.43) | 0.14 |
| 2 Rx | Ref. | . |  | 0.83 (0.27-2.59) | 0.75 | 1.66 (0.74-3.69) | 0.22 | 1.67 (0.84-3.33) | 0.15 |
| Infant claims | Ref. | 0.96 (0.13-6.81) | 0.97 | 0.95 (0.45-1.98) | 0.88 | 1.66 (0.94-2.93) | 0.08 | 1.38 (0.80-2.37) | 0.25 |
| PS: propensity score; RR: risk ratios; CI: confidence intervals; Ref.: reference; Rx: filled prescription; dx: diagnosis | | | | | | | | | |
