# Supplementary material for: Gabapentin in pregnancy and the risk of adverse neonatal and maternal outcomes: A population-based cohort study nested in the US Medicaid Analytic eXtract dataset
Source: PLoS Med. 2020 Sep 1;17(9):e1003322. doi: 10.1371/journal.pmed.1003322 (PMC7462308; doi:10.1371/journal.pmed.1003322)
Supplement: S7 Table — RR, relative risk. (DOCX) [file pmed.1003322.s007.docx]

# S7 Table. Sensitivity and secondary analyses for the relative risk of individual non-cardiac major malformation groups associated with exposure to gabapentin compared with unexposed pregnancies

| **Exposure Group:** | **Unexposed** | **Gabapentin-exposed during the first trimester** | | | | | | | |
| --- | --- | --- | --- | --- | --- | --- | --- | --- | --- |
| **Outcome:** |  | **Central nervous system defects** |  | **Eye anomalies** |  | **Ear anomalies** |  | **Non-cardiac vascular defects** |  |
|  |  | **RR (95% CI)** | **p-value** | **RR (95% CI)** | **p-value** | **RR (95% CI)** | **p-value** | **RR (95% CI)** | **p-value** |
| **Analysis** |  |  |  |  |  |  |  |  |  |
| PS-adjusted | Ref. | 1.53 (0.96-2.43) | 0.07 | 0.45 (0.06-3.23) | 0.43 | 1.77 (0.44-7.09) | 0.42 | 1.87 (0.78-4.50) | 0.16 |
| 2 Rx | Ref. | 1.61 (0.80-3.21) | 0.17 | . |  | 2.38 (0.33-16.94) | 0.39 | . |  |
| Infant claims | Ref. | 1.54 (0.96-2.48) | 0.07 | . |  | 1.88 (0.47-7.56) | 0.37 | 1.98 (0.82-4.77) | 0.13 |
| High-dimensional-PS-adjusted | Ref. | 1.56 (0.98-2.47) | 0.06 | 0.45 (0.06-3.23) | 0.43 | 1.89 (0.47-7.59) | 0.37 | 1.74 (0.72-4.19) | 0.22 |
| 2 Rx | Ref. | 1.61 (0.81-3.22) | 0.18 | . |  | 2.62 (0.37-18.62) | 0.34 | . |  |
| Infant claims | Ref. | 1.57 (0.98-2.53) | 0.06 | . |  | 2.01 (0.50-8.08) | 0.32 | 1.83 (0.76-4.40) | 0.18 |
| **Exposure Group:** | **Unexposed** | **Gabapentin-exposed during the first trimester** | | | | | | | |
| **Outcome:** |  | **Respiratory malformations** |  | **Oral clefts** |  | **Gastrointestinal malformations** |  | **Genital malformations** |  |
|  |  | **RR (95% CI)** | **p-value** | **RR (95% CI)** | **p-value** | **RR (95% CI)** | **p-value** | **RR (95% CI)** | **p-value** |
| **Analysis** |  |  |  |  |  |  |  |  |  |
| PS-adjusted | Ref. | 0.41 (0.20-0.87) | 0.02 | 1.25 (0.69-2.27) | 0.45 | 0.97 (0.69-1.36) | 0.86 | 1.25 (0.87-1.80) | 0.22 |
| 2 Rx | Ref. | 0.43 (0.14-1.35) | 0.15 | 1.00 (0.38-2.68) | 0.99 | 1.02 (0.62-1.69) | 0.94 | 1.33 (0.77-2.29) | 0.30 |
| Infant claims | Ref. | 0.43 (0.21-0.91) | 0.03 | 1.32 (0.73-2.39) | 0.35 | 0.96 (0.68-1.35) | 0.80 | 1.13 (0.75-1.70) | 0.55 |
| High-dimensional-PS-adjusted | Ref. | 0.41 (0.20-0.87) | 0.02 | 1.25 (0.69-2.26) | 0.46 | 0.96 (0.69-1.35) | 0.83 | 1.22 (0.85-1.76) | 0.27 |
| 2 Rx | Ref. | 0.43 (0.14-1.34) | 0.15 | 0.91 (0.34-2.42) | 0.85 | 1.03 (0.62-1.71) | 0.90 | 1.27 (0.74-2.18) | 0.40 |
| Infant claims | Ref. | 0.43 (0.21-0.90) | 0.03 | 1.31 (0.73-2.38) | 0.35 | 0.95 (0.67-1.34) | 0.77 | 1.11 (0.74-1.67) | 0.62 |
| **Exposure Group:** | **Unexposed** | **Gabapentin-exposed during the first trimester** | | | | | | | |
| **Outcome:** |  | **Urinary malformations** |  | **Musculo-skeletal malformations** |  | **Limb defects** |  | **Other malformations** |  |
|  |  | **RR (95% CI)** | **p-value** | **RR (95% CI)** | **p-value** | **RR (95% CI)** | **p-value** | **RR (95% CI)** | **p-value** |
| **Analysis** |  |  |  |  |  |  |  |  |  |
| PS-adjusted | Ref. | 0.97 (0.62-1.52) | 0.90 | 0.99 (0.71-1.40) | 0.97 | 1.25 (0.83-1.88) | 0.28 | 1.02 (0.51-2.04) | 0.96 |
| 2 Rx | Ref. | 0.47 (0.18-1.25) | 0.13 | 1.12 (0.69-1.83) | 0.64 | 1.21 (0.63-2.32) | 0.57 | 1.45 (0.60-3.48) | 0.41 |
| Infant claims | Ref. | 0.93 (0.57-1.52) | 0.78 | 0.96 (0.67-1.37) | 0.82 | 1.21 (0.78-1.87) | 0.40 | 1.13 (0.56-2.26) | 0.73 |
| High-dimensional-PS-adjusted | Ref. | 0.97 (0.62-1.52) | 0.88 | 1.00 (0.71-1.41) | 0.98 | 1.24 (0.82-1.86) | 0.31 | 0.94 (0.47-1.88) | 0.86 |
| 2 Rx | Ref. | 0.44 (0.17-1.18) | 0.10 | 1.13 (0.69-1.83) | 0.64 | 1.21 (0.63-2.32) | 0.57 | 1.36 (0.57-3.27) | 0.49 |
| Infant claims | Ref. | 0.93 (0.57-1.52) | 0.78 | 0.97 (0.68-1.38) | 0.86 | 1.20 (0.77-1.86) | 0.42 | 1.04 (0.52-2.08) | 0.92 |
| PS: propensity score; RR: risk ratios; CI: confidence intervals; Ref.: reference; Rx: filled prescription; dx: diagnosis | | | | | | | | | |
