# Supplementary material for: Gabapentin in pregnancy and the risk of adverse neonatal and maternal outcomes: A population-based cohort study nested in the US Medicaid Analytic eXtract dataset
Source: PLoS Med. 2020 Sep 1;17(9):e1003322. doi: 10.1371/journal.pmed.1003322 (PMC7462308; doi:10.1371/journal.pmed.1003322)
Supplement: S8 Table — RR, relative risk. (DOCX) [file pmed.1003322.s008.docx]

# S8 Table. Relative risk of cardiac malformations comparing gabapentin exposed to unexposed women, stratified by dose tertiles of the first and the highest prescription filled during each exposure period of interest

| **Exposure Group:** | **Unexposed** | **Exposed during T1** |  | **Exposed  early in pregnancy** |  | **Exposed  late in pregnancy** |  | **Exposed  early and late in pregnancy** |  |
| --- | --- | --- | --- | --- | --- | --- | --- | --- | --- |
| **Outcomes** |  | **PS-adjusted  RR (95% CI)** | **p-value** | **PS-adjusted  RR (95% CI)** | **p-value** | **PS-adjusted  RR (95% CI)** | **p-value** | **PS-adjusted  RR (95% CI)** | **p-value** |
| **Major congenital malformations** |  |  |  |  |  |  |  |  |  |
| Main PS-adjusted analysis | **Ref.** | **1.07 (0.94-1.21)** | **0.33** | . |  | . |  | . |  |
| Gabapentin (mg/day), first dose |  |  |  |  |  |  |  |  |  |
| <600 | Ref. | 1.05 (0.82-1.34) | 0.72 | . |  | . |  | . |  |
| 600-900 | Ref. | 1.00 (0.80-1.24) | 0.98 | . |  | . |  | . |  |
| >900 | Ref. | 1.17 (0.95-1.44) | 0.15 | . |  | . |  | . |  |
| Gabapentin (mg/day), highest dose |  |  |  |  |  |  |  |  |  |
| ≤600 | Ref. | 1.00 (0.79-1.27) | 0.98 | . |  | . |  | . |  |
| 601-1200 | Ref. | 1.01 (0.79-1.30) | 0.94 | . |  | . |  | . |  |
| >1200 | Ref. | 1.16 (0.95-1.41) | 0.15 | . |  | . |  | . |  |
| **Cardiac malformations** |  |  |  |  |  |  |  |  |  |
| Main PS-adjusted analysis | **Ref.** | **1.12 (0.89-1.40)** | **0.35** | . |  | . |  | . |  |
| Gabapentin (mg/day), first dose |  |  |  |  |  |  |  |  |  |
| <600 | Ref. | 0.87 (0.52-1.43) | 0.57 | . |  | . |  | . |  |
| 600-900 | Ref. | 1.35 (0.97-1.90) | 0.08 | . |  | . |  | . |  |
| >900 | Ref. | 1.05 (0.70-1.56) | 0.82 | . |  | . |  | . |  |
| Gabapentin (mg/day), highest dose |  |  |  |  |  |  |  |  |  |
| ≤600 | Ref. | 0.81 (0.51-1.31) | 0.39 | . |  | . |  | . |  |
| 601-1200 | Ref. | 1.48 (1.01-2.16) | 0.04 | . |  | . |  | . |  |
| >1200 | Ref. | 1.10 (0.77-1.58) | 0.60 | . |  | . |  | . |  |
| **Preeclampsia** |  |  |  |  |  |  |  |  |  |
| Main PS-adjusted analysis | **Ref.** | . |  | **0.87 (0.75-1.00)** | **0.05** | **0.96 (0.69-1.33)** | **0.80** | **0.92 (0.74-1.13)** | **0.42** |
| Gabapentin (mg/day), first dose |  |  |  |  |  |  |  |  |  |
| <600 | Ref. | . |  | 0.76 (0.57-1.00) | 0.05 | 0.77 (0.42-1.41) | 0.40 | 1.31 (0.86-1.99) | 0.21 |
| 600-900 | Ref. | . |  | 0.89 (0.72-1.10) | 0.29 | 1.10 (0.69-1.74) | 0.69 | 0.91 (0.62-1.32) | 0.61 |
| >900 | Ref. | . |  | 0.95 (0.73-1.24) | 0.73 | 0.99 (0.46-2.17) | 0.99 | 0.79 (0.57-1.09) | 0.15 |
| Gabapentin (mg/day), highest dose |  |  |  |  |  |  |  |  |  |
| ≤600 | Ref. | . |  | 0.79 (0.62-1.00) | 0.05 | 0.72 (0.42-1.22) | 0.22 | 1.24 (0.75-2.04) | 0.41 |
| 601-1200 | Ref. | . |  | 0.88 (0.68-1.13) | 0.30 | 1.14 (0.68-1.93) | 0.61 | 1.26 (0.83-1.92) | 0.28 |
| >1200 | Ref. | . |  | 0.97 (0.76-1.24) | 0.81 | 1.33 (0.65-2.71) | 0.44 | 0.77 (0.58-1.02) | 0.06 |
| **Preterm delivery** |  |  |  |  |  |  |  |  |  |
| Main PS-adjusted analysis | **Ref.** | . |  | **1.00 (0.93-1.08)** | **0.89** | **1.28 (1.08-1.52)** | **<0.01** | **1.22 (1.09-1.36)** | **<0.001** |
| Gabapentin (mg/day), first dose |  |  |  |  |  |  |  |  |  |
| <600 | Ref. | . |  | 1.03 (0.90-1.18) | 0.65 | 1.38 (1.05-1.81) | 0.02 | 1.23 (0.95-1.59) | 0.12 |
| 600-900 | Ref. | . |  | 1.04 (0.93-1.17) | 0.49 | 1.37 (1.07-1.75) | 0.01 | 1.23 (1.01-1.49) | 0.04 |
| >900 | Ref. | . |  | 0.93 (0.80-1.07) | 0.31 | 0.95 (0.59-1.53) | 0.84 | 1.21 (1.04-1.41) | 0.01 |
| Gabapentin (mg/day), highest dose |  |  |  |  |  |  |  |  |  |
| ≤600 | Ref. | . |  | 1.08 (0.96-1.21) | 0.19 | 1.42 (1.13-1.79) | <0.01 | 1.14 (0.83-1.58) | 0.41 |
| 601-1200 | Ref. | . |  | 0.99 (0.86-1.14) | 0.91 | 1.23 (0.92-1.65) | 0.17 | 1.04 (0.79-1.37) | 0.77 |
| >1200 | Ref. | . |  | 0.93 (0.80-1.07) | 0.29 | 0.97 (0.59-1.62) | 0.92 | 1.29 (1.14-1.47) | <0.001 |
| **Small for gestational age (SGA)** |  |  |  |  |  |  |  |  |  |
| Main PS-adjusted analysis | **Ref.** | . |  | **1.17 (1.02-1.33)** | **0.02** | **1.39 (1.01-1.91)** | **0.05** | **1.32 (1.08-1.60)** | **<0.01** |
| Gabapentin (mg/day), first dose |  |  |  |  |  |  |  |  |  |
| <600 | Ref. | . |  | 1.28 (1.02-1.61) | 0.03 | 1.03 (0.54-1.95) | 0.93 | 2.22 (1.56-3.17) | <0.001 |
| 600-900 | Ref. | . |  | 1.16 (0.94-1.43) | 0.17 | 1.95 (1.30-2.93) | <0.01 | 1.05 (0.70-1.58) | 0.80 |
| >900 | Ref. | . |  | 1.04 (0.80-1.36) | 0.76 | 0.77 (0.29-2.01) | 0.59 | 1.15 (0.86-1.54) | 0.34 |
| Gabapentin (mg/day), highest dose |  |  |  |  |  |  |  |  |  |
| ≤600 | Ref. | . |  | 1.32 (1.08-1.62) | <0.01 | 1.29 (0.79-2.12) | 0.30 | 1.78 (1.12-2.85) | 0.02 |
| 601-1200 | Ref. | . |  | 1.14 (0.89-1.46) | 0.30 | 1.71 (1.05-2.77) | 0.03 | 1.41 (0.89-2.23) | 0.14 |
| >1200 | Ref. | . |  | 1.01 (0.78-1.29) | 0.96 | 0.84 (0.32-2.19) | 0.72 | 1.20 (0.93-1.53) | 0.16 |
| **NICU admission** |  |  |  |  |  |  |  |  |  |
| Main PS-adjusted analysis | **Ref.** | . |  | **1.01 (0.93-1.11)** | **0.77** | **1.21 (0.97-1.51)** | **0.09** | **1.35 (1.20-1.52)** | **<0.001** |
| Gabapentin (mg/day), first dose |  |  |  |  |  |  |  |  |  |
| <600 | Ref. | . |  | 1.02 (0.86-1.20) | 0.85 | 1.59 (1.15-2.20) | <0.01 | 1.41 (1.06-1.87) | 0.02 |
| 600-900 | Ref. | . |  | 1.03 (0.89-1.19) | 0.67 | 0.99 (0.69-1.44) | 0.97 | 1.29 (1.03-1.62) | 0.02 |
| >900 | Ref. | . |  | 0.99 (0.83-1.18) | 0.94 | 1.12 (0.66-1.91) | 0.68 | 1.35 (1.15-1.59) | <0.001 |
| Gabapentin (mg/day), highest dose |  |  |  |  |  |  |  |  |  |
| ≤600 | Ref. | . |  | 1.01 (0.87-1.17) | 0.90 | 1.47 (1.10-1.96) | <0.01 | 1.18 (0.81-1.71) | 0.39 |
| 601-1200 | Ref. | . |  | 1.10 (0.94-1.30) | 0.23 | 0.87 (0.55-1.36) | 0.53 | 1.07 (0.77-1.49) | 0.67 |
| >1200 | Ref. | . |  | 0.94 (0.80-1.11) | 0.45 | 1.22 (0.71-2.07) | 0.47 | 1.45 (1.26-1.66) | <0.001 |
| T1: first trimester; PS: propensity score; RR: risk ratios; CI: confidence intervals; Ref.: reference | | | | | | | | | |
