# Supplementary figures and images for: Gabapentin in pregnancy and the risk of adverse neonatal and maternal outcomes: A population-based cohort study nested in the US Medicaid Analytic eXtract dataset
Source: PLoS Med. 2020 Sep 1;17(9):e1003322. doi: 10.1371/journal.pmed.1003322 (PMC7462308; doi:10.1371/journal.pmed.1003322)

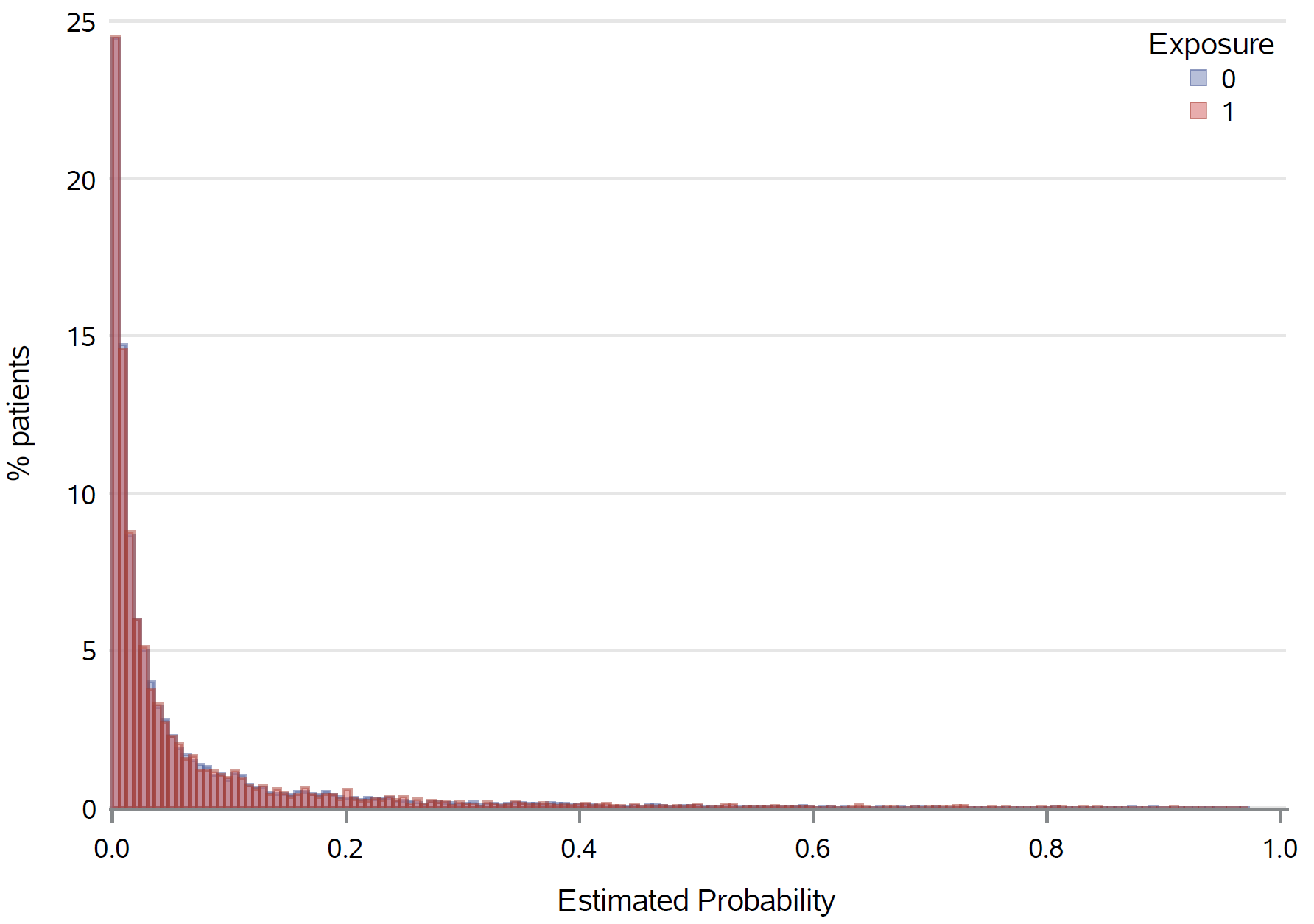

Supplement: S1 Fig — PS, propensity score; T1, first trimester. (TIF) [file pmed.1003322.s009.tif]

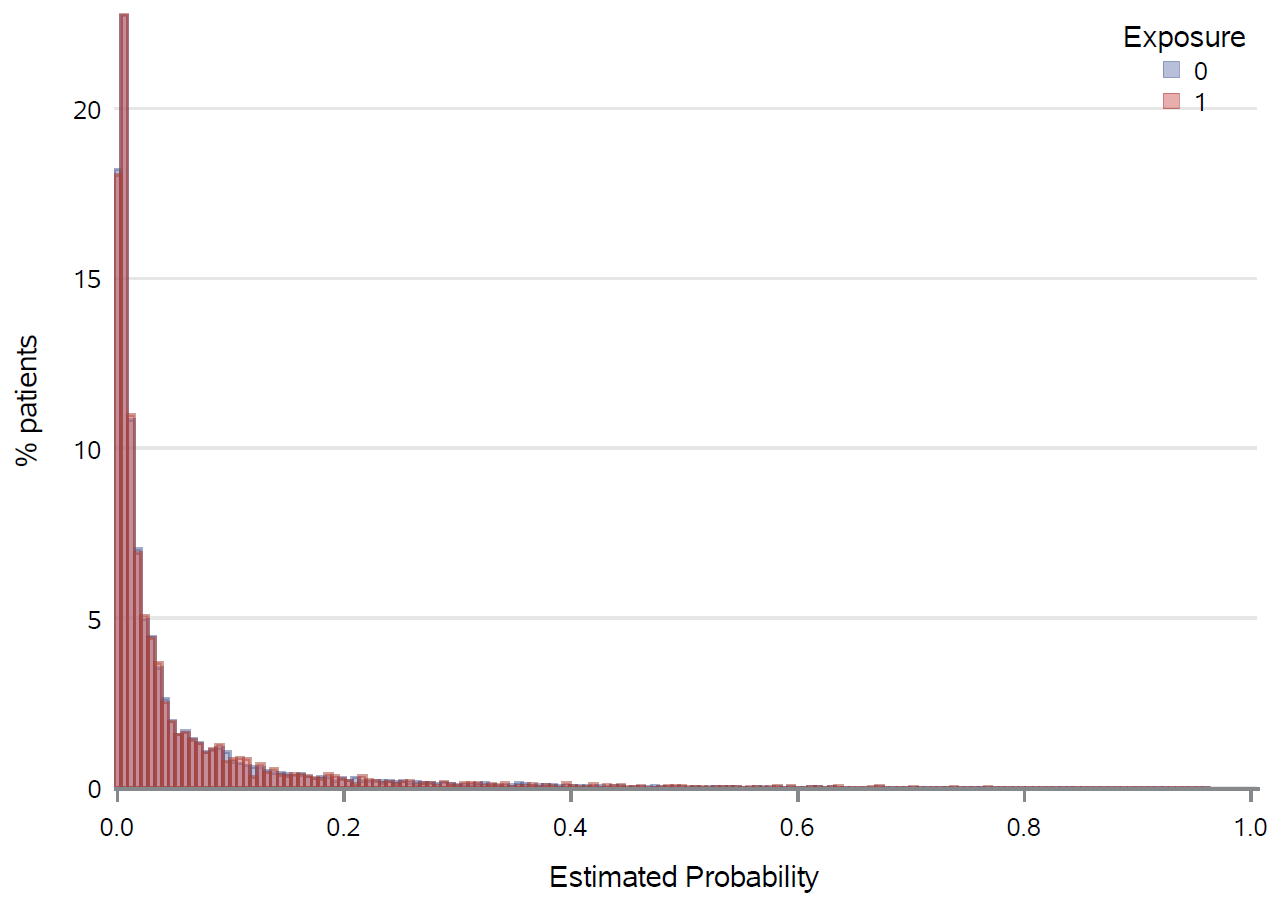

Supplement: S2 Fig — PS, propensity score. (TIF) [file pmed.1003322.s010.tif]

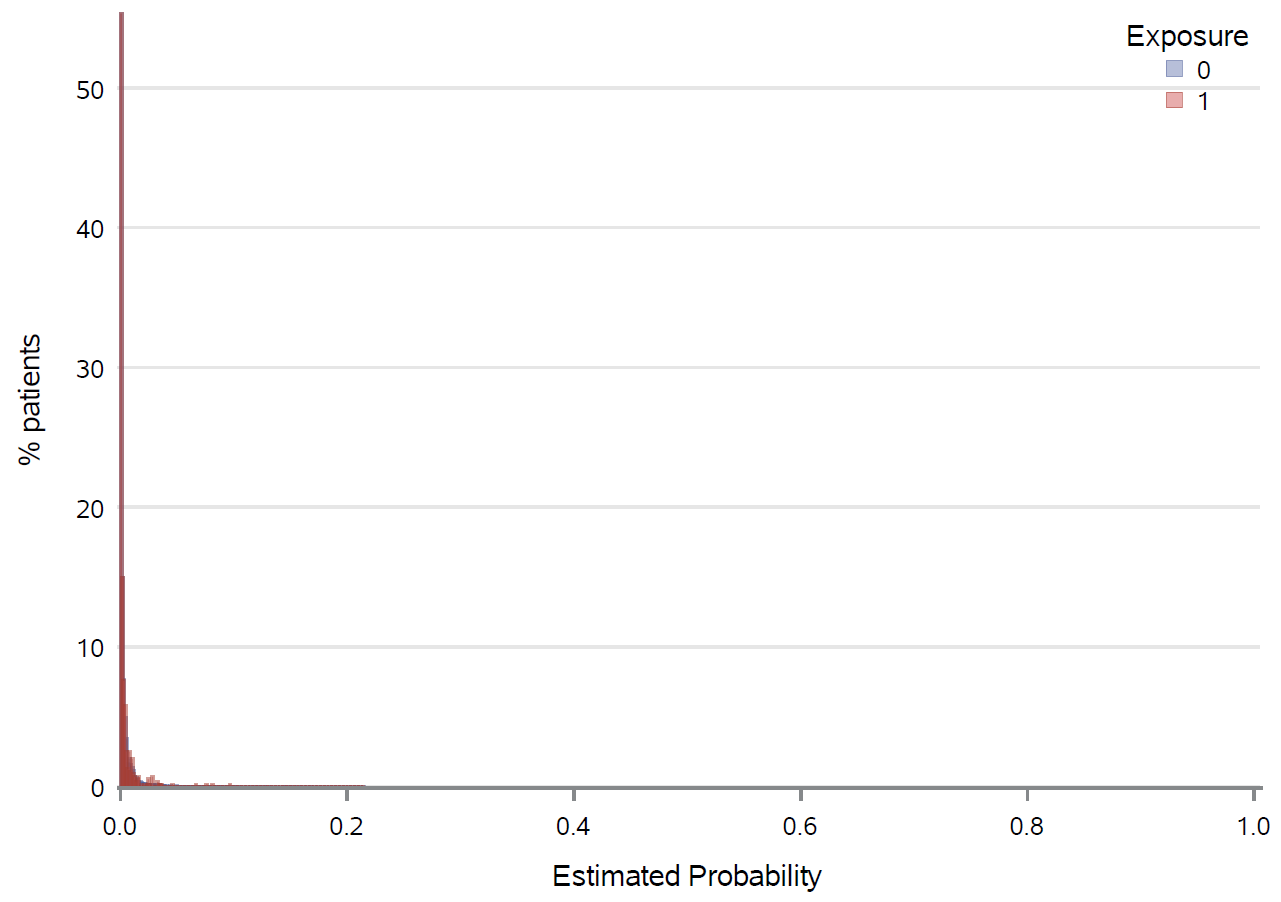

Supplement: S3 Fig — PS, propensity score. (TIF) [file pmed.1003322.s011.tif]

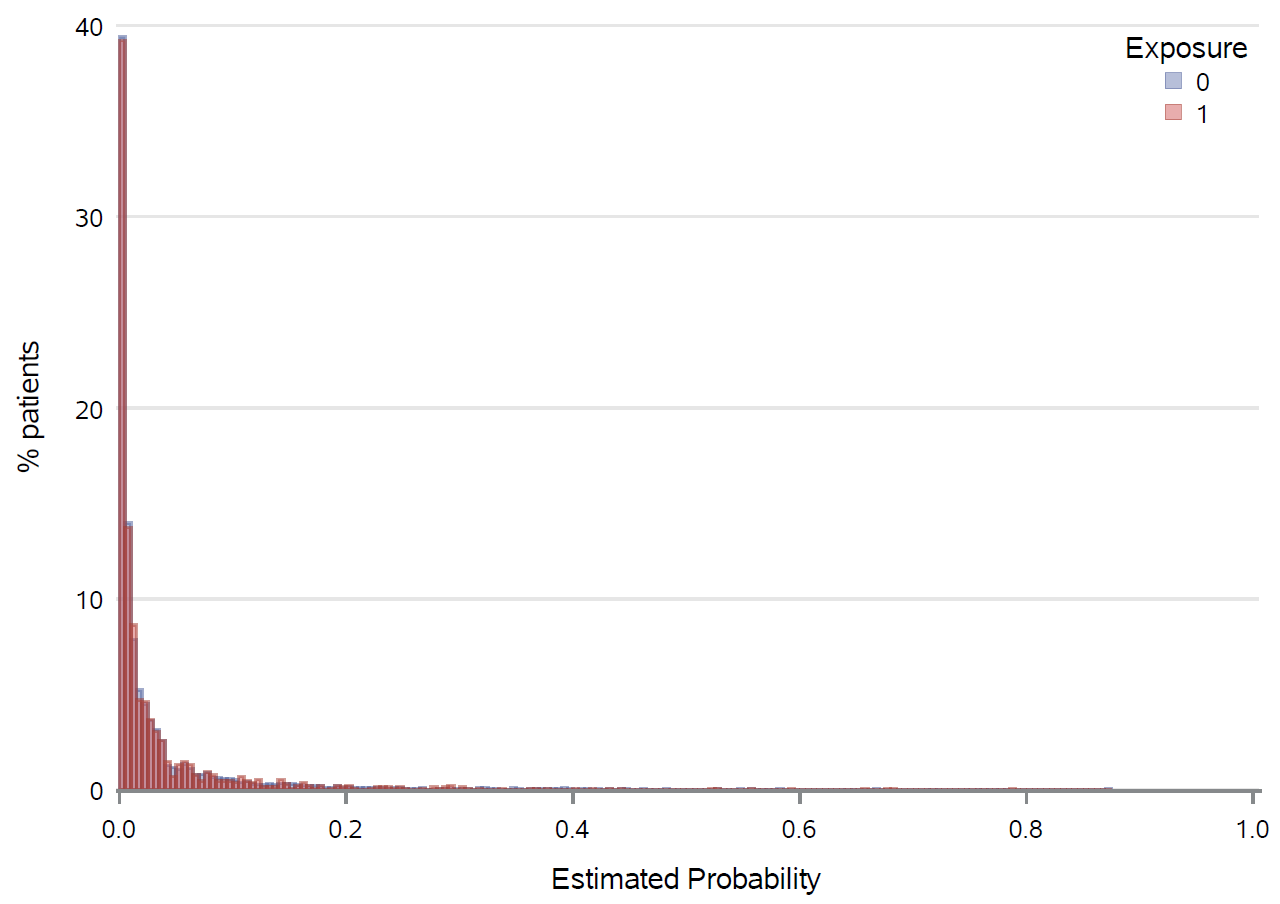

Supplement: S4 Fig — PS, propensity score. (TIF) [file pmed.1003322.s012.tif]

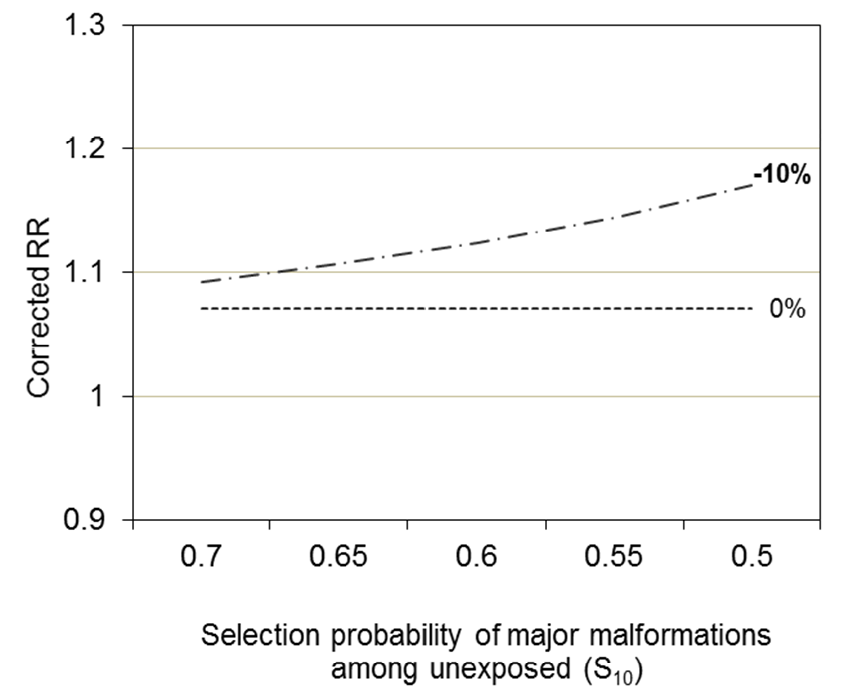

Supplement: S5 Fig — RR, relative risk; T1, first trimester. (TIF) [file pmed.1003322.s013.tif]
