# Supplementary material for: Gabapentin in pregnancy and the risk of adverse neonatal and maternal outcomes: A population-based cohort study nested in the US Medicaid Analytic eXtract dataset
Source: PLoS Med. 2020 Sep 1;17(9):e1003322. doi: 10.1371/journal.pmed.1003322 (PMC7462308; doi:10.1371/journal.pmed.1003322)
Supplement: S1 Text — (DOCX) [file pmed.1003322.s014.docx]

# S1 Text. Planned analyses

Although we did not formally register a pre-specified analytical plan prior to conducting this study, all analyses presented in the manuscript were pre-planned in a specific protocol, except for the post-hoc sensitivity analyses that we added in order to explore some of the findings of our prespecified analyses. Below, we provide a summary of the planned study design and analyses.

**Summary of study design**

| **Exposure** | **Specific outcome^1^:** | **Reference** | **Outcome** | **Covariates** | |
| --- | --- | --- | --- | --- | --- |
| ≥1 gabapentin fill, LMP through T1 | **Malformations** | Gabapentin non-fill in pre-LMP through delivery  (without excluding users of other anticonvulsants) | Delivery through 3 months (depending on infant or maternal codes) | 3 months pre-LMP through the end of T1, except for healthcare utilization (3 months pre-LMP through LMP) | |
| ≥1 gabapentin fill, LMP through 140 days  NOT  ≥1 gabapentin fill, 141-245 days | **Preeclampsia** |  | Delivery through 30 days after delivery (depending on infant or maternal codes) | Primary analyses:  3 months pre-LMP through the end of T1, except for healthcare utilization (3 months pre-LMP through LMP) | Secondary analyses:  3 months pre-LMP through 245 days, except for healthcare utilization (LMP through 140 days) |
|  | **Preterm delivery** |  |  |  |  |
|  | **Small for gestational age** |  |  |  |  |
|  | **NICU admission** |  |  |  |  |
| ≥1 gabapentin fill, 141-245 days  NOT  ≥1 gabapentin fill, LMP through 140 days | **Preeclampsia** |  |  |  |  |
|  | **Preterm delivery** |  |  |  |  |
|  | **Small for gestational age** |  |  |  |  |
|  | **NICU admission** |  |  |  |  |
| ≥1 gabapentin fill, LMP through 140 days  AND  ≥1 gabapentin fill, 141-245 days | **Preeclampsia** |  |  |  |  |
|  | **Preterm delivery** |  |  |  |  |
|  | **Small for gestational age** |  |  |  |  |
|  | **NICU admission** |  |  |  |  |

LMP: last menstrual period; T1: first trimester; NICU: neonatal intensive care unit.

^1^See eTable 1 and eTable 2 for specific outcome definitions

**Summary of analytical plan**

Primary Analysis

We proposed to assess the prevalence of covariates by exposure group and to use standardized differences to evaluate covariate balance between the exposed pregnancies and the reference group. We planned to calculate absolute risks of outcomes and unadjusted risk ratios (RR) with 95% confidence interval (CI).

In order to adjust for confounding, we proposed to estimate propensity scores (PS) in logistic regression models as the predicted probability of receiving gabapentin conditional upon the previously described covariates. This was planned to be implemented separately for each exposure group. We proposed to trim the population whose PS fell within the non-overlapping areas of the PS distributions, and to create 50 PS-strata according to the distribution of the exposed pregnancies. We planned to calculate weights for the reference group of unexposed pregnancies on the basis of the distribution of the exposed in PS-strata and to estimate adjusted RR and 95% CI in generalized linear models. We also planned to test the use of the robust variance estimator to account for correlations within women with multiple pregnancies.

Sensitivity Analyses

We planned on conducting several sensitivity analyses to assess the robustness of primary findings:

1. In order to reduce the potential for exposure misclassification, we decided to update the exposure definition as filling 2 or more gabapentin prescriptions during each of the specified periods of interest, with the assumption that filling multiple prescriptions would increase the likelihood that gabapentin was actually taken or was taken more consistently.
2. Second, to ensure that maternal malformations were not being erroneously attributed to the offspring, and thus reduce the chances of outcome misclassification, we planned to re-define the outcome of major malformations using infant claims only.
3. Third, to reduce potential residual confounding due to channeling bias, we proposed to 1) use high-dimensional PS (hdPS), to enrich the original PS with 100 additional empirically identified covariates, and 2) to conduct analyses restricted to pregnancies among subgroups of women with either indication of epilepsy or seizures or indication of pain.
4. Fourth, for exposure to gabapentin early, late, and both early and late in pregnancy, to ensure potential confounders beyond the end of the first trimester were captured and accounted for, we decided to update the covariate assessment period by measuring maternal comorbidities, concomitant medication, and healthcare intensity from the last menstrual period through the first 140 days of pregnancy.
5. Fifth, to assess the presence of a potential dose-response relationship for gabapentin, we planned to examine the risk of outcomes according to tertiles of the first and the highest prescribed daily dose filled during the specific exposure period of interest.
6. Sixth, for the malformation outcome, since we decided to only include live births in our study cohort, we planned to examine the potential impact of differences in the proportion of terminations among women exposed to gabapentin versus those unexposed on the primary PS-adjusted estimate.
7. Seventh, as maternal smoking has been identified as an important predictor of preterm birth, SGA, and preeclampsia, and is not completely captured in claims data, we proposed to quantify the potential impact of residual confounding by smoking on preterm birth, SGA, and preeclampsia in a bias analysis. This analysis aimed at quantifying the impact of varying the imbalance in the prevalence of maternal smoking between gabapentin-exposed and unexposed pregnancies on the primary PS-adjusted estimates.
