# Supplementary material for: Gabapentin in pregnancy and the risk of adverse neonatal and maternal outcomes: A population-based cohort study nested in the US Medicaid Analytic eXtract dataset
Source: PLoS Med. 2020 Sep 1;17(9):e1003322. doi: 10.1371/journal.pmed.1003322 (PMC7462308; doi:10.1371/journal.pmed.1003322)
Supplement: S1 STROBE Checklist — (DOCX) [file pmed.1003322.s015.docx]

# S1 STROBE Checklist

STROBE Statement—Checklist of items that should be included in reports of ***cohort studies***

|  | Item No | Recommendation |
| --- | --- | --- |
| **Title and abstract** | 1 | (*a*) Indicate the study’s design with a commonly used term in the title or the abstract  YES (Title page; Abstract, Methods and findings) |
|  |  | (*b*) Provide in the abstract an informative and balanced summary of what was done and what was found  YES (Abstract, Methods and findings) |
| Introduction | | |
| Background/rationale | 2 | Explain the scientific background and rationale for the investigation being reported  YES (Introduction, paragraphs 1-3) |
| Objectives | 3 | State specific objectives, including any prespecified hypotheses  YES (Introduction, paragraph 1) |
| Methods | | |
| Study design | 4 | Present key elements of study design early in the paper  YES (Methods, Source of Data and Study Population, page 6, paragraph 1; Methods, Exposure definition, paragraph 1) |
| Setting | 5 | Describe the setting, locations, and relevant dates, including periods of recruitment, exposure, follow-up, and data collection  YES (Methods, Source of Data and Study Population, paragraph 1) |
| Participants | 6 | (*a*) Give the eligibility criteria, and the sources and methods of selection of participants. Describe methods of follow-up  YES (Methods, Source of Data and Study Population, paragraph 1) |
|  |  | (*b*) For matched studies, give matching criteria and number of exposed and unexposed  N/A |
| Variables | 7 | Clearly define all outcomes, exposures, predictors, potential confounders, and effect modifiers. Give diagnostic criteria, if applicable  YES (Methods, Exposure definition, paragraph 1; Methods, Outcomes, paragraph 2; Methods, Covariates, paragraph 2) |
| Data sources/ measurement | 8* | For each variable of interest, give sources of data and details of methods of assessment (measurement). Describe comparability of assessment methods if there is more than one group  YES (Methods, Exposure definition, paragraph 1; Methods, Outcomes, paragraph 2; Methods, Covariates, paragraph 2) |
| Bias | 9 | Describe any efforts to address potential sources of bias  YES (Methods, Primary Analysis, paragraph 1; Methods, Sensitivity Analyses, paragraph 2) |
| Study size | 10 | Explain how the study size was arrived at  YES (Methods, Source of Data and Study Population, paragraph 1; Results, paragraph 1) |
| Quantitative variables | 11 | Explain how quantitative variables were handled in the analyses. If applicable, describe which groupings were chosen and why  YES (Methods, Primary Analysis, paragraph 1; Methods, Sensitivity Analyses, paragraph 2) |
| Statistical methods | 12 | 1. Describe all statistical methods, including those used to control for confounding   YES (Methods, Primary Analysis, paragraph 1; Methods, Sensitivity Analyses, paragraph 2) |
|  |  | (*b*) Describe any methods used to examine subgroups and interactions  YES (Methods, Primary Analysis, paragraph 1; Methods, Sensitivity Analyses, paragraph 2) |
|  |  | (*c*) Explain how missing data were addressed  N/A |
|  |  | (*d*) If applicable, explain how loss to follow-up was addressed  YES (Methods, Source of Data and Study Population, paragraph 1) |
|  |  | (*e*) Describe any sensitivity analyses  YES (Methods, Sensitivity Analyses, paragraph 2) |
| Results | | |
| Participants | 13* | (a) Report numbers of individuals at each stage of study—eg numbers potentially eligible, examined for eligibility, confirmed eligible, included in the study, completing follow-up, and analysed  YES (Results, paragraph 1) |
|  |  | (b) Give reasons for non-participation at each stage  YES (Figure 1) |
|  |  | (c) Consider use of a flow diagram  YES (Figure 1) |
| Descriptive data | 14* | (a) Give characteristics of study participants (eg demographic, clinical, social) and information on exposures and potential confounders  YES (Results, paragraph 2; Table 1, eTable 3, eTable 4) |
|  |  | (b) Indicate number of participants with missing data for each variable of interest  N/A |
|  |  | (c) Summarise follow-up time (eg, average and total amount)  N/A |
| Outcome data | 15* | Report numbers of outcome events or summary measures over time  YES (Results, Absolute and relative risks of neonatal and maternal outcomes, paragraph 3) |
| Main results | 16 | (*a*) Give unadjusted estimates and, if applicable, confounder-adjusted estimates and their precision (eg, 95% confidence interval). Make clear which confounders were adjusted for and why they were included  YES (Results, Absolute and relative risks of neonatal and maternal outcomes, paragraph 3) |
|  |  | (*b*) Report category boundaries when continuous variables were categorized  N/A |
|  |  | (*c*) If relevant, consider translating estimates of relative risk into absolute risk for a meaningful time period  YES (Results, Absolute and relative risks of neonatal and maternal outcomes, paragraph 3) |
| Other analyses | 17 | Report other analyses done—eg analyses of subgroups and interactions, and sensitivity analyses  YES (Results, Sensitivity, secondary and post-hoc analyses, paragraph 1) |
| Discussion | | |
| Key results | 18 | Summarise key results with reference to study objectives  YES (Discussion, paragraph 2) |
| Limitations | 19 | Discuss limitations of the study, taking into account sources of potential bias or imprecision. Discuss both direction and magnitude of any potential bias  YES (Discussion, paragraph 2) |
| Interpretation | 20 | Give a cautious overall interpretation of results considering objectives, limitations, multiplicity of analyses, results from similar studies, and other relevant evidence  YES (Discussion, paragraphs 1 and 2) |
| Generalisability | 21 | Discuss the generalisability (external validity) of the study results  YES (Discussion, paragraph 1) |
| Other information | | |
| Funding | 22 | Give the source of funding and the role of the funders for the present study and, if applicable, for the original study on which the present article is based  YES (Funding) |

*Give information separately for exposed and unexposed groups.

**Note:** An Explanation and Elaboration article discusses each checklist item and gives methodological background and published examples of transparent reporting. The STROBE checklist is best used in conjunction with this article (freely available on the Web sites of PLoS Medicine at http://www.plosmedicine.org/, Annals of Internal Medicine at http://www.annals.org/, and Epidemiology at http://www.epidem.com/). Information on the STROBE Initiative is available at http://www.strobe-statement.org.
