# Supplementary material for: Gabapentin in pregnancy and the risk of adverse neonatal and maternal outcomes: A population-based cohort study nested in the US Medicaid Analytic eXtract dataset
Source: PLoS Med. 2020 Sep 1;17(9):e1003322. doi: 10.1371/journal.pmed.1003322 (PMC7462308; doi:10.1371/journal.pmed.1003322)
Supplement: Attachment — Submitted filename: Response_Gabapentin_PLOS_Med_Mar 6 2020.docx [file pmed.1003322.s016.docx]

***Elisabetta Patorno, M.D., Dr.P.H. Assistant Professor of Medicine***

***epatorno@bwh.harvard.edu***

Requests from the editors:

1.Data Availability Statement: Thank you for your willingness to include all relevant data within the manuscript and supporting information files. As noted by reviewer 3, the data are not present in their entirety. Please modify your statement for the data used in your study:

a) If the data are freely or publicly available, note this and state the location of the data: within the paper, in Supporting Information files, or in a public repository (include the DOI or accession number).

b) If the data are owned by a third party but freely available upon request, please note this and state the owner of the data set and contact information for data requests (web or email address). Note that a study author cannot be the contact person for the data.

c) If the data are not freely available, please describe briefly the ethical, legal, or contractual restriction that prevents you from sharing it. Please also include an appropriate contact (web or email address) for inquiries (again, this cannot be a study author).

[Response] We have included the following Data Availability Statement in the manuscript: “Because of the data use agreement in place, the research team cannot share the database used for the current paper, which was based on Medicaid, a joint federal and state program that helps provide health care coverage for people with low incomes and limited resources in the United States. Other researchers may request to gain access to the Medicaid database through the Research Data Assistance Center (ResDAC) (<https://www.resdac.org/>)”

2. Prospective Analysis Plan: Did your study have a prospective protocol or analysis plan? Please state this (either way) early in the Methods section.

a) If a prospective analysis plan (from your funding proposal, IRB or other ethics committee submission, study protocol, or other planning document written before analyzing the data) was used in designing the study, please include the relevant prospectively written document with your revised manuscript as a Supporting Information file to be published alongside your study, and cite it in the Methods section. A legend for this file should be included at the end of your manuscript.

b) If no such document exists, please make sure that the Methods section transparently describes when analyses were planned, and when/why any data-driven changes to analyses took place.

c) In either case, changes in the analysis—including those made in response to peer review comments—should be identified as such in the Methods section of the paper, with rationale

[Response] We now include a description of our planned analyses in the Appendix.

3. Title: Please revise your title according to PLOS Medicine's style. Your title must be nondeclarative and not a question. It should begin with main concept if possible. "Effect of" should be used only if causality can be inferred, i.e., for an RCT. Please place the study design ("A randomized controlled trial," "A retrospective study," "A modelling study," etc.) in the subtitle (ie, after a colon).

[Response] We have updated the title as “Gabapentin in Pregnancy and the Risk of Adverse Neonatal and Maternal Outcomes: A Population-based Cohort Study”.

4. Abstract: Please structure your abstract using the PLOS Medicine headings (Background, Methods and Findings, Conclusions).

[Response] We restructured the abstract accordingly.

5. Abstract: Please include the study design, population and setting, years during which the study took place,and length of follow up.

[Response] We have revised the abstract accordingly, which now reads as:

“Methods and findings: Using the MAX dataset, we conducted a population-based study of 1,753,865 Medicaid-eligible pregnancies between 2000 and 2013…”

6. Abstract: Methods and Findings: Please list some examples of the 70 confounders most critical to the study.

[Response] We now provide some examples of covariates:

“We estimated relative risks (RR) and 95% confidence intervals (CI) using fine stratification on the propensity score to control for over 70 confounders, e.g., maternal age, race/ethnicity, indications for gabapentin, other pain conditions, hypertension, diabetes, use of opioids and specific morphine equivalents.”

7. Abstract: Methods and Findings: Of the 1,753,865: please also clarify numbers for the unexposed (reference) group.

[Response] We now provide numbers for the unexposed group:

“We identified 4,642 pregnancies exposed in T1, 3,745 exposed in early pregnancy only, 556 exposed in late pregnancy only, and 1,275 exposed in both early and late pregnancy. The reference group consisted of 1,744,447 unexposed pregnancies.”

8. Abstract: Methods and Findings: For all results quantified regarding risk associated with exposure, please present both the 95% CIs and p values.

[Response] We would prefer, if possible, to only report 95% confidence intervals in the abstract and throughout the manuscript. Confidence intervals are very informative measures as they provide information on both magnitude of the effect and degree of precision of the estimates. On the contrary, p values are not particularly informative statistics as they do not allow to disentangle precision of the estimates with effect size, ultimately confusing these two essential aspects of data interpretation. For these reasons, significance tests such as p-values have been interpreted as a suboptimal classification scheme of results (*Rothman KJ. Six persistent research misconceptions. J Gen Intern Med. 2014;29(7):1060–1064. doi:10.1007/s11606-013-2755-z; Wasserstein & Lazar. American Statistical Association’s statement on p-values. Am Stat 70.2 (2016): 129-133; Greenland, S., Senn, S.J., Rothman, K.J. et al. Statistical tests, P values, confidence intervals, and power: a guide to misinterpretations. Eur J Epidemiol 31, 337–350 (2016).* [*https://doi.org/10.1007/s10654-016-0149-3*](https://doi.org/10.1007/s10654-016-0149-3))

Nevertheless, if the Editor prefers p-values to be included in the manuscript, we would be happy to engage in further discussion regarding this possibility.

9. Abstract: Methods and Findings: Please clarify if results presented are for those exposed both early and late in pregnancy, or if these are combined effects of early and late gabapentin exposure on PTB, SGA, and NICUa, and if so, please present separate results for early vs. late exposure (with 95% CIs and p values).

[Response] Please see the explanation provided above.

10. Abstract: Methods and Findings: In the last sentence of the Abstract Methods and Findings section, please describe the main limitation(s) of the study's methodology.

[Response] We have added limitations in the last sentence of the Abstract Methods and Findings section:

“Our study had limitations, including potential residual confounding and exposure misclassification.”

11. Abstract: Conclusions: Please address the study implications without overreaching what can be concluded from the data; the phrase "In this study, we observed ..." may be useful.

[Response] We have revised our Conclusions as:

“In this large population-based study, we found that gabapentin exposure during early pregnancy did not appear to be associated with strong teratogenic effects, though a moderate increase in the risk of cardiac malformations could not be excluded. Maternal use of gabapentin, particularly late in pregnancy, was associated with an increased risk of PTB, SGA, and NICUa.”

12. Author Summary: At this stage, we ask that you include a short, non-technical Author Summary of your research to make findings accessible to a wide audience that includes both scientists and non-scientists. The Author Summary should immediately follow the Abstract in your revised manuscript. This text is subject to editorial change and should be distinct from the scientific abstract. Please see our author guidelines for more information: <https://journals.plos.org/plosmedicine/s/revising-your-manuscript#loc-author-summary>

[Response] We have now added the following short Author Summary:

**Why was this study done?**

- In addition to being currently FDA-approved for the treatment of partial seizures and postherpetic neuralgia, gabapentin is extensively used off-label for many conditions, including neuropathic pain, fibromyalgia, anxiety, and tremor.
- Despite the increasing number of patients receiving gabapentin prescriptions, little information is available on the safety of this medication during pregnancy.
- We therefore evaluated the association between the use of gabapentin exposure during pregnancy and the risk of a range of neonatal and maternal outcomes.

**What did the researchers do and find?**

- We conducted a large population-based cohort study and used several strategies to minimize potential confounding and misclassification of the exposure and the outcome.
- Our results showed that gabapentin exposure during the first trimester of pregnancy was not associated with an increased risk of major malformations overall, though a moderate increase in the risk of cardiac malformations could not be excluded. Maternal use of gabapentin, particularly late in pregnancy, was associated with an increased risk of preterm birth, small for gestational age, and neonatal intensive care unit admission.

13. Methods: Thank you for your note (1st paragraph under “Source of Data and Study Population”) that the details of building the study cohort were previously published. Please however provide some relevant details, in particular which US states were not represented, and the fact that LMP was estimated (and how) as it seems like this could potentially impact interpretations regarding pregnancy timepoints.

[Response] We now report information on excluded US states and LMP estimation:

Methods, page 6, 2^nd^ paragraph: “Using the MAX dataset, we collected data for 46 US states and the District of Columbia during the period 2000 through 2013. Montana and Connecticut were excluded because of difficulty in linking data for mothers and infants, Michigan was excluded because of incomplete data, and data from Arizona were not available. The details of the strategy used to build the study cohort have been previously reported. The study population included all pregnancies resulting in live births among Medicaid-enrolled women 12 to 55 years old, who had continuous eligibility in Medicaid starting from three months prior to the estimated last menstrual period (LMP) to one month after delivery. The date of LMP was estimated based on the date of delivery combined with diagnostic codes for preterm delivery, using a validated algorithm [Margulis AV, Setoguchi S, Mittleman MA, Glynn RJ, Dormuth CR, Hernandez-Diaz S. Algorithms to estimate the beginning of pregnancy in administrative databases. Pharmacoepidemiology and drug safety 2013;22:16-24].”

14. Methods: How was race/ethnicity defined and by whom? Why was race/ethnicity considered important in this study and what it is believed to represent?

[Response] Race/ethnicity was categorized based on information submitted to the Centers for Medicare & Medicaid Services by individual states. Race/ethnicity was considered important in this study as it could represent a potentially important confounding factor. We have added the following statement in the Methods section:

Methods, page 8, 2^nd^ paragraph: “Race or ethnicity was considered to represent a potentially important confounding factor, and was categorized based on information submitted to the Centers for Medicare & Medicaid Services by individual states.”

15. Methods: Sensitivity analyses: In the first scenario, you restrict analyses to greater or equal to two gabapentin prescriptions filled during each period to reduce the potential for exposure misclassification. Can you please clarify what is meant by exposure misclassification more explicitly? In the discussion, the interpretation is that such individuals may use gabapentin more consistently.

[Response] We have included the assumption motivating the sensitivity analysis restricted to patients filling ≥2 gabapentin prescriptions during each period:

Methods, page 10, 1^st^ paragraph: “We conducted several sensitivity analyses to assess the robustness of our findings. First, in order to reduce the potential for exposure misclassification, we updated the exposure definition as filling ≥2 gabapentin prescriptions during each of the previously specified periods of interest, with the assumption that filling multiple prescriptions would increase the likelihood that gabapentin was actually taken or taken consistently.”

16. Results: Please note the comment from Reviewer 3 regarding the presentation of the unadjusted results in the text. If presenting both, please consistently describe the adjusted or unadjusted in the text for all outcomes. In the tables, please present both the unadjusted and PS-adjusted results for all measures.

[Response] We agree with Reviewer 3 and do not report unadjusted results, except for primary analyses (Table 2). For primary analyses, we report unadjusted results in order to show the direction and the magnitude of the shift in the risk estimate with confounding adjustment. We believe this step facilitates the interpretation of the adjusted results. Once direction and magnitude of confounding adjustment are established and adjusted primary results are shown, we would prefer not to report unadjusted estimates for secondary analyses that aim to test the robustness of primary findings, as we agree with the reviewer these may be distracting and potentially misleading. However, if the Editor prefers unadjusted results to be included also in the table summarizing results for secondary analyses, we are happy to do so.

As unadjusted results are presented in Table 2, we now describe in detail unadjusted results in the text as suggested by the Editor:

Results, page 12, 2^nd^ paragraph: “Before PS adjustment, gabapentin was significantly associated with an increased risk of preeclampsia [RR, 1.17 (1.01-1.35) for early exposure; RR, 1.43 (1.03-2.00) for late exposure; RR, 1.52 (1.23-1.87) for early and late exposure], preterm birth [RR, 1.46 (1.35-1.57) p<0.001 for early exposure; RR, 1.84 (1.55-2.18) for late exposure; RR, 1.93 (1.73-2.16) for early and late exposure], SGA [RR, 1.71 (1.50-1.96) for early exposure; RR, 1.97 (1.43-2.71) for late exposure; RR, 2.26 (1.85-2.75) for early and late exposure], and NICU admission [RR, 1.89 (1.73-2.07) for early exposure; RR, 2.11 (1.69-2.63) for late exposure; RR, 3.03 (2.69-3.41) for early and late exposure].”

17. Results: As also alluded to by a reviewer, please revise the final sentence of the first paragraph under “Absolute and relative risks of neonatal and maternal outcomes” to: “The relative risks were no longer statistically significant after PS adjustment…”

[Response] We propose the following wording:

Results, page 12, 1^st^ paragraph: “After PS adjustment, the relative risks moved to RR, 1.07 (0.94-1.21) and RR, 1.12 (0.89-1.40), respectively.”

18. Results: For relative risks associated with all congenital and cardiac malformations, please provide both the 95% CIs and p values associated.

[Response] Please see our previous response regarding reporting p-values.

19. Results: In the second paragraph under “Absolute and relative risks of neonatal and maternal outcomes”: please revise to “In PS-adjusted analyses, there was no statistically significant increase in risk of preeclampsia regardless…” Please also present the risk with 95% CIs and p values associated. For associations with preterm birth, small for gestational age, and NICU admissions, please also present the p values for the PS-adjusted findings.

[Response] We have revised the sentence in line with our previous response regarding reporting p-values :

Results, page 13, 1^st^ paragraph: “In PS-adjusted analyses, there was no increase in risk of preeclampsia regardless of the timing of the gabapentin exposure during pregnancy [RR, 0.87 (0.75-1.00) for early exposure; RR, 0.96 (0.69-1.33) for late exposure; RR, 0.92 (0.74-1.13) for early and late exposure].”

20. Results: Under “Sensitivity, secondary and post-hoc analyses”, please remove the word meaningful and revise the sentence to read: “No significant associations between gabapentin exposure and individual non-cardiac malformations were identified, and elevated point estimates observed for central nervous system defects, ear anomalies, and non-cardiac vascular defects were not statistically significant (eTable6).”

[Response] We have revised the sentence as follows:

Results, page 14, 1^st^ paragraph: “No significant associations between gabapentin exposure and individual non-cardiac malformations were identified,…”

21. Discussion: Please clarify or remove the word “meaningful” from the following sentence (second paragraph, on page 12): “...allowed us to rule out meaningful increases in the risk of overall major malformations among pregnancies exposed to gabapentin during the first trimester with higher precision…”

[Response] We have revised the sentence and removed the word “meaningful”.

22. Discussion: Please present and organize the Discussion as follows: a short, clear summary of the article's findings; what the study adds to existing research and where and why the results may differ from previous research; strengths and limitations of the study; implications and next steps for research, clinical practice, and/or public policy; one-paragraph conclusion. Specifically, a discussion of implications and next steps relevant for clinical practice is missing.

[Response] We have included the following discussion of implications and next steps relevant for clinical practice:

Discussion, page 18, 1^st^ paragraph: “Our results add to the current understanding of the safety of gabapentin prenatal use and provides pregnant women with pain conditions and epilepsy and their providers important information, which can guide clinical decisions during pregnancy. Our findings also indicate that pregnant women using gabapentin during pregnancy may be considered for targeted interventions to monitor for and promptly respond to the potential adverse outcomes associated with the use of this agent.”

23. Conclusions: Please revise the first sentence of the conclusion to temper the implications that can be drawn, we suggest: “Results from this study suggest that gabapentin exposure during early pregnancy does not appear to be associated with teratogenic effects.” or similar.

[Response] We have revised as suggested:

Conclusions, page 18, 2^nd^ paragraph: “Results from this large cohort study suggest that gabapentin exposure during early pregnancy does not appear to be associated with strong teratogenic effects.”

24. Tables: Tables should be numbered 1-3 rather than 1, 3, and 4.

[Response] We have revised accordingly.

25. Table 1: Please define the abbreviation for “SD” in the legend.

[Response] We now provide the definition for “SD”.

26. Table 3: Please define abbreviation for T1. Please provide p values in addition to 95% CIs for all outcomes.

[Response] We now define the abbreviation T1.

27. Table 4: Please define abbreviations for T1, Rx, dx. Please provide p values in addition to 95% CIs for all outcomes. Please also provide the unadjusted results.

[Response] We now provide definitions for the above abbreviations. As previously discussed in response to Reviewer 3 and the Editor, we would prefer not to include unadjusted results in order not to distract from the main message of the Table. However, if the Editor prefers unadjusted results to be included in this table, we are happy to do so.

28. Figure 2: Please define Pc0.

[Response] The definition of Pc0 is provided in the Figure footnote:

Figure 2, footnote: “Assuming (1) a prevalence of maternal smoking among unexposed pregnancies (Pc0) of approximately 30%[1], …”

29. eTable3, eTable4: Please define abbreviation for SD.

[Response] We now provide the definition for “SD” in eTable 3 and 4.

30. eTable5: Please define “2 Rx” in the legend. Please provide p values in addition to 95% CIs.

[Response] We now define “Rx” in the legend.

31.eTable6: Please define “2 Rx” and T1 in the legend. Please provide p values in addition to 95% CIs.

[Response] We now define “Rx” in the legend. We have substituted the abbreviation “T1” in the table and used instead “first trimester”.

32. eTable 7: Please define “T1” in the legend. Please provide p values in addition to 95% CIs. Please also provide the results of the unadjusted analyses.

[Response] We now define “T1” in the legend. As previously discussed in response to Reviewer 3 and the Editor, we would prefer not to include unadjusted results in order not to distract from the main message of the Table. However, if the Editor prefers unadjusted results to be included in this table, we are happy to do so.

33. eFigure1: Please define the abbreviation for “RR” in the legend.

[Response] We now define “RR” in the legend.

34. References: Please use square brackets for in-text citations, like this: [1]. Please use the "Vancouver" style for reference list formatting, and see our website for other reference guidelines: <https://journals.plos.org/plosmedicine/s/submission-guidelines#loc-references>

[Response] We have reformatted citations accordingly.

35. Checklist: Please ensure that the study is reported according to the STROBE guideline, and include the completed STROBE checklist as Supporting Information. When completing the checklist, please use section and paragraph numbers, rather than page numbers. Please add the following statement, or similar, to the Methods: "This study is reported as per the Strengthening the Reporting of Observational Studies in Epidemiology (STROBE) guideline (S1 Checklist)."

[Response] We have included the completed STROBE checklist as supporting information and have added the following statement in the Methods:

Methods, page 6, 2^nd^ paragraph: “A STROBE checklist and a description of our planned analyses are included in the supplemental material.”

Comments from the reviewers:

Reviewer #1: Thank you very much for allowing me to review this article on the use of Gabapentin in Pregnancy and the Risk of Neonatal and Maternal Outcomes. This is an important topic where safety information is lacking to support clinical management and policy decision making. The article is well written and the approach well reported thereby aiding transparency that is commendable. Please find my comments below for consideration that mainly relate to the methods and results section.

[Response] Thank you for the positive feedback.

1) "Continuous Medicaid eligibility was also required among the linked infants for a minimum of three months after birth".

Could the authors describe why 3 months following birth was chosen to ascertain outcomes in neonates. Because there is an increased risk of pre-term birth with gabapentin-exposed patients meaning the period for outcome detection in infants born at term will be 40 weeks +12 weeks compared to e.g. pre-term 36 weeks +12 weeks, what is the possibility of differential outcome ascertainment of congenital anomalies in offspring? Is there an increased likelihood for example of detecting a cardiac defect if offspring are more likely to be admitted to the NICU etc.?

[Response] Thanks for the comment. We chose 3 months following birth to ascertain outcomes in neonates as major congenital malformations are generally detected within 3 months after birth. We did not observe an increase in the risk of preterm or NICU among the women exposed to gabapentin early in pregnancy, i.e., the relevant etiologic period for congenital malformations, and thus we do not expect meaningful differential ascertainment of malformations within this population.

2) "Pregnancies with exposure to acknowledged teratogenic agents during the first trimester were excluded"

A minor point but there is a risk in misunderstanding this sentence as meaning the exclusion of all teratogenic agents. I suggest clarifying that certain teratogens were still included but handled differently (i.e. some anticonvulsants).

[Response] Thanks for the suggestion. As the reviewer notes, we excluded pregnancies exposed to acknowledged teratogens, and balanced suspected teratogens through PS adjustment. We have revised the text accordingly in the Methods section:

Methods, page 8, 2^nd^ paragraph: “We considered a large number of potential confounders: maternal age at delivery, …, concomitant use of medications (e.g., opioids and specific morphine equivalents, other anticonvulsant medications, angiotensin-converting enzyme (ACE) inhibitors), …”

3) The first trimester is defined as the first 90 days of pregnancy. Exposure early in pregnancy is defined as the first 140 days of pregnancy. By definition all exposed individuals in the first trimester should appear in the exposure early in pregnancy cohort (the latter of which is likely to be the larger of the two). The results however, report the number of exposed patients in the first trimester as 4,642 and the number of patients exposed early in pregnancy as 3,745, which seems implausible if I have correctly understood the proposed definition. Please could the authors clarify.

[Response] Exposure during the first trimester (T1) was defined as pregnancies with at least one filled prescription for gabapentin during the first 90 days of pregnancy, regardless of exposure to gabapentin later in pregnancy. Exposure early in pregnancy was defined as at least one gabapentin dispensing in the first 140 days of pregnancy and no dispensing between the 141st and 245th day. We have edited the Methods section as follows:

Methods, page 7, 1^st^ paragraph: “First trimester (T1) exposure was defined as pregnancies with at least one filled prescription for gabapentin during the first 90 days of pregnancy (starting from the date of LMP)*,* independently of exposure to gabapentin later in pregnancy.”

4) A minor point but for clarity in the covariates section of the methods I would add in brackets what indications for gabapentin are included.

[Response] We have added main gabapentin indications in the section:

Methods, page 8, 2^nd^ paragraph: “We considered a large number of potential confounders: maternal age at delivery, …, indications for gabapentin (e.g., epilepsy or seizure, neuropathic pain), other pain conditions, psychiatric disorders, …”

5) It is quite common that some lifestyle information is missing in electronic databases. Was there any missing data on smoking and lifestyle information? If so, how was this handled?

[Response] Administrative claims databases, such as MAX, are regarded to contain complete information on all diagnoses, procedures and filled medications, as they are used as the basis for healthcare billing, so there are no missing data in the traditional sense in these databases. However, certain important patient characteristics, e.g., smoking and other lifestyle factors are known to be under-recorded in claims databases, which could cause residual confounding.

To mitigate possible residual confounding we conducted specific sensitivity analyses, i.e., hdPS-adjustment, the restriction to women with either epilepsy/seizures or pain indications, and the extension of the covariate assessment period through the first 140 days of pregnancy, which produced results consistent with the main analyses. We also conducted a bias analysis to quantify the potential bias associated with a maternal smoking for preterm birth, SGA, and preeclampsia (protective), which revealed that adjusted results were fairly robust even under extreme scenarios of imbalance between exposed and unexposed pregnancies (see Figure 2).

6) "For the primary analyses, we measured maternal comorbidities and concomitant medication use during the 3 months before pregnancy through the end of the first trimester."

Data on maternal comorbidities and concomitant medication were used to estimate the propensity for treatment with gabapentin. However, the propensity score is a subject's probability of treatment, conditional on observed baseline covariates. Including covariate information up to the end of the first trimester means post-baseline data can be used to estimate the propensity score. In many instances gabapentin may have been prescribed before or early in pregnancy it is possible that this population may have more baseline covariate information. What proportion of covariate information included in estimating the propensity score was present at the start of pregnancy between the groups and how might this affect the propensity score distributions? Could the authors discuss potential bias implications in using this approach?

[Response] Thanks for the comment. We purposely measured maternal comorbidities and concomitant medication through the end of the first trimester in order to capture as much information as possible on existing medical conditions for exposed and unexposed women. Because of the many routine checks and examinations related to a new pregnancy, information on baseline medical conditions have more opportunity to be recorded during the first trimester, independently of gabapentin exposure. Our propensity score was estimated only of the basis of chronic conditions or treatments that are not expected to be causal intermediates between gabapentin and pregnancy complications. On the other hand, as measures of healthcare utilization, e.g., physician visits, etc., could be causal intermediates between gabapentin and pregnancy complications, only information on these measures through LMP was included in the PS model.

We have revised the text as follows:

Methods, page 8, 2^nd^ paragraph: “For the primary analyses, we measured maternal comorbidities and concomitant medication use during the 3 months before pregnancy through the end of the first trimester. Information deriving from the first trimester was included in the definition of these covariates, because information on existing medical conditions, which are not expected to be causal intermediates between gabapentin exposure and pregnancy complications, have more opportunity to be recorded during the first trimester, due to the routine checks and examinations related to a new pregnancy.”

7) "We trimmed the population whose PS fell within the non-overlapping areas of the PS distributions, and created 50 PS-strata according to the distribution of the exposed pregnancies."

Could a figure demonstrating the degree of overlap in the PS distributions be reported to help better understand how generalizable the findings may be?

[Response] Thanks for the suggestion. We have added a figure describing the overlap in the PS distributions in the Appendix (eFigure 1), where we also report the percentage of gabapentin exposed women retained in the analysis after trimming. We have included the following text in the Methods section:

Methods, page 9, 2^nd^ paragraph: “We trimmed the population whose PS fell within the non-overlapping areas of the PS distributions, and created 50 PS-strata according to the distribution of the exposed pregnancies. Due to the large overlap in the PS distribution of gabapentin exposed and unexposed women, all exposed women across the four exposure groups, except for one exposed to gabapentin late in pregnancy, were retained in the analysis after trimming (see eFigure 1).”

8) The article reports some statistically significant associations between the use of gabapentin and cardiac malformations. The study also evaluates the risk of maternal outcomes using a cohort consisting of exposed late in pregnancy. Given this cohort of exposed late in pregnancy has already been established could the authors consider using it to test for potential residual confounding in the association with cardiac malformations if gabapentin exposure late in pregnancy may act as a negative control?

[Response] Thanks for the comment. The size of the cohort of women exposed to gabapentin late in pregnancy was almost 10 times smaller (556 women) than the size of the cohort exposed to gabapentin during T1 (4642 women), making it a challenging negative control for a rare outcome such as cardiac malformations. Specifically, if we evaluate the association between exposure to 2 or more filled prescriptions of gabapentin in this cohort and the risk of cardiac malformations, we find a RR = 0.47 (95% CI, 0.07-3.48), p=0.48, which is not sufficiently informative to be interpreted as a negative control.

9)  It may be interesting to examine whether the association with preterm delivery is consistent or varies by the main indications (i.e. epilepsy vs. chronic pain) in case the associated problems with chronic pain and its management influence the timing of delivery.

[Response] We agree and examine the associations with preterm deliveries among subgroups defined by epilepsy and pain in Table 3. These subgroup analyses produced results consistent with the primary findings.

Reviewer #2: This paper tackles an extremely important issue, as the use of gabapentin during pregnancy has increased dramatically over recent years.

This is an excellent paper examining if gabapentin exposure during pregnancy increases the risk of neonatal maternal outcomes. The methodology is well thought out, with many additional sensitivy analyses included to confirm their results and is very carefully described in the paper. I really enjoyed reading this paper  and can think of no imporvemens to it. It is an exceptional piece of work.

[Response] Thanks, we appreciate the positive feedback.

Reviewer #3: This study of the risks associated with gabapentin use during pregnancy is based on a retrospective analysis of a large claims database and on propensity score stratification to control for confounders when comparing women exposed vs not exposed to gabapentin. I found the methods appropriate and well described including a range of sensible sensitivity analyses. I also found the discussion and conclusion well written and reflecting the uncertainty due to potential residual confounding and multiplicity. I only have minor comments, mostly aimed at clarifying the presentation, listed below:

*	Please indicate the design of the study in the title. For example: "Gabapentin in Pregnancy and the Risk of Neonatal and Maternal Outcomes: A Propensity-Matched retrospective Cohort Study"

[Response] We have updated the title as “Gabapentin in Pregnancy and the Risk of Adverse Neonatal and Maternal Outcomes: A Population-based Cohort Study”.

*	The data statement suggests that all data necessary to replicate the analysis is available without restriction; however, this does not seem to be the case. Only aggregate results are available in the supplement but not raw data. Given the nature of the data (US Claims data), I suspect that the authors would not have the authority to make the raw data available. This needs to be clarified.

[Response] We have included the following Data Availability Statement in the manuscript: “Because of the data use agreement in place, the research team cannot share the database used for the current paper, which was based on Medicaid, a joint federal and state program that helps provide health care coverage for people with low incomes and limited resources in the United States. Other researchers may request to gain access to the Medicaid database through the Research Data Assistance Center (ResDAC) (<https://www.resdac.org/>)”

*	First trimester (T1) exposure (90 days) and early pregnancy exposure (first 140 days) appear very similar and I wonder whether we need them both. Is it mainly because early pregnancy exposure excludes exposures after day 140 while T1 exposure does not exclude exposure after Day 90. Please confirm and clarify the rationale.

[Response] As the relevant etiologic period for major malformations is the first trimester, i.e., the first 90 days of pregnancy, assessing exposure to gabapentin beyond the first trimester (through day 140) may introduce exposure misclassification and thus would not allow for a valid ascertainment of its teratogenic effects. On the other hand, the potential impact of gabapentin on placentation and other aspects that may affect other neonatal or maternal complications could continue beyond the first 90 days of pregnancy. Thus, for these outcomes, limiting the exposure assessment period to the first 90 days of pregnancy may undermine the correct quantification of their association with gabapentin exposure. For these reasons, we believe both periods are important to correctly estimate the association of gabapentin with the range of outcomes included in this manuscript.

*	Please also clarify why exposure is limited to Day 245 and not later. In general, I would like to better understand the rationale for the different cutoffs chosen to define the exposure periods.

[Response] We assessed late gabapentin exposure only through Day 245, to exclude the possibility of differential opportunity to be exposed to gabapentin between preterm and full-term pregnancies.

*	Given how closely the exposure periods overlap, please clarify for selecting different outcomes for T1 exposure vs early pregnancy exposure.

[Response] Please the Authors’ discussion two responses above regarding why we believe both periods are important to correctly estimate the association of gabapentin with the range of outcomes included in this manuscript.

*	For the baseline characteristics listed in Table 1, eTable 3 and eTable 4, please clarify the corresponding measuring period when not clear i.e. in particular, please indicate whether the characteristic is measured before the pregnancy and, if so, the length of the "recall" period (e.g. in the 3 months preceding pregnancy) or during pregnancy or both. I note that this is quite well defined in the methods; however, adding details to the tables themselves might help.

[Response] Thanks for the suggestion. We now include the following text in the tables’ footnotes:

Table 1, eTable 3, and eTable 4: “Maternal comorbidities and concomitant medication use were measured during the 3 months before pregnancy through the end of the first trimester. Measures of healthcare intensity (e.g., number of medical visits) were measured only during the 3-month period before pregnancy, in order for these not to be affected by early awareness of possible pregnancy complications.”

*	In the results, I would suggest not reporting the unadjusted results as we know that before PS adjustments, the groups are very different and the comparisons potentially misleading. I am not sure I would include unadjusted results in the tables unless the goal is to demonstrate the importance of adjusting.

[Response] We agree with the reviewer and do not report unadjusted results, except for primary analyses (Table 2). For these analyses, we report unadjusted results in order to show the direction and the magnitude of confounding adjustment. We believe this step improves the interpretation of the adjusted results themselves. Once direction and magnitude of confounding adjustment are established and adjusted primary results are shown, we agree with the reviewer that reporting unadjusted estimates for sensitivity and subgroup analyses that aim to test the robustness of primary findings, would be distracting and potentially misleading.

*	Results section, absolute and relative risks of neonatal and maternal outcomes: In the sentence "The relative risks were no longer meaningfully elevated after PS adjustment [RR, 1.07 (0.94-1.21); RR, 1.12 (0.89-1.40)]." I would suggest changing the wording to "statistically significant" instead of "meaningfully elevated" and clarify what PS "adjustment" mean by using more specific terminology e.g. "PS stratification and weighting".

[Response] We propose the following wording:

Results, page 12, 1^st^ paragraph: “After PS adjustment, the relative risks moved to RR, 1.07 (0.94-1.21) and RR, 1.12 (0.89-1.40), respectively.

For consistency with Tables 2 and 3, we would prefer to keep the wording “PS adjustment” in the text and refer the reader to the Methods section for details related to the adjustment strategy.

*	Table 4 includes many numbers, which makes it difficult to identify patterns (e.g. significant or inconsistent results). I would suggest using a forest plot instead of a table. This applies to some of the tables in the supplement too.

[Response] We appreciate the reviewer’s suggestion, though we think the addition of forest plots on top of the information already reported in Table 4 and other Tables in the Appendix (RR and 95% CI), would make tables very busy and difficult to read. However, if the Editor prefers forest plots to be included in Table 4 and other Tables in the Appendix, we are happy to do so.

*	Please consider including plots showing the propensity score distribution before adjustment in the supplement.

[Response] Thanks for the suggestion. We have added a figure describing the PS distributions of gabapentin exposed and unexposed women in the Appendix (eFigure 1) before adjustment. In the same figure, we also report the percentage of gabapentin exposed women retained in the analysis after trimming.

*	When looking at the effect of gabapentin according to dose tertiles, please clarify whether each tertile has been separately adjusted using a new propensity score. To allow each tertile to be compared to the unexposed group, I would expect that a separate propensity score be calculated for each tertile as the propensity is likely to change as the dose increase. Please confirm that this is the approach used. An potential alternative to study a dose-response relationship is to perform an analysis according to tertiles of the propensity score itself.

[Response] That is correct, we re-estimated PS within each dose tertiles.

*	Is it possible to add confidence bands to Figure 2 and eFigure 1?

[Response] We appreciate the reviewer’s suggestion, though we think the addition of confidence intervals to the figure would impair a clear visualization of the two figures. The main message of the two figures is that primary estimates remain consistent despite potential bias or differences in the proportion of terminations among women exposed to gabapentin versus those unexposed, and we think the addition of confidence intervals could be distracting. However, if the Editor prefers confidence intervals to be included in the figures, we are happy to do so.

*	Please include the STROBE checklist as well as the statistical analysis plan in the supplement.

-Laurent Billot

[Response] We now include both the STROBE checklist and the statistical analysis plan in the Appendix.

Reviewer #4: General comments

The authors present a large register-based study on the use of gabapentin in pregnancy and risk of congenital malformations and other important pregnancy and neonatal outcomes.

This author group has strong and well-documented credentials with the field as witnessed by this manuscript. The study is compelling in its scope, execution and reporting. This manuscript substantially advances knowledge on the use of gabapentin in pregnancy  and provide much needed decision support to physicians and pregnant women.

That being said, there are some minor issues that I believe should be clarified, justified and discussed prior to publication.

[Response] Thank you for this positive feedback.

Specific comments

Introduction

Short sharp and to the point. The sentence "As gabapentin actively crosses…" is trivial (all but very few drugs cross placenta, and I am not sure if the term "actively" is accurate) could be omitted with no loss of information.

[Response] Thanks for the suggestion. We have omitted the sentence.

Methods

Well described. I would like clarification/justification/discussion of the following points:

a)	Exposure window. The authors have chosen to eliminate the 30-day window prior to conception. This is fine, but I suggest they discuss the possible consequences thereof and elaborate on the typical prescription coverage of gabapentin (1 month? Three months?)

[Response] As most prescriptions in the U.S. are for 30 days, the exposure definition required a gabapentin filled prescription during the first 90 days of pregnancy, to limit potential exposure misclassification. This implies we might have missed some exposed pregnancies that may have filled their prescription during the 30 days prior to conception. This does not introduce misclassification since pregnancies exposed to gabapentin prior to conception are removed from the reference group.

b)	Exclusion criteria. The authors exclude some known teratogenic drugs. However, some quite notable drugs are omitted, especially ACE-inhibiting drugs and antiepileptics with documented increased risk of major congenital malformations: valproic acid, carbamazepine and phenytoin (I am aware that these latter exposures are included in the PS model). The authors should discuss.

[Response] Thanks for the comment. As the reviewer notes, we excluded pregnancies exposed to acknowledged teratogens, and balanced suspected teratogens through PS adjustment. We have revised the text in the Methods section to clarify this aspect:

Methods, page 8, 2^nd^ paragraph: “We considered a large number of potential confounders: maternal age at delivery, …, concomitant use of medications (e.g., opioids and specific morphine equivalents, other anticonvulsant medications, angiotensin-converting enzyme (ACE) inhibitors), …”

c)	Exposure definition. Please state the definition of the start of pregnancy explicitly (LMP?).

[Response] We now explicitly state that LMP corresponded to the start of pregnancy:

Methods, page 7, 1^st^ paragraph: “First trimester (T1) exposure was defined as pregnancies with at least one filled prescription for gabapentin during the first 90 days of pregnancy (starting from the date of LMP)*,* independently of exposure to gabapentin later in pregnancy.”

d)	Outcome assessment. Please discuss the possible consequences of a short follow-up (3 months). Some - especially cardiac - malformations are detected later in the first year after birth.

[Response] Most major malformations are detected within the first 3 months after birth, so we do not expect meaningful consequences deriving from a 3-month follow-up.

Nevertheless, in a sensitivity analysis, we have restricted to infants continuously eligible for at least 1 year and extended infant follow-up for cardiac and overall malformations ascertainment to 1 year (See table below).

| **Cardiac malformations** | **Unexposed** | **Exposed**  **during T1** |
| --- | --- | --- |
| **Primary analyses, infants continuously eligible for ≥ 3 months, using a 3-month follow-up for cardiac malformations ascertainment:** |  | **PS-adjusted  RR (95% CI)** |
| Main PS-adjusted analysis | Ref. | 1.12 (0.89-1.40) |
|  |  |  |
| **Restricted to infants continuously eligible for ≥ 1 year, using a 1-year follow-up for cardiac malformations ascertainment:** |  |  |
| Main PS-adjusted analysis | Ref. | 1.10 (0.88-1.37) |
|  |  |  |
| **Major congenital malformations** | **Unexposed** | **Exposed**  **during T1** |
| **Primary analyses, infants continuously eligible for ≥ 3 months, using a 3-month follow-up for malformations ascertainment:** |  | **PS-adjusted  RR (95% CI)** |
| Main PS-adjusted analysis | Ref. | 1.07 (0.94-1.21) |
|  |  |  |
| **Restricted to infants continuously eligible for ≥ 1 year, using a 1-year follow-up for malformations ascertainment:** |  |  |
| Main PS-adjusted analysis | Ref. | 1.05 (0.94-1.18) |

Results from these analyses, which are based on a smaller population of exposed and unexposed pregnancies (approximately 80% of the original cohort size), show consistency with the results of our primary analyses.

Results for the 1-year follow-up are now included in **Table 3** and discussed in the text:

Methods, page 10, 1^st^ paragraph: “Second, to ensure that maternal malformations were not being erroneously attributed to the offspring, and thus reduce the chances of outcome misclassification, we re-defined the outcome of major malformations using infant claims only and extended infant follow-up to 1 year, for infants continuously eligible for Medicaid for ≥1 year.”

Discussion, page 17, 1^st^ paragraph: “We also re-defined the malformation outcomes using infant claims only and extended follow-up to 1 year for infants continuously eligible for ≥1 year, which confirmed the primary results.”

e)	Covariates. While I am confident that the following has been elaborately published elsewhere, I believe that this information is quite important for the reader to have at hand as these covariates are important:

Alcohol, overweight and smoking: Please briefly describe how these were assessed and stratified and list the % of missing data on these covariates in the original MAX dataset.

[Response] Administrative claims databases, such as MAX, are regarded to contain complete information on all diagnoses, procedures and filled medications, as they are used as the basis for healthcare billing. However, certain important patient characteristics, e.g., smoking and other lifestyle factors are under-recorded in claims databases. Because of this, although we included claims-based definitions of smoking, alcohol, and overweight/obesity in the PS model, residual confounding by these factors is possible.

To mitigate possible residual confounding by these factors, we built a PS based on over 70 potential confounders and proxies for unmeasured confounders in the main analyses, and conducted specific sensitivity analyses aiming at addressing residual confounding, i.e., hdPS-adjustment, the restriction to women with either epilepsy/seizures or pain indications, and the extension of the covariate assessment period through the first 140 days of pregnancy. These produced results consistent with the main analyses.

We have also conducted a bias analysis to quantify the potential bias associated with a maternal smoking for preterm birth, SGA, and preeclampsia (protective), which revealed that adjusted results were fairly robust even under extreme scenarios of imbalance between exposed and unexposed pregnancies (see Figure 2).

f)	Inferential analysis for risk of preterm birth. I believe the authors should discuss the possibility of immortal time bias in this context.

[Response] We acknowledge the reviewer’s point. In order to exclude the possibility of differential opportunity to be exposed to gabapentin between preterm and full-term pregnancies, we specifically assessed late gabapentin exposure only through Day 245. Immortal time bias therefore would not have affected these analyses.

g)	Bias analysis (smoking). I acknowledge the reference to the assumed Pc0 prevalence of 30% maternal smoking, but such seem extremely high to me still. Please elaborate.

[Response] Smoking prevalence is generally higher in the Medicaid population compared to less disadvantaged populations. In addition, as only the unexposed women that resemble more closely those exposed to gabapentin will fall within the same stratum of the propensity score and will contribute to the analyses, it is expected that these unexposed women will have higher smoking prevalence compared to the unexposed women that do not contribute to the analyses.

Results

Clearly presented if a somewhat long narrative section.

Discussion

Clear and well substantiated in the actual findings. Some adjustments may be warrented pending the response to the issues above.
