# Supplementary material for: Gabapentin in pregnancy and the risk of adverse neonatal and maternal outcomes: A population-based cohort study nested in the US Medicaid Analytic eXtract dataset
Source: PLoS Med. 2020 Sep 1;17(9):e1003322. doi: 10.1371/journal.pmed.1003322 (PMC7462308; doi:10.1371/journal.pmed.1003322)
Supplement: Attachment — Submitted filename: Response_Gabapentin_PLOS_Med_Apr 10 2020_final.docx [file pmed.1003322.s017.docx]

***Elisabetta Patorno, M.D., Dr.P.H. Assistant Professor of Medicine***

***epatorno@bwh.harvard.edu***

Requests from the editors:

1.Abstract (and throughout): Thank you for your responses to this query in the previous round of revision, and we appreciate your willingness to consider the inclusion of p values for your results. Please provide p values in addition to 95% CIs.

[Response] We appreciate the Editor and Reviewer #3 point of view regarding the inclusion of p-values. We agree that the p-value quantifies the degree of consistency between the observed data and the null hypothesis, and that the real issue is its misinterpretation. However, we feel that we should not include p-values in the current manuscript for the following reasons:

- The confidence interval helps to frame what the full p-value function looks like, and thus illustrates the degree of consistency between the observed data and *all possible hypotheses*, not just the null hypothesis

- Presenting the p-value brings the focus back to the null (it quantifies the consistency between the observed data and the *null hypothesis*), which makes it in essence a significance testing exercise

- Once you provide the confidence interval (as we do), the p-value is redundant. It can be calculated by the reader based on the confidence interval

For these reasons, we strongly feel it is not desirable to provide the p-values in our manuscript; it invites misinterpretation and encourages the tendency to do (implicit) significance testing. Our Division has recently taken a strong stance against reporting p-values in manuscripts.

If the Editor still feels that reporting p-values is necessary, we would kindly ask for the opportunity to have a dedicated conference call to better contextualize our request.

2. Author summary: Thank you for including an author summary. Please also include the section: “What Do These Findings Mean?” (In this section, authors should reflect on the new knowledge generated by the research and the implications for practice, research, policy, or public health. Authors should also consider how the interpretation of the study’s findings may be affected by the study limitations.) Please see our author guidelines for more information on formatting this section: <https://journals.plos.org/plosmedicine/s/revising-your-manuscript#loc-author-summary>

[Response] We have now included the section “What Do These Findings Mean?”:

**What Do These Findings Mean?**

Pregnant women and their physicians should weigh the benefits of treatment with gabapentin with the risks of potential adverse pregnancy outcomes associated with its use.

3. Methods: “Source of Data and Study Population”: Please provide a reference for the US Medicaid Analytic eXtract (MAX) database used.

[Response] We have included a reference for the US Medicaid Analytics eXtract (MAX):

12. Centers for Medicare and Medicaid Services. Medicaid Analytic eXtract (MAX) General Information. https://www.cms.gov/Research-Statistics-Data-and-Systems/Computer-Data-and-Systems/MedicaidDataSourcesGenInfo/MAXGeneralInformation. Accessed April 8, 2020.

4. Methods: “Source of Data and Study Population”: Thank you for including the STROBE checklist. Please revise the last sentence to read: This study is reported as per the Strengthening the Reporting of Observational Studies in Epidemiology (STROBE) guideline (S1 Checklist)." or similar.

[Response] We have revised the sentence accordingly:

Methods, page 6, 2^nd^ paragraph: “A description of our planned analyses is included in the supplemental material (see Appendix). This study is reported as per the Strengthening the Reporting of Observational Studies in Epidemiology (STROBE) guideline (STROBE Checklist, Appendix)”

5. Results section, throughout: We thank you for your responses on this subject, but please do report p values to accompany the 95% CIs presented for all results. In instances where you report “significant” associations, please indicate whether the intended meaning is “statistical significance”.

[Response] Please see our response to Editorial point 1.

6. Results: “Absolute and relative risks of neonatal and maternal outcomes”: Please provide p values along with 95% CIs for all presented analyses, unadjusted and adjusted, throughout this section. We suggest you change “the relative risks moved” to “the relative risks attenuated” or similar in the sentence “After PS adjustment, the relative risks moved to RR, 1.07 (0.94-1.21) and RR, 1.12 (0.89-1.40), respectively.” to enhance clarity.

[Response] Please see our response to Editorial point 1 regarding the inclusion of p values.

We propose to revise the sentence as:

Results, page 12, 2^nd^ paragraph: “After PS adjustment, the relative risks were RR, 1.07 (0.94-1.21) and RR, 1.12 (0.89-1.40), respectively.”

7. Results “Sensitivity, secondary and post-hoc analyses”: Please provide p values along with 95% CIs for all presented analyses.

[Response] Please see our response to Editorial point 1.

8. Results: (and response to editor comment #16): Thank you for your response to this, please do report (in the table) the unadjusted results for the secondary analyses.

[Response] As requested by the Editor, we provide below a version of the table reporting both PS-adjusted and unadjusted results. As mentioned in our previous rebuttal letter, we are in agreement with Reviewer #3 and are similarly concerned that reporting unadjusted analyses for sensitivity and secondary analyses may be distracting and potentially misleading. The inclusion of unadjusted results makes the table very hard to read (as shown below) and, we believe, defeats its main purpose, i.e. testing the robustness of primary PS-adjusted (not unadjusted) results. We would therefore suggest to keep the current version of the table and only report PS-adjusted results. If the Editor still feels that include both PS-adjusted and unadjusted results is preferable, we propose to include the table below in the Appendix.

**Table 3. Sensitivity and secondary analyses for the relative risk of neonatal and maternal outcomes associated with exposure to gabapentin compared with unexposed pregnancies**

| **Exposure Group:** | **Unexposed** | **Exposed**  **during T1** | **Exposed  early in pregnancy** | **Exposed  late in pregnancy** | **Exposed  early and late in pregnancy** |
| --- | --- | --- | --- | --- | --- |
| **Outcomes** |  | **PS-adjusted  RR (95% CI)** | **PS-adjusted  RR (95% CI)** | **PS-adjusted  RR (95% CI)** | **PS-adjusted  RR (95% CI)** |
| **Major congenital malformations** |  |  |  |  |  |
| Main unadjusted analysis | Ref. | 1.49 (1.31-1.69) |  |  |  |
| Main PS-adjusted analysis | **Ref.** | **1.07 (0.94-1.21)** | . | . | . |
| ≥ 2 Rx, unadjusted | Ref. | 1.65 (1.37-1.99) |  |  |  |
| ≥ 2 Rx, PS-adjusted | Ref. | 1.15 (0.95-1.39) | . | . | . |
| Infant claims, unadjusted | Ref. | 1.52 (1.33-1.73) |  |  |  |
| Infant claims, PS-adjusted | Ref. | 1.05 (0.92-1.20) | . | . | . |
| 1-year follow-up^1^, unadjusted | Ref. | 1.51 (1.34-1.69) |  |  |  |
| 1-year follow-up^1^, PS-adjusted | Ref. | 1.05 (0.94-1.18) |  |  |  |
| High-dimensional-PS-adjusted | Ref. | 1.06 (0.93-1.20) | . | . | . |
| ≥ 1 epilepsy or seizure dx, unadjusted | Ref. | 1.22 (0.81-1.84) |  |  |  |
| ≥ 1 epilepsy or seizure dx, PS-adjusted | Ref. | 1.01 (0.66-1.56) | . | . | . |
| No epilepsy or seizure dx, unadjusted | Ref. | 1.46 (1.28-1.67) |  |  |  |
| No epilepsy or seizure dx, PS-adjusted | Ref. | 1.07 (0.93-1.22) | . | . | . |
| ≥ 1 pain dx^2^, unadjusted | Ref. | 1.33 (1.15-1.53) |  |  |  |
| ≥ 1 pain dx^2^, PS-adjusted | Ref. | 1.06 (0.91-1.22) | . | . | . |
| No pain dx, unadjusted | Ref. | 1.50 (1.16-1.96) |  |  |  |
| No pain dx, PS-adjusted | Ref. | 1.14 (0.87-1.49) | . | . | . |
| **Cardiac malformations** |  |  |  |  |  |
| Main unadjusted analysis | Ref. | 1.77 (1.43-2.18) |  |  |  |
| Main PS-adjusted analysis | **Ref.** | **1.12 (0.89-1.40)** | . | . | . |
| ≥ 2 Rx, unadjusted | Ref. | 2.44 (1.85-3.22) |  |  |  |
| ≥ 2 Rx, PS-adjusted | Ref. | 1.40 (1.03-1.90) | . | . | . |
| Infant claims, unadjusted | Ref. | 1.88 (1.52-2.34) |  |  |  |
| Infant claims, PS-adjusted | Ref. | 1.13 (0.89-1.44) | . | . | . |
| 1-year follow-up^1^, unadjusted | Ref. | 1.70 (1.36-2.12) |  |  |  |
| 1-year follow-up^1^, PS-adjusted | Ref. | 1.10 (0.92-1.53) |  |  |  |
| High-dimensional-PS-adjusted | Ref. | 1.11 (0.88-1.40) | . | . | . |
| High-dimensional-PS-adjusted, ≥2 Rx^3^ | Ref. | 1.40 (1.03-1.90) | . | . | . |
| ≥ 1 epilepsy or seizure dx, unadjusted | Ref. | 1.85 (1.02-3.37) |  |  |  |
| ≥ 1 epilepsy or seizure dx, PS-adjusted | Ref. | 1.40 (0.73-2.71) | . | . | . |
| No epilepsy or seizure dx, unadjusted | Ref. | 1.67 (1.34-2.09) |  |  |  |
| No epilepsy or seizure dx, PS-adjusted | Ref. | 1.08 (0.84-1.38) | . | . | . |
| ≥ 1 pain dx^2^, unadjusted | Ref. | 1.50 (1.18-4.12) |  |  |  |
| ≥ 1 pain dx^2^, PS-adjusted | Ref. | 1.07 (0.82-1.40) | . | . | . |
| No pain dx, unadjusted | Ref. | 2.05 (1.37-3.07) |  |  |  |
| No pain dx, PS-adjusted | Ref. | 1.32 (0.84-2.06) | . | . | . |
| **Preeclampsia** |  |  |  |  |  |
| Main unadjusted analysis | Ref. |  | 1.17 (1.01-1.35) | 1.43 (1.03-2.00) | 1.52 (1.23-1.87) |
| Main PS-adjusted analysis | **Ref.** | . | **0.87 (0.75-1.00)** | **0.96 (0.69-1.33)** | **0.92 (0.74-1.13)** |
| ≥ 2 Rx, unadjusted | Ref. |  | 1.45 (1.16-1.82) | 1.54 (0.85-2.80) | 1.46 (1.11-1.90) |
| ≥ 2 Rx, PS-adjusted | Ref. | . | 0.98 (0.78-1.23) | 1.17 (0.64-2.14) | 0.87 (0.67-1.14) |
| High-dimensional-PS-adjusted | Ref. | . | 0.86 (0.75-1.00) | 0.96 (0.69-1.33) | 1.00 (0.80-1.25) |
| Updated CAP^4^, unadjusted | Ref. |  | 1.17 (1.02-1.35) | 1.43 (1.03-2.00) | 1.52 (1.23-1.87) |
| Updated CAP^4^, PS-adjusted | Ref. |  | 0.88 (0.76-1.02) | 0.98 (0.70-1.36) | 0.93 (0.75-1.15) |
| ≥ 1 epilepsy or seizure dx, unadjusted | Ref. |  | 0.88 (0.46-1.68) | 1.05 (0.27-4.06) | 1.16 (0.65-2.07) |
| ≥ 1 epilepsy or seizure dx, PS-adjusted | Ref. | . | 0.74 (0.39-1.41) | 1.43 (0.37-5.46) | 0.82 (0.46-1.46) |
| No epilepsy or seizure dx, unadjusted | Ref. |  | 1.18 (1.02-1.36) | 1.44 (1.03-2.03) | 1.52 (1.21-1.92) |
| No epilepsy or seizure dx, PS-adjusted | Ref. | . | 0.88 (0.76-1.01) | 0.96 (0.68-1.35) | 0.96 (0.76-1.20) |
| ≥ 1 pain dx^2^, unadjusted | Ref. |  | 1.11 (0.95-1.30) | 1.13 (0.71-1.80) | 1.44 (1.14-1.83) |
| ≥ 1 pain dx^2^, PS-adjusted | Ref. | . | 0.91 (0.77-1.06) | 0.83 (0.52-1.31) | 0.94 (0.74-1.19) |
| No pain dx, unadjusted | Ref. |  | 1.00 (0.73-1.37) | 1.72 (1.07-2.76) | 1.24 (0.76-2.03) |
| No pain dx, PS-adjusted | Ref. | . | 0.74 (0.54-1.02) | 1.18 (0.74-1.89) | 0.84 (0.51-1.38) |
| **Preterm delivery** |  |  |  |  |  |
| Main unadjusted analysis | Ref. |  | 1.46 (1.35-1.57) | 1.84 (1.55-2.18) | 1.93 (1.73-2.16) |
| Main PS-adjusted analysis | **Ref.** | . | **1.00 (0.93-1.08)** | **1.28 (1.08-1.52)** | **1.22 (1.09-1.36)** |
| ≥ 2 Rx, unadjusted | Ref. |  | 1.56 (1.37-1.77) | 1.89 (1.38-2.59) | 2.02 (1.77-2.30) |
| ≥ 2 Rx, PS-adjusted | Ref. | . | 0.98 (0.86-1.12) | 1.27 (0.93-1.74) | 1.28 (1.12-1.46) |
| High-dimensional-PS-adjusted | Ref. | . | 1.01 (0.93-1.08) | 1.25 (1.06-1.49) | 1.22 (1.08-1.37) |
| Updated CAP^4^, unadjusted | Ref. |  | 1.46 (1.35-1.57) | 1.84 (1.55-2.18) | 1.93 (1.73-2.16) |
| Updated CAP^4^, PS-adjusted | Ref. |  | 0.99 (0.92-1.06) | 1.24 (1.04-1.47) | 1.20 (1.08-1.34) |
| ≥ 1 epilepsy or seizure dx, unadjusted | Ref. |  | 1.31 (0.99-1.74) | 1.41 (0.76-2.60) | 1.56 (1.20-2.02) |
| ≥ 1 epilepsy or seizure dx, PS-adjusted | Ref. | . | 1.07 (0.81-1.42) | 1.14 (0.51-2.51) | 1.47 (1.13-1.91) |
| No epilepsy or seizure dx, unadjusted | Ref. |  | 1.43 (1.33-1.55) | 1.83 (1.53-2.84) | 1.87 (1.66-2.11) |
| No epilepsy or seizure dx, PS-adjusted | Ref. | . | 1.00 (0.93-1.08) | 1.30 (1.09-1.55) | 1.20 (1.06-1.35) |
| ≥ 1 pain dx^2^, unadjusted | Ref. |  | 1.27 (1.16-1.37) | 1.71 (1.38-2.12) | 1.80 (1.60-2.03) |
| ≥ 1 pain dx^2^, PS-adjusted | Ref. | . | 0.96 (0.88-1.04) | 1.24 (1.00-1.54) | 1.22 (1.08-1.38) |
| No pain dx, unadjusted | Ref. |  | 1.54 (1.33-1.79) | 1.75 (1.33-2.31) | 1.61 (1.24-2.08) |
| No pain dx, PS-adjusted | Ref. | . | 1.15 (0.99-1.34) | 1.33 (1.01-1.75) | 1.19 (0.92-1.54) |
| **Small for gestational age (SGA)** |  |  |  |  |  |
| Main unadjusted analysis | Ref. |  | 1.71 (1.50-1.96) | 1.97 (1.43-2.71) | 2.26 (1.85-2.75) |
| Main PS-adjusted analysis | **Ref.** | . | **1.17 (1.02-1.33)** | **1.39 (1.01-1.91)** | **1.32 (1.08-1.60)** |
| ≥ 2 Rx, unadjusted | Ref. |  | 1.88 (1.50-2.36) | 1.99 (1.09-3.63) | 2.22 (1.74-2.84) |
| ≥ 2 Rx, PS-adjusted | Ref. | . | 1.15 (0.92-1.45) | 1.35 (0.74-2.46) | 1.28 (1.00-1.63) |
| High-dimensional-PS-adjusted | Ref. | . | 1.17 (1.02-1.33) | 1.36 (0.99-1.88) | 1.39 (1.12-1.72) |
| Updated CAP^4^, unadjusted | Ref. |  | 1.71 (1.50-1.96) | 1.97 (1.43-2.71) | 2.26 (1.85-2.75) |
| Updated CAP^4^, PS-adjusted | Ref. | . | 1.15 (1.01-1.32) | 1.36 (0.99-1.88) | 1.31 (1.08-1.60) |
| ≥ 1 epilepsy or seizure dx, unadjusted | Ref. |  | 1.53 (0.93-2.50) | 0.55 (0.08-3.78) | 1.65 (1.01-2.69) |
| ≥ 1 epilepsy or seizure dx, PS-adjusted | Ref. | . | 1.35 (0.83-2.20) | 1.03 (0.15-7.10) | 1.46 (0.89-2.38) |
| No epilepsy or seizure dx, unadjusted | Ref. |  | 1.68 (1.46-1.93) | 2.05 (1.48-2.84) | 2.21 (1.78-2.74) |
| No epilepsy or seizure dx, PS-adjusted | Ref. | . | 1.17 (1.01-1.34) | 1.46 (1.05-2.02) | 1.30 (1.05-1.62) |
| ≥ 1 pain dx^2^, unadjusted | Ref. |  | 1.56 (1.34-1.81) | 1.77 (1.17-2.68) | 2.08 (1.67-2.59) |
| ≥ 1 pain dx^2^, PS-adjusted | Ref. | . | 1.16 (1.00-1.35) | 1.33 (0.88-2.01) | 1.29 (1.04-1.61) |
| No pain dx, unadjusted | Ref. |  | 1.54 (1.16-2.06) | 1.96 (1.18-3.25) | 1.93 (1.24-3.03) |
| No pain dx, PS-adjusted | Ref. | . | 1.14 (0.85-1.51) | 1.44 (0.86-2.39) | 1.32 (0.84-2.07) |
| **NICU admission** |  |  |  |  |  |
| Main unadjusted analysis | Ref. |  | 1.89 (1.73-2.07) | 2.11 (1.69-2.63) | 3.03 (2.69-3.41) |
| Main PS-adjusted analysis | **Ref.** | . | **1.01 (0.93-1.11)** | **1.21 (0.97-1.51)** | **1.35 (1.20-1.52)** |
| ≥ 2 Rx, unadjusted | Ref. |  | 2.31 (1.99-2.67) | 1.87 (1.19-2.92) | 3.22 (2.79-3.71) |
| ≥ 2 Rx, PS-adjusted | Ref. | . | 1.08 (0.93-1.25) | 1.00 (0.64-1.57) | 1.43 (1.24-1.65) |
| High-dimensional-PS-adjusted | Ref. | . | 1.01 (0.92-1.11) | 1.19 (0.95-1.49) | 1.31 (1.15-1.50) |
| Updated CAP^4^, unadjusted | Ref. |  | 1.89 (1.73-2.07) | 2.11 (1.69-2.63) | 3.03 (2.69-3.41) |
| Updated CAP^4^, PS-adjusted | Ref. | . | 1.00 (0.91-1.09) | 1.16 (0.93-1.45) | 1.33 (1.18-1.50) |
| ≥ 1 epilepsy or seizure dx, unadjusted | Ref. |  | 0.88 (0.55-1.41) | 0.29 (0.04-2.03) | 1.59 (1.12-2.27) |
| ≥ 1 epilepsy or seizure dx, PS-adjusted | Ref. | . | 0.68 (0.42-1.11) | 0.31 (0.05-2.15) | 1.18 (0.83-1.68) |
| No epilepsy or seizure dx, unadjusted | Ref. |  | 1.93 (1.75-2.11) | 2.23 (1.78-2.79) | 3.11 (2.74-3.53) |
| No epilepsy or seizure dx, PS-adjusted | Ref. | . | 1.04 (0.95-1.14) | 1.29 (1.03-1.61) | 1.38 (1.22-1.56) |
| ≥ 1 pain dx^2^, unadjusted | Ref. |  | 1.55 (1.39-1.72) | 1.80 (1.35-2.40) | 2.56 (2.24-2.93) |
| ≥ 1 pain dx^2^, PS-adjusted | Ref. | . | 0.95 (0.86-1.06) | 1.13 (0.85-1.50) | 1.30 (1.13-1.48) |
| No pain dx, unadjusted | Ref. |  | 2.05 (1.71-2.46) | 2.12 (1.49-3,02) | 2.95 (2.29-3.82) |
| No pain dx, PS-adjusted | Ref. | . | 1.18 (0.99-1.42) | 1.33 (0.94-1.90) | 1.56 (1.21-2.01) |
| T1: first trimester; PS: propensity score; RR: risk ratios; CI: confidence intervals; NICU: neonatal intensive care unit; Ref.: reference; Rx: filled prescription; dx: diagnosis; CAP: covariate assessment period  ^1^ Restricted to infants continuously eligible for ≥ 1 year ^2^ Includes neuropathic pain, fibromyalgia, arthritis, arthropathies and musculoskeletal pain, back and neck pain, migraine or headache, osteoarthritis, rheumatoid arthritis, or other pain  ^3^ Post-hoc analysis ^4^ CAP measured from the last menstrual period through the first 140 days of pregnancy | | | | | |

9. Figure 2: In the title, please include panels E and F (...unexposed and gabapentin exposed women in either late (A, C, E) or both early and late in pregnancy (B, D, F)), or remove the panel IDs from the title as this information is found in the legend.

[Response] Thank you for the suggestion. We have chosen to remove the panel IDs from the title that now reads as “**Figure 2. Bias analysis quantifying the impact on point estimates of increasing residual differences in the prevalence of maternal smoking between unexposed and gabapentin exposed women in either late or both early and late in pregnancy**”

10. eFigure 1: Please use a consistent number of decimal places for the x axes across panels. In the legend, please provide a description of the comparison groups: Exp_Group: exposure group; Gaba_Group1-4: gabapentin exposed women in exposure groups 1 to 4 (presumably gaba = gabapentin, exposure groups 1-4 correspond to time periods of exposures). Please include a descriptive figure legend, if possible.

[Response] Thank you. We now provide a consistent number of decimal places for the x axes and include a descriptive figure legend.

11. eTable 5: Please provide p values.

[Response] Please see our response to Editorial point 1.

References: Please check the formatting for reference 3, removing the trademark symbol. Please double check that your references use the "Vancouver" style for reference formatting (it looks like you may need a period between

the journal names and the publication year), and see our website for other reference guidelines <https://journals.plos.org/plosmedicine/s/submission-guidelines#loc-references>

[Response] Thank you, we have adjusted the formatting of the references.

12. Checklist: Thank you for providing your STROBE checklist. Please revise the checklist, using sections and paragraphs, rather than page numbers, to refer to locations throughout the manuscript.

[Response] Thank you, we have revised the checklist and now only refer to sections and paragraphs.

Comments from the reviewers:

Reviewer #1: Thank you for asking me to review the revision. The responses are satisfactory. I am clearer that a patient's complete claim history prior to the pregnancy is not available. For this reason information accruing throughout the first trimester is the only way to identify the necessary information. Although it would normally be expected that key past medical history is recorded early on, I agree that more medical conditions could have chance to accumulate over time. The primary propensity score analysis was estimated only on the basis of chronic conditions or treatments that are not expected to be causal intermediates between gabapentin and pregnancy complications, which is valid and the variables are listed in etable 3. Assuming the high dimensional propensity score (HDPS) uses more covariates than simply those in etable 3, the HDPS could be more at risk from the appearance of conditions and treatments after gabapentin exposure has commenced. However, this is a

secondary analysis and given the effect estimates are similar to those of the primary PS analysis it seems unlikely to be an issue here.

[Response] Thank you for the comment. We agree that HDPS could be more at risk of including potential causal intermediates, due to the empirical nature of the underlying variable selection. However, as the reviewer notes, HDPS-adjusted results were almost identical to the primary PS-adjusted results, indicating a low likelihood for causal intermediates to have been included in the analysis.

Reviewer #3: Looking at eFigure1 which shows the overlap in PS distribution, it looks as if 100% (or very close to 100%) of the unexposed group fell within the first bar of the histogram whereas, for the exposed groups, the proportion included in the first bar varied between approximately 65% and 92%. This indicates a substantial lack of overlap in the propensity score distributions between the exposed and non-exposed groups, yet the authors report retaining 100% (or 99.8%) of exposed cases.

[Response] Thank you for the comment. The plots illustrated in eFigure 1 report the overlap in PS distribution for the 4 exposure groups after trimming, before weighting. Because weighting was not applied to generate these plots, the scale of the y-axes for PS distributions of exposed (in red) and unexposed (in blue) is not directly comparable. The PS distribution of unexposed (in blue) refers to a sample of over 1.7 million pregnancies, whereas the PS distribution of exposed (in red) refers to a much smaller sample, ranging between a few hundreds and a few thousands pregnancies. While it is true that a percentage close to 100% of unexposed pregnancies have small PS probabilities, due to the large number of unexposed women (over 1.7 million), in absolute terms there are still many unexposed pregnancies with PS probabilities as large as the PS probabilities of exposed pregnancies. However, as the percentage of unexposed pregnancies with large PS probability is low (relatively to the 1.7 million unexposed population), it is difficult to appreciate their presence in the section of the plot illustrating larger PS probabilities. Conversely, the PS distribution of exposed pregnancies (in red) appears more clearly in these sections, because the percentage of exposed pregnancies with large PS probability (relatively to the exposed population) is higher than the percentage of unexposed pregnancies with large PS probability (relatively to the unexposed population)

We understand the current version of the plots may be misleading for readers, thus, we propose to replace these plots with plots generated after trimming and after weighting, which may more clearly illustrate the overlap of the two PS distributions on a comparable scale. We provide below an example (based on exposure group 1) of how updated plots look like:

Please clarify the rule used for trimming and the reason for having so few cases trimmed.

[Response] We trimmed the population whose PS fell within the non-overlapping areas of the PS distributions. Because the sample size of unexposed pregnancies was so large (over 1.7 million), it was possible to find unexposed patients with overlapping propensity scores for almost all exposed pregnancies, as reported in eFigure 1.

This lack of PS overlap suggests that a number of exposed cases would have been matched (i.e. included in the same strata) as non-exposed cases who had a much lower propensity score. I wonder about the possible biases induced by including exposed cases with high (non-overlapping) propensity scores and whether a sensitivity analysis excluding exposed cases who have a "non-overlapping" propensity score might alleviate those concerns.

[Response] Please see our previous responses regarding the overlap of PS distributions. Only exposed and unexposed pregnancies with overlapping PS were included in adjusted analyses.

I note that the authors do not wish to report p-values. While p-values can indeed be misinterpreted, I feel that they help quantify the degree of consistency between the observed data and the hypotheses and are a useful complement to confidence intervals. In fact, the articles cited, e.g. the ASA statement, do not necessarily ask authors to avoid using p-values but rather warn against misinterpretation. For the sake of transparency and interpretation, I would encourage the reporting of p-values in addition to confidence intervals but will leave the decision with the editors.

[Response] Please see our response to Editorial point 1.

Reviewer #4: The authors have satisfactorily addressed issues raised in my review and revised the manuscript accordingly.

[Response] Thank you for the positive feedback.
