# Supplementary material for: Gabapentin in pregnancy and the risk of adverse neonatal and maternal outcomes: A population-based cohort study nested in the US Medicaid Analytic eXtract dataset
Source: PLoS Med. 2020 Sep 1;17(9):e1003322. doi: 10.1371/journal.pmed.1003322 (PMC7462308; doi:10.1371/journal.pmed.1003322)
Supplement: Attachment — Submitted filename: Response_Gabapentin_PLOS_Med_July 1 2020_bb.docx [file pmed.1003322.s018.docx]

Requests from the editors:

1. Response to reviewers/editors: Thank you for the responses to the reviewer and editor comments.

- Response to Editorial Request #1:“We have asked on previous occasions for p values to be provided alongside 95% CIs. These are still not provided and I should advise you that we will be unwilling to continue with this paper unless you provide them. We understand the limitations of presenting and interpreting p-values that you have outlined in your response letter. However, our readers expect to see them, and thus PLOS Medicine requires both p-values and 95% CIs when presenting results. We will withdraw the paper from our files if these are not provided on the resubmission.

[Response] We now provide the p-values.

- Response to Editorial Request #8: Please present the results from the unadjusted analyses in addition to the adjusted analyses as requested previously (in a separate table would be acceptable).

[Response] We now provide unadjusted results in eTable 5.

2. Title: Please include the population and setting (country)- we suggest “Gabapentin in pregnancy and the risk of adverse neonatal and maternal outcomes: A population-based cohort study of the US Medicaid Analytic eXtract (MAX) dataset”

[Response] We have updated the Title as follows: “Gabapentin in Pregnancy and the Risk of Adverse Neonatal and Maternal Outcomes: A Population-based Cohort Study nested in the US Medicaid Analytic eXtract Dataset.”

3. Abstract: Methods and Findings: Please include some summary demographic information on the individuals included in the dataset and please include the months along with the years for the included pregnancies.

[Response] We now provide some summary demographic information and the months and years for the included pregnancies.

4. Abstract: Methods and Findings: Please provide p-values in addition to 95% CIs.

[Response] We have added the p-values.

5. Abstract: Methods and Findings: Can you please clarify this sentence: “There was no association with preeclampsia after adjustment” to indicate which relationship you are describing?

[Response] We have updated the sentence as:

Abstract, Methods and Findings: “There was no higher risk of preeclampsia among women exposed to gabapentin after adjustment”

6. Results: Last sentence of “Study cohort and patient characteristics”- does the superscript 19 indicate reference 19? If so please place the reference in square brackets: [19].

[Response] Thanks for the comment. This superscript meant to refer to reference 20. We now report the correct reference number in square brackets: [20].

7. Results: “Absolute and relative risks of neonatal and maternal outcomes”: Please provide p-values associated with the risk ratios and 95% CIs here, and throughout the manuscript. In the methods, please specify the significance level used (eg, P<0.05, two-sided) and the statistical test used to derive a p-value.

[Response] We have added 95% CI and p-values throughout the manuscript. Please see the e-mail correspondence with Dr. Moyer regarding the agreed upon language for describing the findings.

8. Results: “Sensitivity, secondary and post-hoc analyses”: For the following sentence, please clarify whether the risk was significantly increased for the subgroup of patients with an epilepsy/seizure diagnosis: “Of note, the risk of cardiac malformations in pregnancies exposed to gabapentin during T1 was significantly increased when we re-defined the exposure based on ≥2 filled prescriptions [RR, 1.40 (1.03-1.90)], and remained elevated in a post-hoc analysis with hdPS adjustment [RR, 1.40 (1.03-1.90)], and in a subgroup analysis that restricted to patients with a recorded diagnosis of epilepsy or seizures [RR, 1.40; 0.73-2.71)].”

[Response] In line with the criteria used to interpret our findings, we have revised the sentence as:

Results, page 13, 2^nd^ paragraph: “Of note, the risk of cardiac malformations in pregnancies exposed to gabapentin during T1 was increased when we re-defined the exposure based on ≥2 filled prescriptions [RR, 1.40 (1.03-1.90), p=0.03], and in a post-hoc analysis with hdPS adjustment [RR, 1.40 (1.03-1.90), p=0.03], and remained apparently elevated, with a wider CI, in a subgroup analysis that restricted to patients with a recorded diagnosis of epilepsy or seizures [RR, 1.40; 0.73-2.71), p=0.31].”

9. Discussion: Last sentences of paragraph beginning “This study has limitations”: We suggest revising to: “Our results add to the current understanding of the safety of gabapentin prenatal use and provide pregnant women with pain conditions and epilepsy and their providers with important information, which can guide clinical decisions during pregnancy. Our findings also suggest that pregnant women using gabapentin during pregnancy may be considered for targeted interventions to monitor for and promptly respond to the potential adverse outcomes associated with the use of this agent.”

We also suggest that the discussion of the strengths and limitations of the study be a separate paragraph from the discussion of implications and next steps for research.

[Response] Thank you for the comment. We have revised the sentences as suggested and included them in a separate paragraph.

10. Conclusions: The caveat regarding the impact of multiple analyses on the cardiac finding would be more appropriately discussed in the paragraph describing the study’s limitations. “A moderate increase in the risk of cardiac malformations – in particular conotruncal defects – cannot be excluded, although in the context of multiple analyses the possibility of a chance finding should be taken into consideration.” In the last sentence, please change “clinicians need to” to “clinicians should” or similar.

[Response] We now discuss the impact of multiple analyses in the limitations:

Discussion, page 18, 1^st^ paragraph: “Fifth, in the context of multiple analyses the possibility of a chance finding should be taken into consideration.”

We have also revised the Conclusions as follows:

Conclusions, page 18, 1^st^ paragraph: “Results from this large cohort study suggest that gabapentin exposure during early pregnancy does not appear to be associated with teratogenic effects, though a moderately higher risk of cardiac malformations – in particular conotruncal defects – cannot be excluded. Maternal use of gabapentin, particularly late in pregnancy, was associated with an increased risk of preterm birth, SGA, and NICU admission; an association that was only partially explained by confounders. Clinicians should weigh these potential risks with the clinical benefits of using gabapentin to treat painful and disabling conditions.”

11. Table 2 and Table 3, and eTable 5, 6, and 7: Please provide p values associated with these results.

[Response] We now provide p-values in all tables.

Comments from the reviewers:

Reviewer #3: My previous query about the apparent lack of overlap in PS distribution has been adequately addressed. I have no further comment.

-Laurent Billot

[Response] Thank you for letting us know our response adequately addressed the Reviewer’s query.

**Requests from the editors on June 19, 2020:**

1. Please do provide all p values in addition to 95% CIs to accompany your results, as previously agreed.

[Response] These have been added, as requested.

2. As you prefer not to dichotomize results, please remove the term “significant” and all related terms and references throughout the manuscript.

[Response] These have been removed, as requested.

3. In the Abstract Methods and Findings, please change “There was an increase in risk of preterm birth among women exposed to gabapentin…” to “There was a higher risk of…”

[Response] This has been changed, as suggested.

4. In the Abstract Conclusions, please change “... we found that gabapentin exposure during early pregnancy did not appear to be associated with strong teratogenic effects, though a moderate increase in the risk of cardiac malformations could not be excluded.” to “.. we did not find evidence for an association between gabapentin exposure during early pregnancy and major malformations, though there was some evidence of a higher risk of cardiac malformations.”

[Response] This has been changed, as suggested.

5. In the Abstract Conclusions, please change “…Maternal use of gabapentin, particularly late in pregnancy, was associated with an increased risk of PTB, SGA, and NICUa.” To “…associated with a higher risk…”

 [Response] This has been changed, as suggested.

6. In the Author Summary, 5th point, please edit to “We did not find evidence of an association between gabapentin exposure during the first trimester of pregnancy and major malformations."

[Response] Author Summary, 5th point has been changed, to: “We did not find evidence of an association between gabapentin exposure during the first trimester of pregnancy and major malformations overall, although there was some evidence of a higher risk of cardiac malformations.”

7. In the Author Summary, 5th point, rather than “moderate increase in the risk of cardiac malformations could not be excluded” please rephrase as “there was some evidence of a higher risk of cardiac malformations”.

[Response] Author Summary, 5th point has been changed, to: “We did not find evidence of an association between gabapentin exposure during the first trimester of pregnancy and major malformations overall, although there was some evidence of a higher risk of cardiac malformations.”

8. In the Author Summary, 5th point, please edit “Maternal use of gabapentin, particularly late in pregnancy, was associated with an increased risk of preterm birth, small for gestational age, and neonatal intensive care unit admission.” To “There was a higher risk of preterm birth, small for gestational age, and neonatal intensive care unit admission among women exposed to gabapentin, particularly in late pregnancy.”

 [Response] This has been changed, as suggested.

9. In the Results section: Twice each on pages 12, 13, 14 of the PDF,  please delete “significant/significantly/non-significantly”.

[Response] These have been changed, as suggested.

10. In the Results, on p.13, 4-5 lines up from the end, you write “… and in a subgroup analysis restricted ...” and then quote an RR associated with CI that cross unity. Please change this to “… but not in a subgroup analysis restricted …”.

[Response] We have changed this sentence but prefer a slightly different wording. This has been changed to the following (incorporating the language suggested by Dr. Moyer on June 29, 2020):

“Of note, the risk of cardiac malformations in pregnancies exposed to gabapentin during T1 was increased when we re-defined the exposure based on ≥2 filled prescriptions [RR, 1.40 (1.03-1.90), p=0.03], and in a post-hoc analysis with hdPS adjustment [RR, 1.40 (1.03-1.90), p=0.03], and remained apparently elevated, with a wider confidence interval, in a subgroup analysis that restricted to patients with a recorded diagnosis of epilepsy or seizures [RR, 1.40; 0.73-2.71), p=0.31].”

11. In the Results, please rephrase the sentence near the top of page 14 " No significant associations between gabapentin exposure and individual..." to avoid mentioning significance.  We propose the following revision: “There was no evidence to support associations between gabapentin exposure and individual non-cardiac malformations, though point estimates were elevated for central nervous system defects, ear anomalies, and non-cardiac vascular defects.”

[Response] We have also changed this sentence but use different wording. This has been changed to the following:

“Elevated point estimates were observed for central nervous system defects, ear anomalies, and non-cardiac vascular defects, albeit with wide confidence intervals (eTable 7).”

12. In the second sentence of the Discussion, please change to: “We did not find evidence of a higher risk of major overall or cardiac malformations

following gabapentin exposure during the first trimester of pregnancy.”

13. In the Discussion, at the bottom of p.14, “… showed a 40% increased risk” seems rather stridently worded given the apparently modest number of events, please rephrase as “... found there may be a higher risk... ”

[Response for 12 &13] We have changed these sentences, as suggested, but have used wording that matches the conclusion from the Abstract:

“We did not find evidence for an association between gabapentin exposure during early pregnancy and major malformations overall, though there was some evidence of a higher risk of cardiac malformations; a sensitivity analyses restricted to women that may use gabapentin more consistently suggested there may be a higher risk of cardiac malformations, and a subsequent post-hoc analysis, which evaluated individual cardiac malformations, revealed a potential increase in the risk of conotruncal defects specifically.”
