# Supplementary material for: Gabapentin in pregnancy and the risk of adverse neonatal and maternal outcomes: A population-based cohort study nested in the US Medicaid Analytic eXtract dataset
Source: PLoS Med. 2020 Sep 1;17(9):e1003322. doi: 10.1371/journal.pmed.1003322 (PMC7462308; doi:10.1371/journal.pmed.1003322)
Supplement: Attachment — Submitted filename: Response_Gabapentin_PLOS_Med_July 23 2020.docx [file pmed.1003322.s019.docx]

Requests from the editors:

1.Title: Thank you for updating your title to: “Gabapentin in Pregnancy and the Risk of Adverse Neonatal and Maternal Outcomes: A Population-based Cohort Study nested in the US Medicaid Analytic eXtract Dataset.” Please also make sure this title is updated on the manuscript submission form/system to ensure that the title is carried forward accurately.

[Response] Thank you for the comment. We have now updated the title on the manuscript submission form/system.

2.Author summary: "Why was this study done?": Please remove the word “only” from the second bullet point.

[Response] We have removed the word “only” from the bullet.

3.Discussion: Conclusion paragraph: Please revise this sentence as follows, changing “increased” to “higher” to be consistent with wording elsewhere in the text: “Maternal use of gabapentin, particularly late in pregnancy, was associated with a higher risk of preterm birth, SGA, and NICU admission; an association that was only partially explained by confounders.”

[Response] We have updated the sentence as requested.

4.Sections titled “Author contributions”, “Funding”, “Competing interests”, and “Data availability statement”: These can be removed from the manuscript body; please be sure the information is entered into the relevant place in the manuscript submission form and it will automatically be included with the manuscript.

[Response] We have removed the sections as requested.

5.Appendix: Please include each document/table as a separate supporting information file.

[Response] We have included each document/table as a separate supporting information file.

Production Issues:

FIGURES and GRAPHICS:

1. Please upload your main figures with file type 'figure' and not file type 'other'. Please upload your supplementary figures with file type 'supporting information'.

[Response] Done as requested.

2. Please label figures in order of citation and as the appropriate file type (i.e., as "Figure" or "Supporting Information" and as "Fig 1, Fig 2, Fig 3," etc.).

[Response] Done as requested.

3. Some text in eFigure1 is below our recommended font size (8-12 pt) and difficult to read. Please remake the text in question at a larger size or take steps to make the text legible.

[Response] We have created an individual figure for each panel and increased the font size.

4. Graph images in eFigure1 appear pixelated/low quality. Please remake this part of the figure using the original source, using the PLOS figure guidelines to ensure quality is preserved [http://journals.plos.org/plosmedicine/s/figures].

[Response] We have recreated each of the four figures embedded in eFigure1 and created individual files for each of them. We also provide the TIF files for each of them.

5. Please remove embedded figures from your manuscript file and ensure the most up to date versions of your figures are uploaded to our submission system as separate TIF or EPS files. Please do not remove the figure legends or captions from the manuscript.

[Response] Done as requested.

6. Please ensure that all main figure files are clearly labeled in the File Inventory.

[Response] All main figure files are clearly labeled in the File Inventory

TABLES:

1. Please cite main tables in ascending numerical order in your main article file.

[Response] Main tables are cited in ascending order.

METADATA:

1. Please ensure your short title is correct in the submission form, including symbols, italicisation etc. Your short title will be typeset from the submission form directly.

[Response] The short title is correct.

2. Please accurately complete the 'Competing Interests' section on our submission form, including any COIs declared by your co-authors. If you have no competing interests to declare, please add "The authors have declared that no competing interests exist". Otherwise please declare all competing interests beginning with the statement "I have read the journal's policy and the authors of this manuscript have the following competing interests:"

[Response] We have completed the “Competing Interests” section as requested.

3. Please provide a detailed Financial Disclosure statement in the submission form. Include grant numbers and URLs as appropriate, as well as the initials of the authors who received the funding. This is published with the article, therefore it should be completed in full sentences and contain the exact wording you wish to be published.

Please add the statement 'The funders had no role in study design, data collection and analysis, decision to publish, or preparation of the manuscript' to the end of your Financial Disclosure statement on the submission form. If this statement is inaccurate, please describe the role the funders had.

If you received no funding, please add the statement 'The author(s) received no specific funding for this work' to your submission form.

[Response] We have provided a detailed Financial Disclosure statement.

4. In accordance with PLOS' data policy, please ensure that your Data Availability Statement in the submission form clearly identifies how readers can access your data. Data cannot be available on request, or only accessible by contacting one of the authors. Note that your Data Availability Statement will be typeset as it is written, so please ensure it is in complete sentences and appears as you would like it to in the published version.

For details on our data policy, please see <http://journals.plos.org/plosmedicine/s/data-availability>

[Response] We have provided a Data Availability Statement.

ARTICLE FILE:

1. Please indicate the corresponding author on the title page with an asterisk (*) and include the corresponding author's email address. Only an asterisk and the email address itself are required.

[Response] We now indicate the corresponding author on the title page with an asterisk (*) and report the corresponding author's email address.

2. Please remove author degree titles from the byline of your manuscript file.

[Response] We have removed the author degree titles.

3. Please ensure that all authors have at least one affiliation listed on the title page and that all listed affiliations are attributed to at least one author. Affiliations should be listed in ascending numerical order in the affiliation list and should match what is provided in the Authors Information in the submission form.

Affiliations should include an institution, a city, and a country (no postcode). US affiliations need the state as well, written out in full (with no abbreviations).

[Response] Affiliations now comply with the journal’s requests.

4. Please ensure that all author names are spelled correctly in the manuscript file and the submission form, including the use of a middle name/initials, and any special characters (accents, umlauts, etc.). Please place first/given names before last/family names. We ask that you ensure they match verbatim. Please be aware that correct spelling and formatting of author names in the article file is vital for accurate indexing in PubMed.

[Response] All authors names are spelled correctly.

5. Please ensure that your Ethics Statement is available in the Methods section of your manuscript in its entirety.

[Response] The Ethics Statement is available in the Methods section of the manuscript in its entirety.

6. We have noted that you have the following present in your manuscript: Financial Disclosure/ Competing Interests statement/ Author Contributions/ Data Availability Statement]

We have removed these section from your manuscript file as this information is automatically pulled from the submission form. Please make sure that the information in the submission form is correct.

[Response] Thanks, we have now removed these sections from the manuscript file.

REFERENCES:

1. Please make sure all references are cited in ascending numerical order in the text.

[Response] All references are cited in ascending numerical order.

SUPPORTING INFORMATION:

1. Please ensure that all supporting information files that are intended for publication have the file type 'Supporting Information' in the File Inventory.

[Response] Done as requested.

2. Please ensure your supporting information files are individually uploaded to the File Inventory as file type 'Supporting Information' and are not present in the manuscript file.

[Response] All supporting information files have been individually uploaded.

3. Please upload your Supporting Information items individually, rather than in a single file, as you refer to them individually in the text. Supporting Information files should be less than 10 MB each, and the individual figure files should be labelled clearly eg “S1 Fig”, "S1A Fig" etc.

[Response] All supporting information files have been individually uploaded and labeled as requested.

4. Please ensure that all Supporting Information files are clearly labled in the file inventory. i.e. S1_Fig.tif, S1_Table.xlsx, S1_Text.doc, S1_Data.xlsx, S1_STROBE_Checklist.docx, etc.

Each file type should be numbered starting at 1. Please also make sure that the file description matches the file name (e.g. S1_Fig.tif is listed as S1 Fig).

[Response] All supporting information files have been labeled as requested.

5. Please ensure that each supporting information file has a legend at the end of the manuscript file, after the Reference list.

[Response] We have now included a legend after the Reference list.

6. Please note that your submission cannot contain any references to generic "supporting information" files and instead must refer to specific files using our naming convention (S1 Fig, S1 Table, S1 Text, S1 Data, etc.)

[Response] We now refer to specific files using the requested naming convention.

7. Please replace any page numbers in your STROBE statement with paragraph numbers per section (e.g. "Methods, paragraph 1") since the page numbers of the final published paper may be different from the page numbers in the current manuscript.

[Response] All page numbers have been removed.
